# Zirconium tetraazamacrocycle complexes display extraordinary stability and provide a new strategy for zirconium-89-based radiopharmaceutical development†
†TJW, DNP and NB have filed patents relating to this work. All other authors declare no competing financial interests exist.
‡
‡Electronic supplementary information (ESI) available: Complete experimental details and supporting data. CCDC 1501174. For ESI and crystallographic data in CIF or other electronic format see DOI: 10.1039/c6sc04128k



**DOI:** 10.1039/c6sc04128k

**Published:** 2016-12-13

**Authors:** Darpan N. Pandya, Nikunj Bhatt, Hong Yuan, Cynthia S. Day, Brandie M. Ehrmann, Marcus Wright, Ulrich Bierbach, Thaddeus J. Wadas

**Affiliations:** a Department of Cancer Biology , Wake Forest School of Medicine , Winston-Salem , NC 27157 , USA . Email: twadas@wakehealth.edu ; Email: dapandya@wakehealth.edu; b Department of Radiology , University of North Carolina at Chapel Hill , Chapel Hill , NC 27599 , USA; c Department of Chemistry , Wake Forest University , Winston-Salem , NC 27109 , USA; d Department of Chemistry , University of North Carolina at Chapel Hill , Chapel Hill , NC 27599 , USA

## Abstract

We report our initial investigations into the use of tetraazamacrocycles as zirconium-89 chelators. We describe the synthesis and complete characterization of several Zr tetraazamacrocycle complexes, and definitively describe the first crystal structure of zirconium 1,4,7,10-tetraazacyclododecane-1,4,7,10-tetraacetic acid (Zr–DOTA) using single crystal X-ray diffraction analysis. After evaluating several radioactive analogs, we found that ^89^Zr–DOTA is superior to ^89^Zr–DFO, the only ^89^Zr-complex to be used clinically in ^89^Zr-radiopharmaceutical applications. Finally, we provide a rationale for the unanticipated and extraordinary stability of these complexes *in vitro* and *in vivo*. These results may inform the development of safer and more robust immuno-PET agents for precision medicine applications.

## Abstract 2

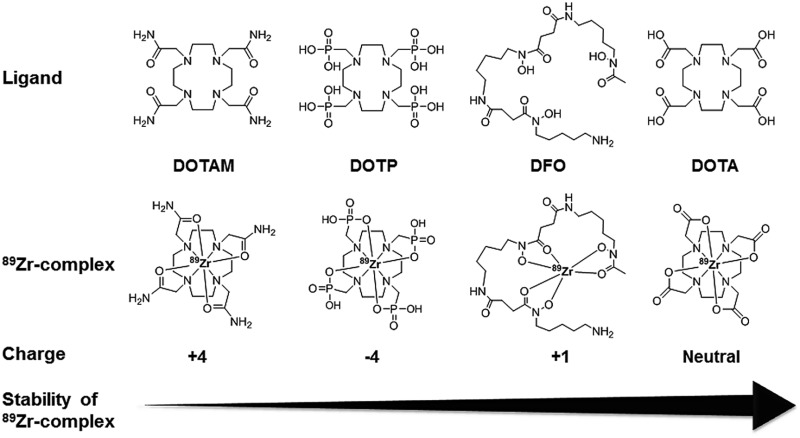
 ^89^Zr–Tetraazamacrocycle complexes display extraordinary stability.

## Introduction

Zirconium-89 (^89^Zr: (*t*_1/2_ = 78.4 h, β^+^: 22.8%, *E*_β+max_ = 901 keV; EC: 77%, *E*_γ_ = 909 keV)) is a positron-emitting radionuclide currently being tested in over 30 clinical trials involving monoclonal antibodies (mAbs); its radioactive half-life complements the biological half-life of a circulating antibody *in vivo*.^1^ Standard practice within the radiopharmaceutical industry requires ^89^Zr to be attached to an antibody through desferrioxamine (DFO) or its analogs, which are derivatives of the iron chelator desferral, a growth-promoting agent secreted by *Streptomyces pilosus*.^2,3^ Despite the widespread use of DFO in ^89^Zr-immuno-PET applications, the unsaturated coordination sphere of ^89^Zr–DFO is believed to be responsible for its observed instability in preclinical animal models,^4–6^ and significant effort has been expended to develop improved ^89^Zr(iv)-chelators.^7–21^

Tetraazamacrocycles such as 1,4,7,10-tetraazacyclododecane-1,4,7,10-tetraacetic acid (DOTA) remain a largely unexplored class of ligands due to a perceived inability to form stable ^89^Zr-complexes.^20,22–24^ Although only anecdotal evidence has appeared in the literature, instability is believed to arise from chelation by the four macrocycle nitrogen atoms and four oxygen atoms of the pendant arms rather than eight oxygen donors, which are believed to be preferred by the oxophilic ^89^Zr^4+^ ion.^23^ As a result, little is known regarding their Zr coordination chemistry or use in ^89^Zr-radiopharmaceutical development. Despite well-reasoned arguments to the contrary,^20,22–24^ it seemed reasonable to posit that tetraazamacrocycles would be useful in ^89^Zr-radiopharmaceutical applications, since several Zr–cyclam complexes have been described previously.^25,26^ Additionally, we reasoned that their use would be advantageous since (1) they demonstrate enhanced stability over acyclic ligands due to the macrocyclic effect; (2) various functional groups can be introduced into the macrocycle's backbone or pendant arms to modulate the ligand's stereo- and coordination chemistry; (3) bifunctional chelators derived from these ligands allow them to be conjugated to various peptides, proteins, and antibodies; and (4) they have been used successfully in a number of radiopharmaceutical applications and clinical trials.

**Fig. 1:**
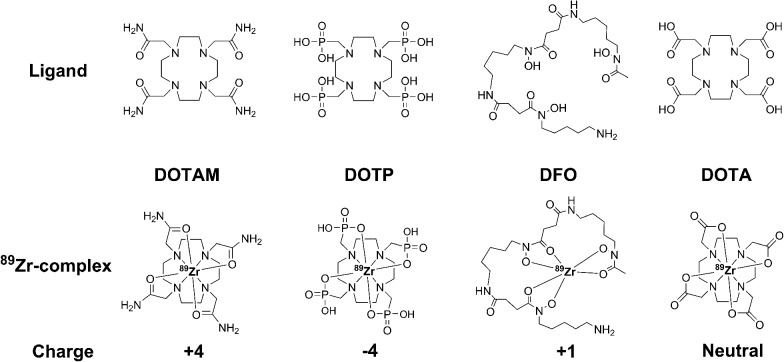
Structures of tetraazamacrocyclic ligands, desferral (DFO), and their ^89^Zr-complexes.

Here we document our initial investigations into the use of tetraazamacrocycles as ^89^Zr-chelators. We describe the synthesis and complete characterization of Zr–DOTA, Zr–DOTAM, and Zr–DOTP (Fig. 1), and describe the first crystal structure of Zr–DOTA, which reveals a saturated coordination sphere around the Zr^4+^ ion. Finally, we evaluate the radioactive analogs *in vitro* and *in vivo*, and show that ^89^Zr–DOTA demonstrates behaviour that is superior to ^89^Zr–DFO. To the best of our knowledge, this is the first report to evaluate tetraazamacrocycles as ^89^Zr-chelators and to provide a rationale for their exceptional and unpredicted *in vivo* behaviour.

## Experimental methods

Full experimental details are presented in the ESI.‡

## Results and discussion

During initial syntheses of the nonradioactive complexes, we observed poor reactivity when the respective ligands were reacted with zirconium oxalate (Zr(ox)_2_), which demonstrates poor solubility in a variety of solvents. Thus, we used either Zr(iv) acetylacetonate (Zr(AcAc)_4_) or ZrCl_4_ as zirconium sources with subsequent synthetic strategies modified from the literature.^11,16,27^ Accordingly, nonradioactive Zr–DOTA (Scheme S1‡), Zr–DOTP (Scheme S2‡) and Zr–DOTAM (Scheme S3‡) were prepared in excellent yields and all were fully characterized by HPLC, NMR spectroscopy, and HR-MS analyses (Fig. S1–S20‡). While single crystals of all complexes were obtained, only those of Zr–DOTA were suitable for single crystal X-ray diffraction analysis.

**Fig. 2:**
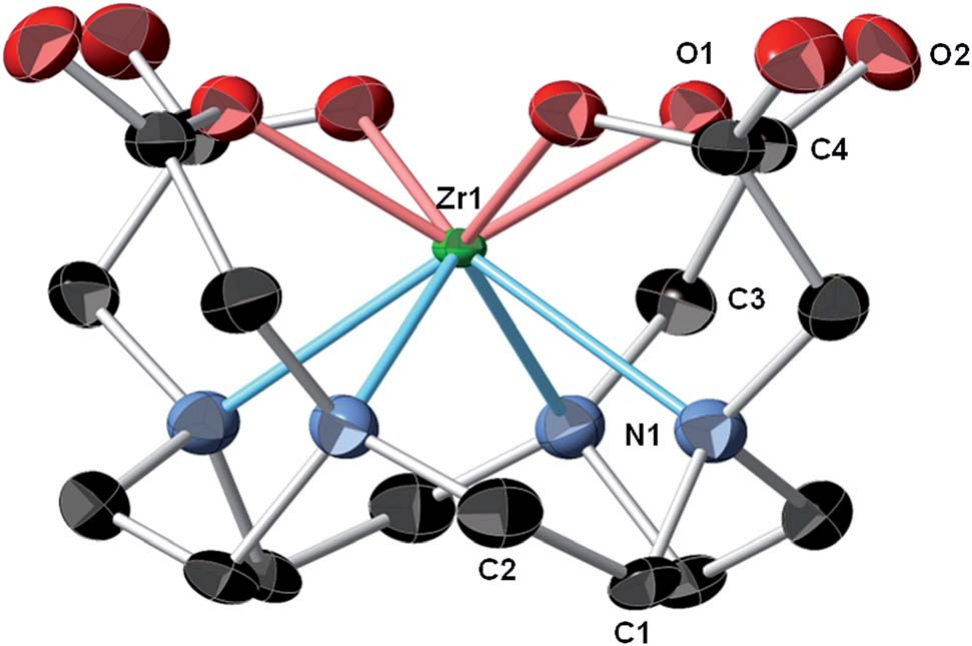
Crystal structure of Zr–DOTA.‡ Thermal ellipsoids are drawn at the 50% probability level; only one of the crystallographically-independent Zr centers is shown and a partial atomic labeling scheme is provided. The disordered Zr center, disordered solvent molecules, and hydrogen atoms are omitted for clarity.

In our hands, single crystal X-ray diffraction of Zr–DOTA revealed 2 crystallographically-independent molecules in the asymmetric unit with *C*_4_ symmetry. One of the Zr sites is disordered (68%/32%) over 2 sites along the 4-fold axis. The ordered site at Zr_1_ is depicted in Fig. 2 and complete crystallographic parameters, data collection and refinement information are included in the ESI.‡ All four macrocycle nitrogen atoms and acetate pendant arms participate in Zr^4+^ ion coordination to form an octa-coordinate complex, which may be the key to the relationship between the complex's structure and its unanticipated *in vivo* behaviour (*vide infra*). The Zr–DOTA complex exhibits a compressed, square anti-prismatic geometry. This is not unusual, since Zr tetraazamacrocycle complexes exhibit varying geometries dictated by the additional ligands that occupy the coordination sites not occupied by the nitrogen atoms of the macrocycle.^25,26,28^ The perpendicular distance from the metal center to the plane described by the 4 acetate-containing pendant arms of the macrocycle is 1.004(3) Å, and the perpendicular distance from the metal center to the plane described by the 4 nitrogens of the macrocycle is 1.310(4) Å. The DOTA ligand displays a low-symmetry saddle-like conformation similar to that of metal–dibenzotetramethytetraaza[14]annulene complexes described by De Angelis and co-workers.^29^ This conformation is most pronounced with metal complexes demonstrating d^0^ electron configurations, which lack crystal-field stabilization.^29^ The average Zr–ligand bond lengths and bond angles are comparable to those observed in structurally characterized Zr complexes containing hydroxamate, phenoxyamine, salophen, or cyclam ligands.^25,26,28,30–33^

After completing synthesis and characterization of the reference complexes, we attempted to radiolabel each ligand using ^89^Zr(ox)_2_ and procedures established for preparation of ^89^Zr–DFO.^5^ However, radiochemical yields were poor (Scheme S4 and Table S6‡). Thus, we used ^89^ZrCl_4_ as a radioactive precursor,^5^ and observed that DOTA, DOTAM, and DOTP were quantitatively radiolabeled within 45 minutes at 90 °C (Scheme S5‡). Optimized radiochemical synthesis conditions are presented in Table S7.‡ The radiochemical yield and purity of all ^89^Zr complexes were confirmed by radio-TLC (Fig. S25, S27 and S29‡) or radio-HPLC (Fig. S26 and S28‡). The specific activity (*A*_s_) for each radiometal complex is in good agreement with the *A*_s_ of other ^89^Zr-complexes reported in the literature.^9,15^ The surprising differences in reactivity observed for ^89^Zr(ox)_2_ and ^89^ZrCl_4_ with tetraazamacrocycles are most likely dictated by the ^89^Zr species present in solution. Nonradioactive Zr(ox)_2_ is a highly stable complex, even under highly acidic conditions and at very low molar concentrations.^34^ Accordingly, the oxalate anion's ability to form a stable ^89^Zr-complex in aqueous media effectively competes with the tetraazamacrocycle ligand, resulting in reduced ^89^Zr-tetraazamacrocycle complex formation. On the other hand, natural ZrCl_4_ (like other highly charged, oxophilic metal halides) readily undergoes aquation in solution to form hydroxo- and oxo-bridged species.^35^ It is reasonable that ^89^ZrCl_4_ would demonstrate a similar behaviour.^34^ Thus, in the absence of an oxalate ligand, ^89^Zr–tetraazamacrocycle complex formation is favoured over the formation of ^89^ZrOH species when ^89^ZrCl_4_ is added to a buffered solution of the macrocycle.

Since tetraazamacrocycles are considered poor ^89^Zr-chelators,^20,22–24^ we evaluated the *in vitro* stability of ^89^Zr–DOTA, ^89^Zr–DOTAM, and ^89^Zr–DOTP by challenging them with excess EDTA, high concentrations of biologically relevant metal ions, or human serum proteins. ^89^Zr–DOTA did not undergo transchelation, with a 100-, 500-, and 1000-fold excess of EDTA at pH 5 or pH 7 over 7 days. In contrast, ^89^Zr–DFO completely lost metal ions after 3 h incubation, with a 1000-fold excess of EDTA at pH 5. Based upon our EDTA challenge studies, the order of ^89^Zr-complex stability can be described as ^89^Zr–DOTA ≫ ^89^Zr–DOTP > ^89^Zr–DOTAM > ^89^Zr–DFO (Table S8‡).

Tetraazamacrocycles can chelate numerous, biologically relevant metal cations, and this property can potentially create a second mechanism for ^89^Zr^4+^ ion dissociation from its chelator *in vivo*.^1^ To assess the ability of the Zr complexes to resist demetallation by another metal cation, we performed metal competition studies, in which we mixed the radiometal complex with an excess concentration of metal salts in aqueous buffer. We observed no demetallation of ^89^Zr–DOTA over the 7 day experiment (Table S9‡). In contrast, ^89^Zr–DFO remained only 33.9% and 72.6% intact, respectively, when challenged with Fe^3+^ ions or Ga^3+^ ions. The overall order of ^89^Zr-complex stability based upon these studies mirrored our results in the EDTA challenge experiments, further demonstrating the robust stability of Zr–tetraazamacrocycle complexes.

We then evaluated the *in vivo* behaviour of ^89^Zr–DOTA, ^89^Zr–DOTP, and ^89^Zr–DOTAM in acute biodistribution studies. Results are shown in Tables S12–S14.‡ Mice receiving ^89^Zr–DOTAM retained elevated levels of radioactivity in liver and spleen tissues, which was not excreted over the 72 h experiment. *In vitro*, ^89^Zr–DOTAM aggregated and precipitated out of solution unless a low concentration of surfactant was included to stabilize the complex. While surfactant was used in the injection formulation for biodistribution studies, it is hypothesized that once in the blood stream, ^89^Zr–DOTAM aggregates with serum proteins, which are deposited in these tissues during circulation. We could not identify the radioactive species, and thus we did not evaluate ^89^Zr–DOTAM further.

Mice intravenously injected with ^89^Zr–DOTA retained significantly less radioactivity in their liver, kidney, and bone tissue compared to mice injected with ^89^Zr–DOTP at 72 h post-injection (^89^Zr–DOTA *vs.* ^89^Zr–DOTP: % ID per g ± SD, *p* value) (blood, 0.0003 ± 0.001 *vs.* 0.0005 ± 0.001, 0.39; liver, 0.021 ± 0.002 *vs.* 0.036 ± 0.002, <0.0001; kidney, 0.078 ± 0.009 *vs.* 0.32 ± 0.045, <0.0001; bone, 0.025 ± 0.009 *vs.* 2.63 ± 0.12, <0.0001). Higher retention of ^89^Zr–DOTP was predicted by the *in vitro* kinetic stability results, and may suggest transchelation to serum proteins or reduced stability in the presence of the lower pH environments that may exist in Kupffer cell lysosomes or the kidney.^36,37^ Retention of radioactivity in bones of mice receiving ^89^Zr–DOTP may be caused by a number of factors, *e.g.* residualization of ^89^Zr transchelated by hydroxylapatite, or adsorption of the intact complex in the bone matrix due to the influence of the four phosphate-containing pendant arms of the ^89^Zr–DOTP complex. The latter phenomenon was observed with other radiometal–DOTP complexes.^1^ Fewer phosphate-containing pendant arms may reduce bone retention, and studies of phosphate-containing ^64^Cu–tetraazamacrocycle complexes show that this strategy reduces the amount of radioactivity retained in bone tissue.^38^

We then compared the performance of ^89^Zr–DOTA and ^89^Zr–DFO (Fig. S37‡). Each had similar blood excretion profiles, but animals injected with ^89^Zr–DOTA had lower radioactivity retention in liver, kidney, and bone tissue at 72 h post-injection (^89^Zr–DOTA *vs.* ^89^Zr–DFO: % ID per g ± SD, *p* value) (blood, 0.0003 ± 0.0008 *vs.* 0.0003 ± 0.0005, 1.00; liver, 0.021 ± 0.002 *vs.* 0.066 ± 0.009, <0.0001; kidney, 0.078 ± 0.009 *vs.* 0.69 ± 0.098, <0.0001; bone, 0.025 ± 0.009 *vs.* 0.079 ± 0.014, <0.0001). Interestingly, while radioactivity retention in bone tissue of mice injected with ^89^Zr–DFO increases over time, radioactivity retention in bone tissue of mice receiving ^89^Zr–DOTA remained low, with no statistically significant changes at any time point. One possible explanation for these observations may be the tetraazamacrocycle's ability to form an octa-coordinate complex with the ^89^Zr^4+^ ion. The saturated coordination sphere plus the four hard oxygen donor groups are believed to produce a complex that remains resistant to chemical, biological, and physical factors that may destabilize a radiometal complex *in vivo*. However, it is unknown if resistance to these forces will be maintained when ^89^Zr–DOTA is incorporated into an antibody conjugate. These studies are currently underway in our laboratory.

**Fig. 3:**
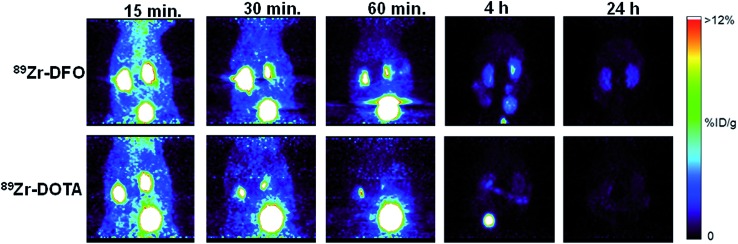
PET maximum-intensity projection images comparing ^89^Zr–DOTA and ^89^Zr–DFO. ^89^Zr–DOTA undergoes more efficient renal excretion than ^89^Zr–DFO. By 4 h post-injection, most activity associated with ^89^Zr–DOTA was excreted into the bladder; by 24 h post-injection, very little activity remained. Mice injected with ^89^Zr–DFO retained significantly more radioactivity in their kidneys at 4 h and 24 h post-injection.

Normal mice were injected with ^89^Zr–DFO and ^89^Zr–DOTA and dynamic PET imaging done from 0–60 minutes, followed by static imaging at 2, 4, and 24 h after injection. Both radiometal complexes exhibited a similar excretion profile based on the amount of radioactivity in the blood pool and the liver during first 60 minutes (Fig. 3, S38, and S39‡). Radioactivity in the kidney and bone was much lower in mice receiving ^89^Zr–DOTA compared to ^89^Zr–DFO, suggesting a better excretion profile from these tissues (see Fig. 3 and S38‡). Both ^89^Zr–DFO and ^89^Zr–DOTA are excreted renally, with elevated levels of radioactivity in the kidneys and bladder at early time points. However, by 4 h, nearly all the radioactivity was excreted from mice that received ^89^Zr–DOTA, and after 24 h, radioactivity was barely above background levels. By contrast, more radioactivity accumulated in the kidneys of mice injected with ^89^Zr–DFO at 4 h, and they were still visible in static images acquired after 24 h. Results of region-of-interest analysis on the data acquired during the static imaging sessions further corroborate our biodistribution studies, which suggest that the *in vivo* behavior of ^89^Zr–DOTA is superior to ^89^Zr–DFO (Table S16‡). However, only metabolism studies will provide definitive proof of the radioactive species retained or excreted. Accordingly, we are currently performing such studies to examine the fates of ^89^Zr–oxalate, ^89^ZrCl_4_, ^89^Zr–DFO, and ^89^Zr–DOTA in mouse tissues.

Based on these unexpected observations, we attempted to place our results in context by comparing them with published studies of other ^89^Zr-chelators (Tables S17–S19‡). Variability in study designs or unreported data prevented direct comparisons among all ligand classes, but comparisons were made when possible.

*In vitro* data suggest that ^89^Zr–DOTA was more resistant to EDTA challenge than ^89^Zr–TAFC,^39^ which was 97% intact after 7 days exposure to 1000 fold EDTA (pH 7). Additionally, metal ion competition studies suggest that ^89^Zr–DOTA was more resistant to exogenous metal challenge than either ^89^Zr–HOPO or ^89^Zr–CP256.^9,14^ When exposed to Fe^3+^ ions, only 83% of the ^89^Zr–HOPO complex was still intact after 7 days, and only 14% of ^89^Zr–CP256 remained intact after 20 minutes. Finally, *in vivo* biodistribution data were also examined at 24 h post-injection. Mice injected with ^89^Zr-TAM-1, ^89^Zr-TAM-2, and ^89^Zr-2,3-HOPO retained 26, 145, and 92 fold more radioactivity, respectively, in kidney tissue than mice injected with ^89^Zr–DOTA.^15,19^ In the same ligand series, mice injected with the corresponding radiometal chelates retained 2.6, 7.6 and 7.6 fold more radioactivity, respectively in bone tissue than did animals receiving ^89^Zr–DOTA.^15,19^ Also, approximately 5 and 16 fold more radioactivity was observed in the bone tissue of animals injected with ^89^Zr–HOPO and ^89^Zr–L4, respectively.^9,10^ This limited comparison of *in vitro* and *in vivo* behaviour suggests that ^89^Zr–DOTA is the most stable ^89^Zr-complex reported to date, and its apparent resilience to perturbation *in vitro* and *in vivo* is remarkable and unexpected.

Although the elevated temperature needed to synthesize ^89^Zr tetraazamacrocycle complexes may be considered a limitation of this work, it should not prohibit exploration of these radiometal chelates in immuno-PET applications, since various methods can be used to circumvent this temperature requirement and prepare useful ^89^Zr-radiopharmaceuticals.^40–42^ Furthermore, the use of ^89^ZrCl_4_ as a ^89^Zr-source allows access to ultra-stable ^89^Zr-complexes, previously believed to be inaccessible or unstable. The synthetic methodologies we describe here can facilitate systematic study of ^89^Zr coordination chemistry using inorganic chemistry, radiochemistry and molecular imaging techniques to elucidate how to create ^89^Zr-radiopharmaceuticals with excellent stability *in vivo*. While many ligands have been developed to chelate ^89^Zr, a systematic study among ligand classes has not been described in the literature. Just as a systematic study of tetraazamacrocycles benefited ^64^Cu radiopharmaceutical development and led to the ultra-stable cross-bridged chelators,^43–46^ similar advances could be accomplished based on systematic study of these ^89^Zr-tetraazamacrocycle complexes. Finally, clinicians are increasingly using ^89^Zr–DFO–mAbs in dosimetry and therapeutic planning before targeted systemic radiotherapy.^47–51^ Since DOTA can effectively chelate ^89^Zr and other therapeutic radionuclides,^52–54^ it is plausible to imagine that one DOTA–mAb conjugate would be needed to accomplish dosimetry and radiotherapy with ^89^Zr and a therapeutic radionuclide, respectively. This approach may increase dosimetric accuracy, reduce regulatory burden, and minimize costs associated with cGMP-compliant radiopharmaceutical development, so that these precision medicine applications may be used more effectively in the future.

## Conclusions

This report is the first to describe the structural characterization of Zr–DOTA using single-crystal X-ray diffraction and the use of tetraazamacrocycles as ^89^Zr-chelators. In all studies, ^89^Zr–DOTA demonstrated superior *in vivo* behaviour compared to ^89^Zr–DFO, which is considered the “gold standard” in clinical ^89^Zr-radiopharmaceutical development. These results refute current thinking regarding the use of tetraazamacrocycles as ^89^Zr-chelators, and may provide a way to enhance development of radiolabeled agents for precision medicine applications.
