# Supplementary material for: Zirconium tetraazamacrocycle complexes display extraordinary stability and provide a new strategy for zirconium-89-based radiopharmaceutical development
Source: Chem Sci. 2016 Dec 13;8(3):2309–14. doi: 10.1039/c6sc04128k (PMC5363373; doi:10.1039/c6sc04128k)
Supplement: Supplementary file 1 [file SC-008-C6SC04128K-s001.pdf]

Electronic Supplementary Material (ESI) for Chemical Science.
This journal is © The Royal Society of Chemistry 2016

Zirconium Tetraazamacrocycle Complexes Display Extraordinary Stability and Provide a New Strategy for Zirconium-89-Based Radiopharmaceutical Development

Darpan N. Pandya[a]*, Nikunj Bhatt[a], Hong Yuan[b], Cynthia S. Day[c], Brandie M. Ehrmann[d], Marcus Wright[c], Ulrich Bierbach[c], and Thaddeus J. Wadas[a]*

a) Department of Cancer Biology, Wake Forest School of Medicine, Winston-Salem, NC 27157 USA,
b) Department of Radiology, University of North Carolina at Chapel Hill, Chapel Hill, NC 27599 USA,
c) Department of Chemistry, Wake Forest University, Winston-Salem, NC 27109 USA.
d) Department of Chemistry, University of North Carolina at Chapel Hill, Chapel Hill, NC 27599 USA.

*The corresponding authors for this manuscript are listed below.

Thaddeus J. Wadas, Ph.D.
Assistant Professor of Cancer Biology and Radiology
Wake Forest School of Medicine
Medical Center Blvd.
Winston-Salem, NC 27157
phone: (336) 716-5696
fax: (336) 716-0255
e-mail: twadas@wakehealth.edu

Darpan N. Pandya, Ph.D.
Department of Cancer Biology

Wake Forest School of Medicine
Medical Center Blvd.
Winston-Salem, NC 27157
phone: (336) 713-7164
fax: (336) 716-0255
e-mail: dapandya@wakehealth.edu

Table of Contents

Section	Page Number
Abbreviations	3
Materials and methods	3-4
Scheme 1. Synthesis of ^{Na}Zr-DOTA	5
HPLC, NMR, HRMS analysis of ^{Nat}Zr-DOTA (Fig. S1-S9)	6-14
Scheme 2. Synthesis of ^{Na}Zr-DOTP	15
HPLC, NMR, HRMS analysis of ^{Nat}Zr-DOTP (Fig. S10-S16)	16-22
Scheme 3. Synthesis of ^{Na}Zr-DOTAM	23
NMR, HRMS analysis of ^{Nat}Zr-DOTAM (Fig. S17-S20)	24-27
Crystal Structure analysis of ^{Na}Zr-DOTA (Fig. S21-S23)	28-32
Crystal structure data collection parameters (Table S1-S5)	33-45
Scheme S4. Radiochemical Synthesis of ^{89}Zr-DOTA, ^{89}Zr-DOTP, and ^{89}Zr-DOTAM using $^{89}Zr(ox)_2$	46
Radiochemistry protocol using $^{89}Zr(ox)_2$ for ^{89}Zr-DOTA, ^{89}Zr-DOTP, and ^{89}Zr-DOTAM (Table S6)	47
Preparation of $^{89}ZrCl_4$ from $^{89}Zr(ox)_2$ (Fig. S24)	48
Scheme S5. Radiochemical Synthesis of ^{89}Zr-DOTA, ^{89}Zr-DOTP, and ^{89}Zr-DOTAM using $^{89}ZrCl_4$	49
Radiochemistry protocol using $^{89}ZrCl_4$ for ^{89}Zr-DOTA	50
Quality control of ^{89}Zr-DOTA by radio-TLC (Fig. S25)	51
Quality control of ^{89}Zr-DOTA by radio-HPLC (Fig. S26)	52
Radiochemistry protocol using $^{89}ZrCl_4$ for ^{89}Zr-DOTP	53
Quality control of ^{89}Zr-DOTP by radio-TLC (Fig. S27)	54
Quality control of ^{89}Zr-DOTP by radio-HPLC (Fig. S28)	55
Radiochemistry protocol using $^{89}ZrCl_4$ for ^{89}Zr-DOTAM	56
Quality control of ^{89}Zr-DOTAM by radio-TLC (Fig. S29)	56
Summary of optimized radiochemistry conditions to prepare ^{89}Zr-complexes with $^{89}ZrCl_4$ (Table S7)	57
In vitro EDTA challenge study (Table S8)	58-60
In vitro metal competition study (Table S9)	61-62
In vitro serum stability study (Fig. S30-S36)	63-67
Summary of *In Vitro* serum stability of ^{89}Zr-complexes (Table S10)	68
Determination of partition coefficients (logP) (Table S11)	69
Complete Biodistribution studies of ^{89}Zr-DOTA (Table S12), ^{89}Zr-DOTP (Table S13), ^{89}Zr-DOTAM (Table S14), and ^{89}Zr-DFO (Table S15)	70-74
Summary of biodistribution data of ^{89}Zr-complexes (Fig. S37)	75
Small animal PET/CT imaging protocol of ^{89}Zr-DFO and ^{89}Zr-DOTA	76
Dynamic PET data comparing ^{89}Zr-DFO and ^{89}Zr-DOTA in specific tissues (Fig. S38)	77
Small animal PET/CT imaging of ^{89}Zr-DFO and ^{89}Zr-DOTA at 4 h post-injection (Fig. S39)	78
Image-derived biodistribution of ^{89}Zr-DFO and ^{89}Zr-DOTA (Table S16)	79
Tables comparing *in vitro* and *in vivo* properties of ^{89}Zr-DOTA with ^{89}Zr-chelates reported in the literature (Tables S17, S18 and S19)	80-82
References	83

Abbreviations Used:

DFO: Deferoxamine; DOTA: 1,4,7,10-tetraazacyclododecane-1,4,7,10-tetraacetic acid; DOTP: 1,4,7,10-tetraaza cyclododecane-1,4,7,10-tetra(methylene phosphonic acid); DOTAM: 1,4,7,10-tetrakis(carbamoylmethyl)-1,4,7,10-tetraazacyclododecane; EDTA: Ethylenediaminetetraacetic acid; $Zr(AcAc)_4$: Zirconium(IV) acetylacetonate; $Zr\text{-}(ox)_2$: Zirconium(IV) Oxalate; TFA: Trifluoroacetic acid; MeOH: Methanol; HEPES: 4-(2-Hydroxyethyl)piperazine-1-ethanesulfonic acid; NMR: Nuclear magnetic resonance; UV-HPLC: Ultra-Violate High performance liquid chromatography; RT: retention time; R_f: retention factor; ESI-HR-MS: Electrospray Ionization High Resolution Mass Spectrometry; HS, human serum; ITLC: Instant Thin Layer Chromatography; kBq: Kilobecquerel; MBq: Megabecquerel; %ID/g: Percent Injected Dose Per Gram of Tissue; P.I.: Post-injection; ROI: Region of Interest; CPM: counts per minute; TLC: Thin Layer Chromatography; PET: Positron emission tomography; CT: Computed tomography

Materials and Methods

Zirconium-89 (^{89}Zr: ($t_{½}$ = 78.4 h, β^+: 22.8 %, $E_{\beta+max}$ = 901 keV; EC: 77%, E_γ = 909 keV) was purchased from Washington University School of Medicine (St. Louis, MO) or Zevacor, Inc. (Dulles, VA). Unless otherwise noted, all other chemicals were purchased from Sigma-Aldrich Chemical Co. (St. Louis, MO), and solutions were prepared using ultrapure water (18 MΩ-cm resistivity). Ligands (DOTA, DOTP, and DOTAM) were purchased from Marcocyclics, Inc. (Dallas, TX). High-resolution mass spectrometry data was acquired using a Thermo LTQ-FT (7 Tesla) system at the University of North Carolina Chapel Hill Mass Spectrometry Core Laboratory. NMR spectra were obtained using a Bruker DRX 500 MHz spectrometer equipped with a 5mm tbi z-axis gradient probe. All data were collected and processed with Topspin 1.3 using standard Bruker processing parameters. 2D ^1H-^1H gsCOSY, gsHMQC and gsHMBC were collected as 2K by 256 point data sets at 25° C and processed to 1K x 512 blocks. 1H (500 MHz) chemical shifts are reported in parts per million (ppm) relative to the solvent resonances, taken as δ 4.79 for D_2O. Solvent suppression (1D 1H NMR) was

carried out with pre-saturation of the HOD signal. 13C NMR (126 MHz) chemical shifts were referenced externally to TSP (0.00 ppm)

Radiochemistry reaction progress and purity were analyzed by using an analytical HPLC system (Waters, Milford, MA), which runs Empower software and is configured with a 1525 binary pump, 2707 autosampler, 2998 photodiode array detector, 2475 multichannel fluorescence detector, 1500 column heater, fraction collector, HYPERCARB C18 column (5 μ, 4.6 × 100 mm, Thermo Scientific) and a Carrol Ramsey 105-s radioactivity detector (Berkeley, CA). All ligands (DOTA, DOTP, and DOTAM) and associated ^{Nat}Zr-complexes were monitored at 201 nm using a mobile phase consisting of 0.1% TFA/H_2O (solvent A) and 0.1% TFA/acetonitrile (solvent B), and a gradient consisting of 0% B to 70% B in 20 min at a flow rate of 1 mL/min. In addition, radio-TLC was conducted on a Bioscan AR 2000 radio-TLC scanner equipped with a 10% methane:argon gas supply and a PC interface running Winscan v.3 analysis software (Eckert & Ziegler, Berlin, DE). Varian ITLC-SA strips and Merck C-18 TLC plates were employed using a 0.1 M EDTA (pH 5) and 1:1 MeOH:10% NH_4Cl solution as eluents respectively, and ^{89}Zr-Oxalate or $^{89}ZrCl_4$ as a standard control. Radioactive samples were counted using a Perkin Elmer 2480 Wizard® gamma counter (Waltham, MA) with an energy window of 500-1500 keV. PET and CT images were acquired using a GE eXplore Vista small animal PET/CT scanner (Waukesha, WI).

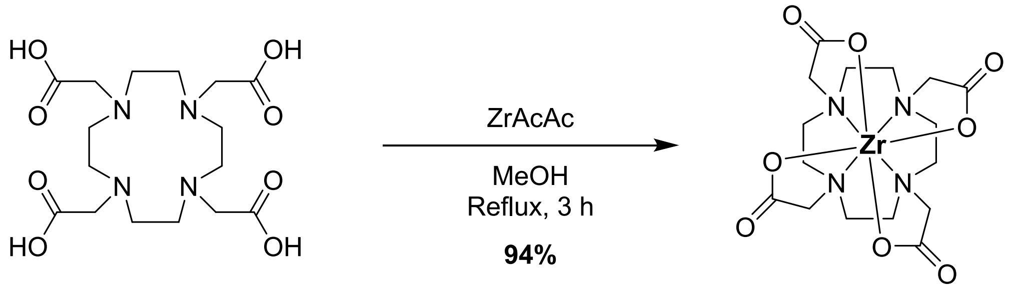

DOTA **Zr-DOTA**

Scheme S1. Synthesis of zirconium complex of DOTA

Synthesis of Zr-DOTA. ZrAcAc (702 mg, 1.44 mmol) was added to a solution of DOTA (529 mg, 1.31 mmol) in 40 mL of methanol. The resulting solution was refluxed for 3 h. As the reaction proceeded, a white precipitate formed. It was filtered, washed with MeOH (2 X 10 mL), and dried in an oven (604 mg, 94% yield). Formation of **Zr-DOTA** complex was confirmed by HPLC (Fig. S1), NMR spectroscopy (Fig. S2 - S7), and HRMS analysis (Fig. S8, S9). ^{1}H NMR (500 MHz, D_2O): δ 3.40 (d, J = 17.6 Hz, 4H), 3.15 (d, J = 17.6 Hz, 4H), 2.99 (td, J = 14.4, 3.8 Hz, 4H), 2.58 (dd, J = 14.5, 3.5 Hz, 4H), 2.38 (td, J = 14.2, 3.5 Hz, 4H), 2.29 (dd, J = 14.7, 3.4 Hz, 4H); ^{13}C NMR (126 MHz, D_2O) δ 178.74, 67.02, 56.41; HRMS (ESI FT-ICR): Calculated for $C_{16}H_{25}N_4O_8Zr$, 491.0714 [$(M+H)^+$] Found: 491.0709 [$(M+H)^+$]. Crystals suitable for X-ray structure determination were grown by dissolving Zr-DOTA in water at 80 °C and allowing the solvent to slowly evaporate at room temperature.

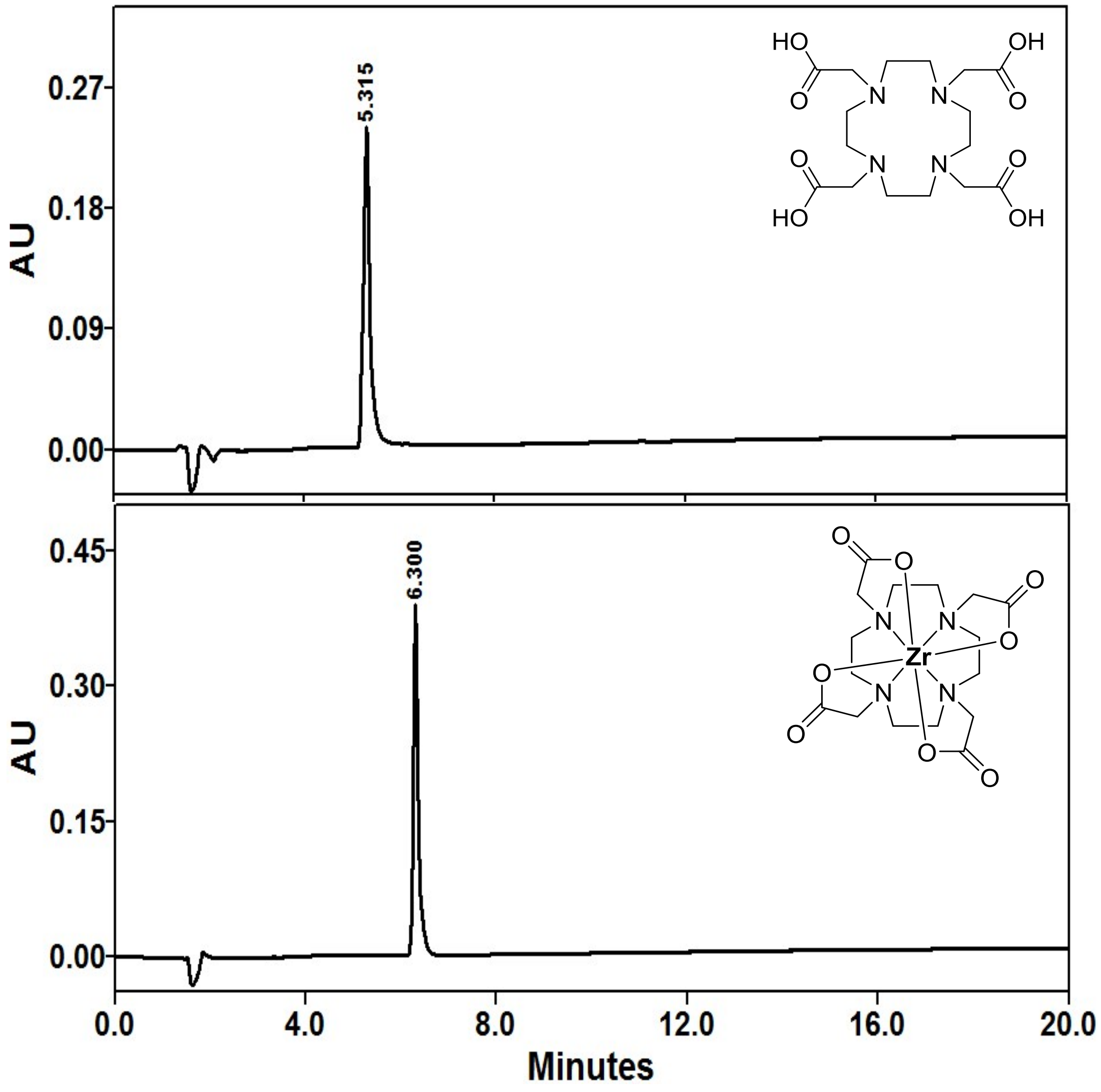

Figure S1. UV-HPLC chromatogram (201nm) of DOTA ligand (top) and nonradioactive ^{Nat}Zr-DOTA complex (bottom)

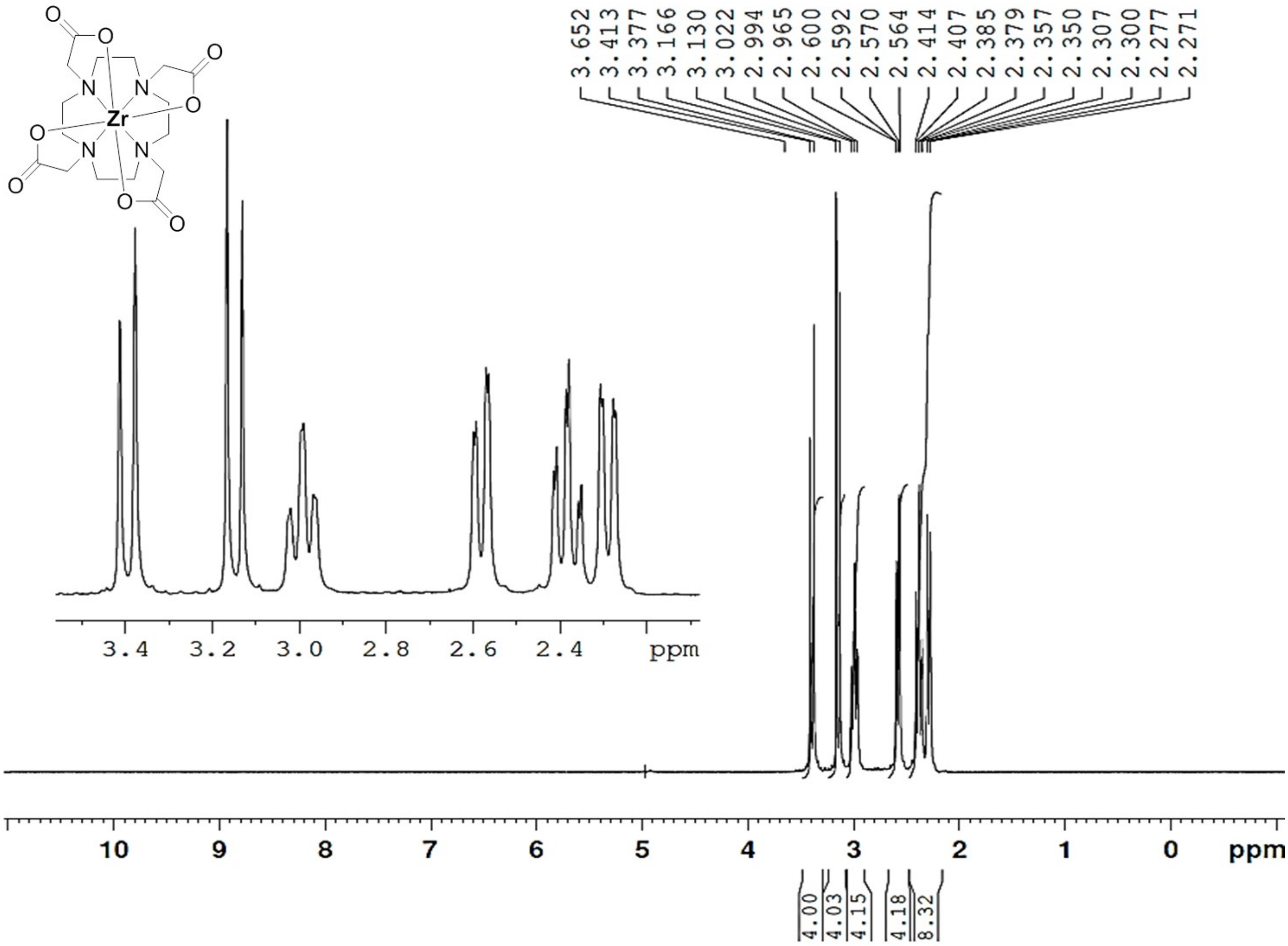

Figure S2. ^{1}H-NMR spectrum (D_2O-500 MHz) of the Zr-DOTA complex

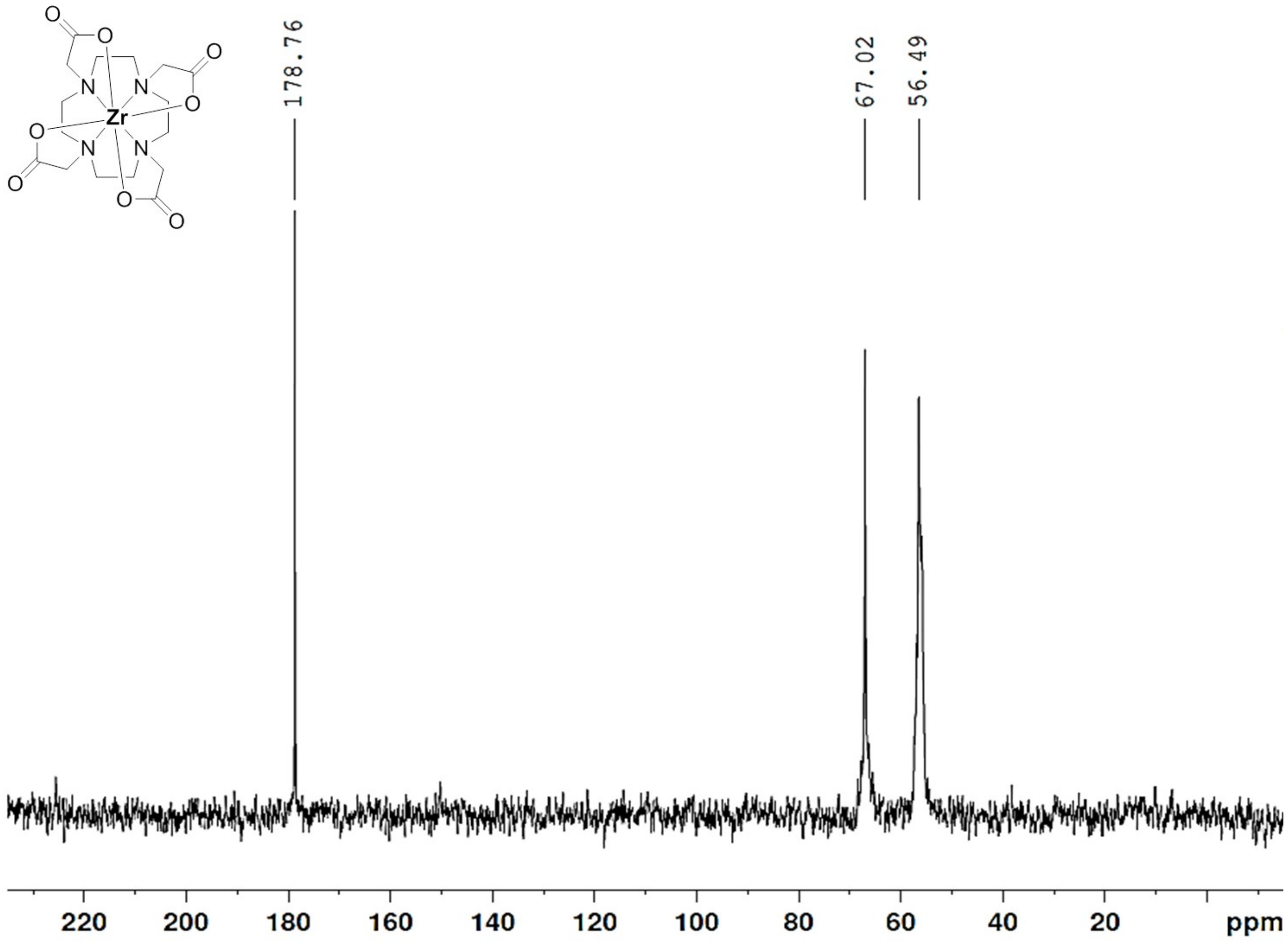

Figure S3. ^{13}C-NMR spectrum (D_2O-126 MHz) of the Zr-DOTA complex

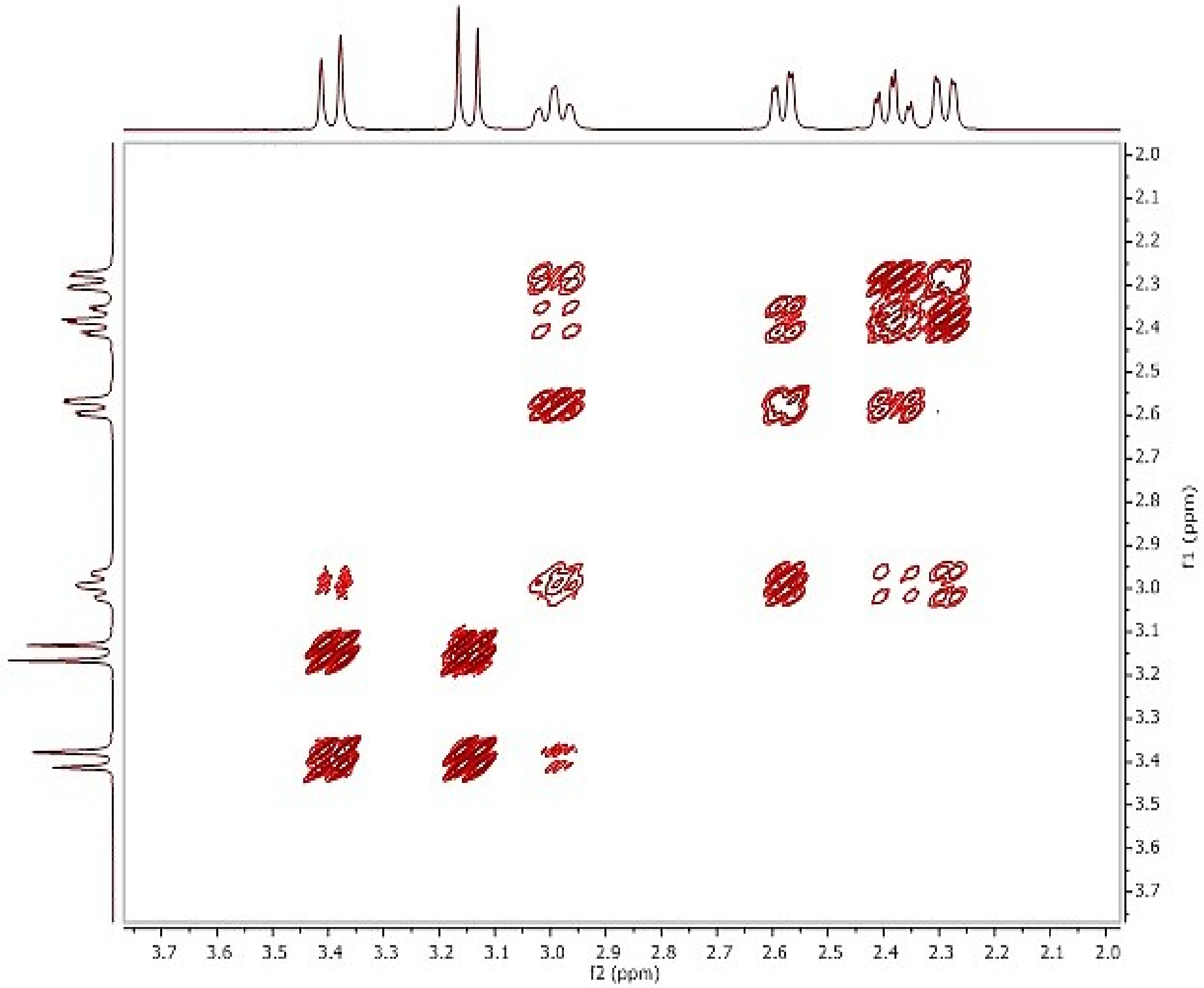

Figure S4. 2-D COSY NMR spectrum (D_2O-500 MHz) of the Zr-DOTA complex

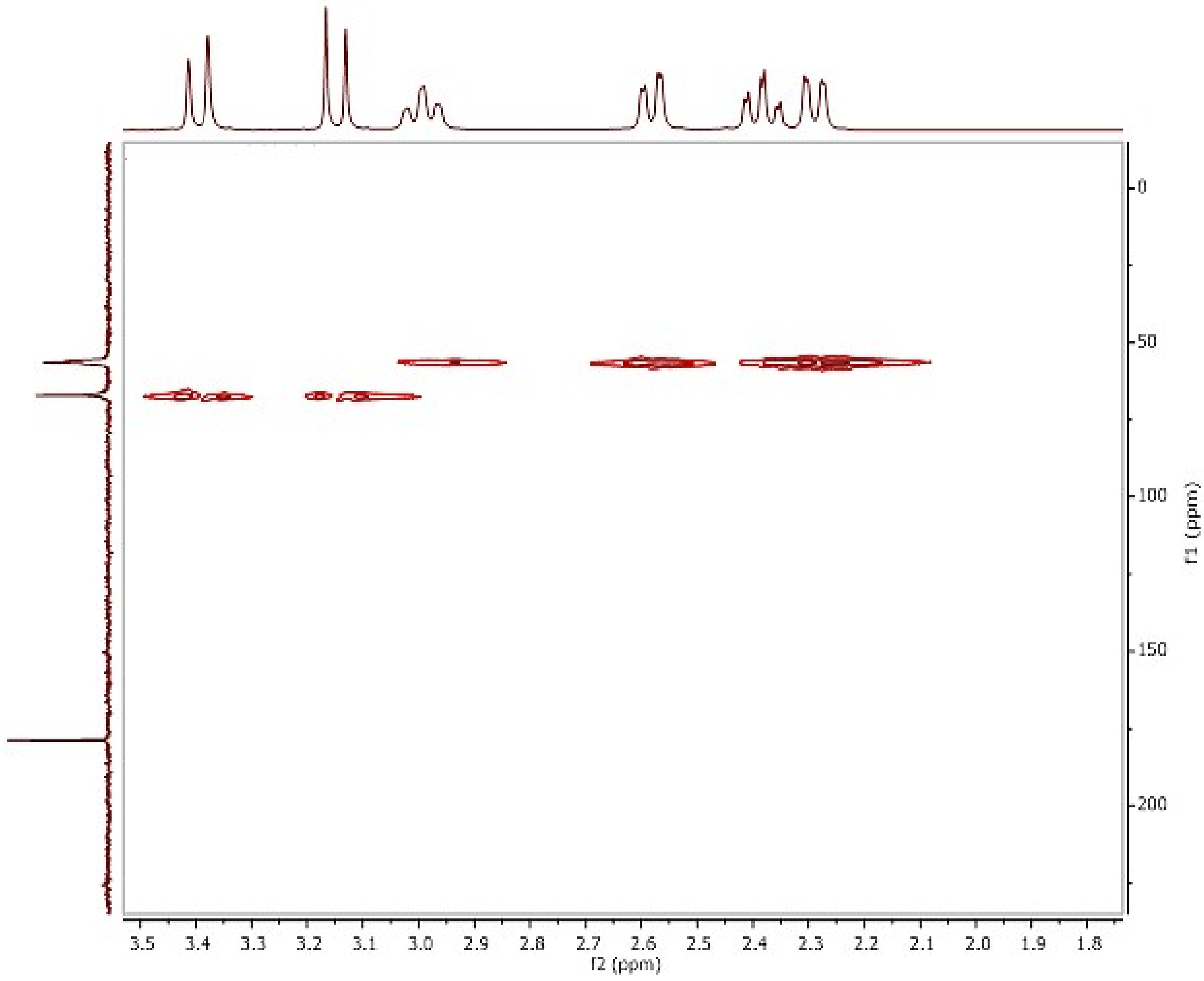

Figure S5. 2-D HMQC NMR spectrum (D_2O-500 MHz) of the Zr-DOTA complex

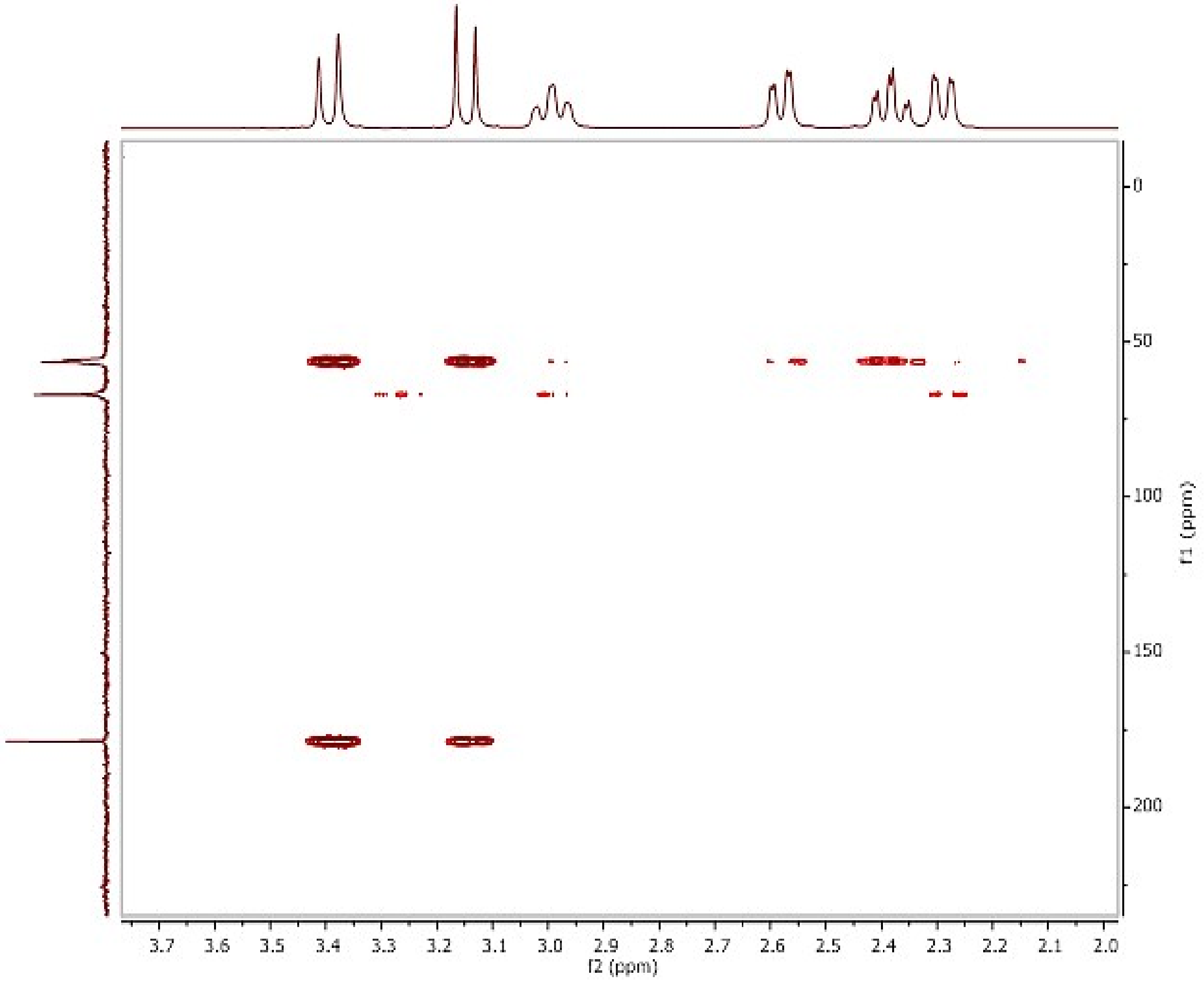

Figure S6. HMBC-2D-NMR spectrum (D_2O-500 MHz) of the Zr-DOTA complex

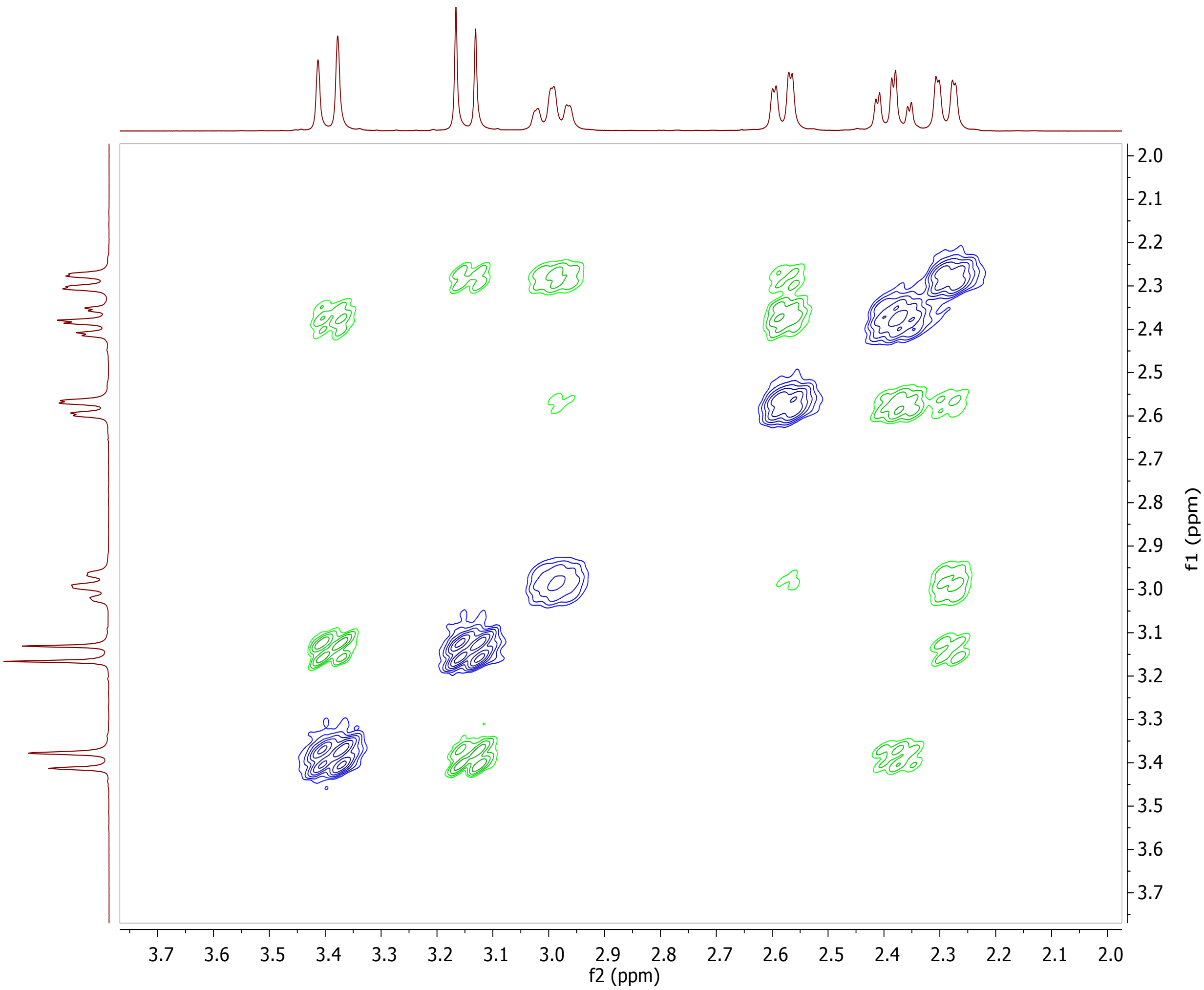

Figure S7. NOESY-2D-NMR spectrum (D_2O-500 MHz) of the Zr-DOTA complex

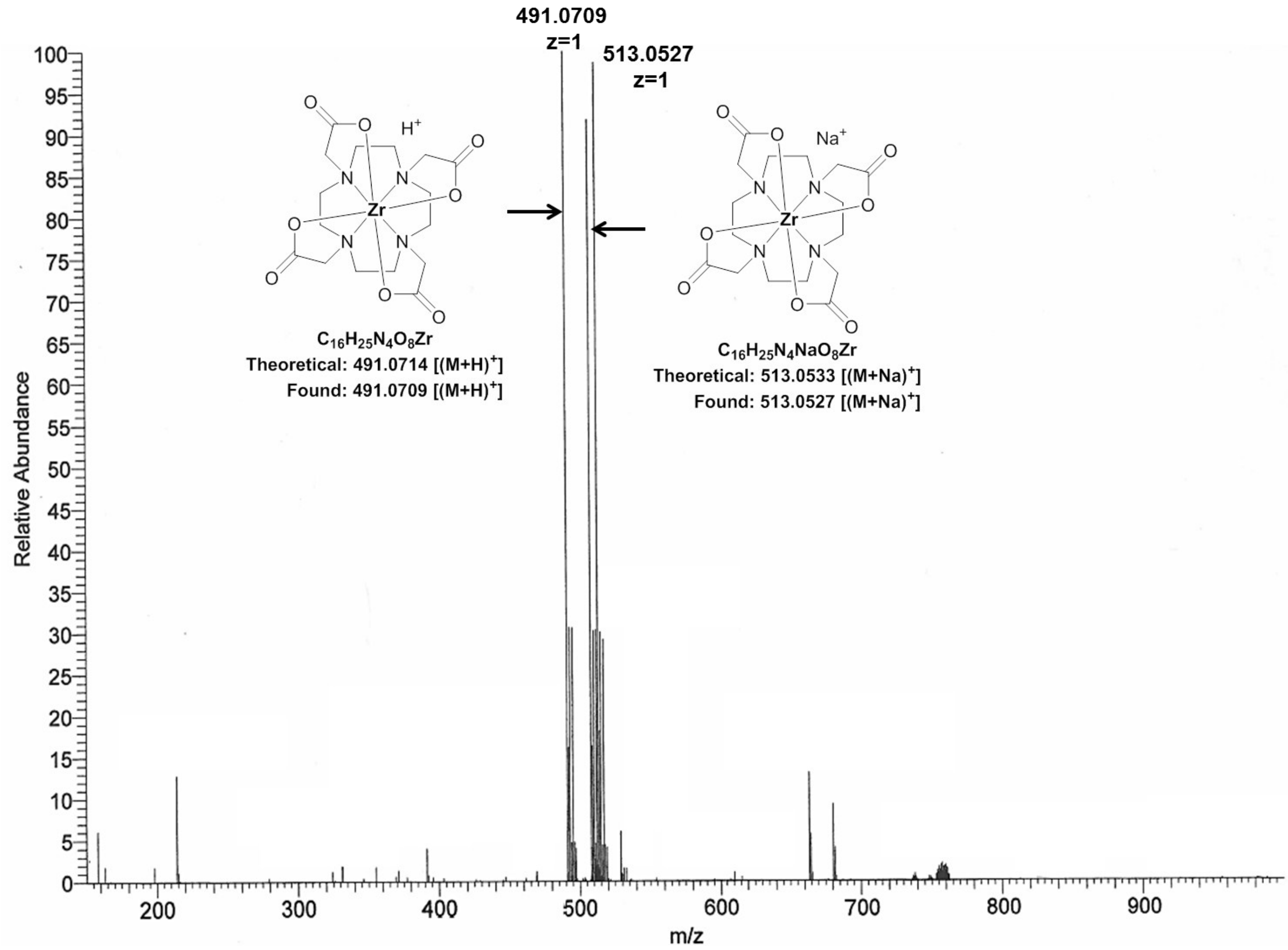

Figure S8. HR ESI FT-ICR MS (Positive mode) analysis of the Zr-DOTA complex

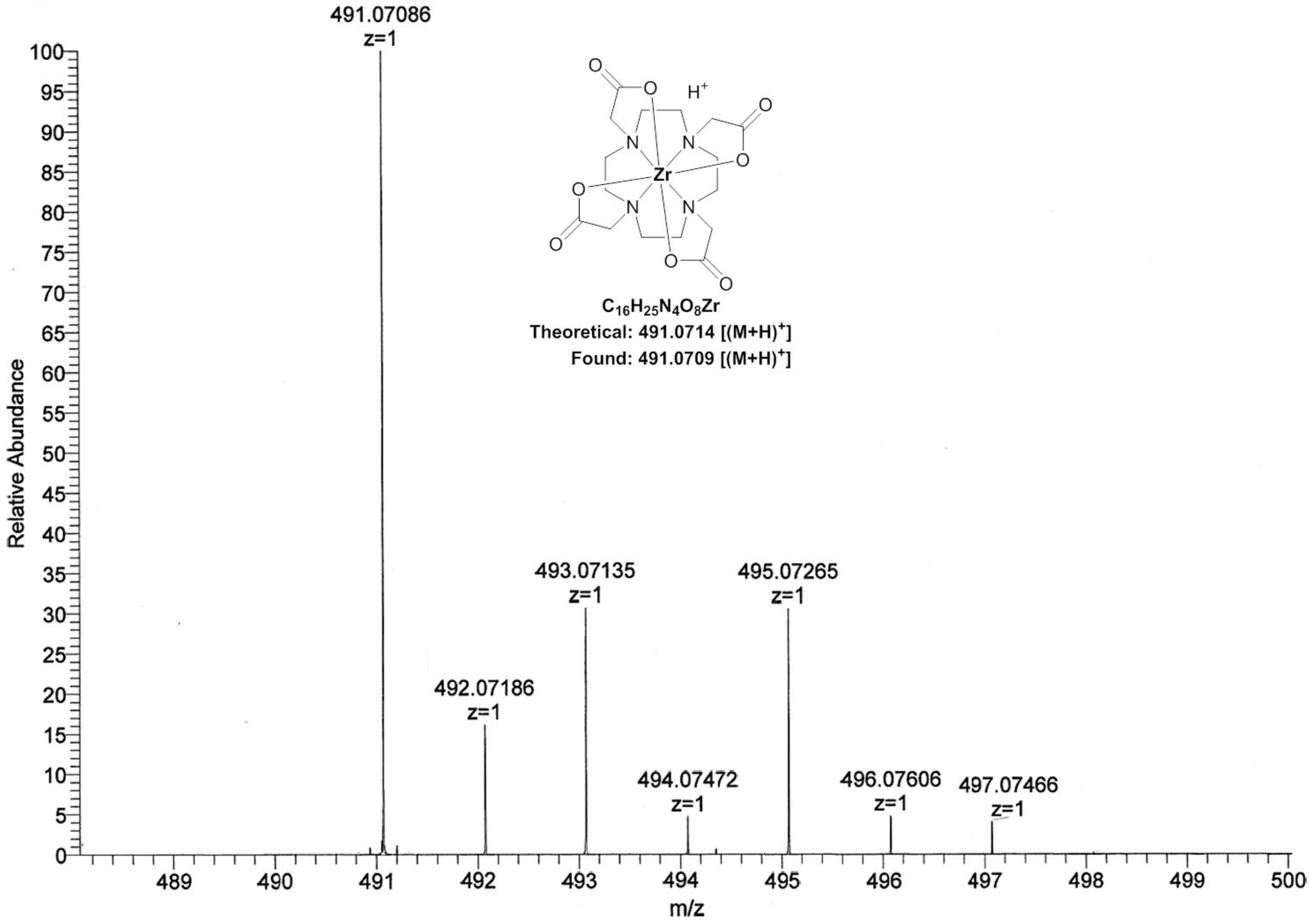

Figure S9. HR ESI FT-ICR MS (Positive mode) analysis of the Zr-DOTA complex

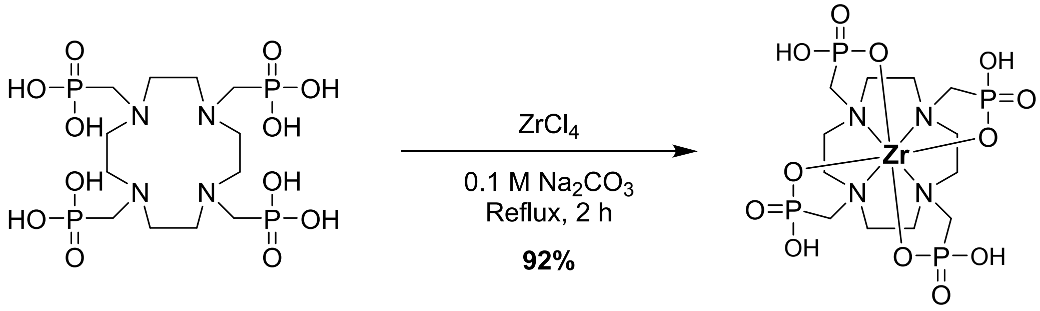

DOTP **Zr-DOTP**

Scheme S2. Synthesis of zirconium complex of DOTP

Synthesis of Zr-DOTP. 0.1 M Na_2CO_3 (2.2 mL) was added to a solution of DOTP (514 mg, 0.94 mmol) and $ZrCl_4$ (240 mg, 1.03 mmol) in 40 mL of water to adjust pH to 7-7.2. The resulting clear solution was refluxed for 2 h, cooled, and filtered through a celite bed. Then filtrate was lyophilized to give a white solid, which was washed with EtOH (2 X 10 mL) and dried in an oven (548 mg, 92% yield). Formation of **Zr-DOTP** complex was confirmed by HPLC (Fig. S10), NMR spectroscopy (Fig. S11 – S14), and HRMS analysis (Fig. S15, S16). ^{1}H NMR (500 MHz, D_2O): δ 4.10 (td, J = 1J and 2J H-H 15 Hz, 4J 31P-1H 4H), 3.72 (dd, J = 2J 31P-1H 16 Hz, 2J 1H-1H 13 Hz, 4H), 3.35 (tt, 1J and 2J H-H 14 Hz, 4J 31P-1H Hz, 4H), 2.53-2.45 (m, 12H); ^{13}C NMR (126 MHz, D_2O) δ 56.65 (d, 1J 31P-13C 138 Hz), 53.67, 52.63 (d, 3J 31P-13C 15 Hz); 31P{1H} NMR (202 MHz, D_2O) δ 33.63; HRMS (ESI FT-ICR): Calculated for $C_{12}H_{27}N_4O_{12}P_4Zr$, 632.9628 [$(M-H)^-$] Found: 632.9636 [$(M-H)^-$].

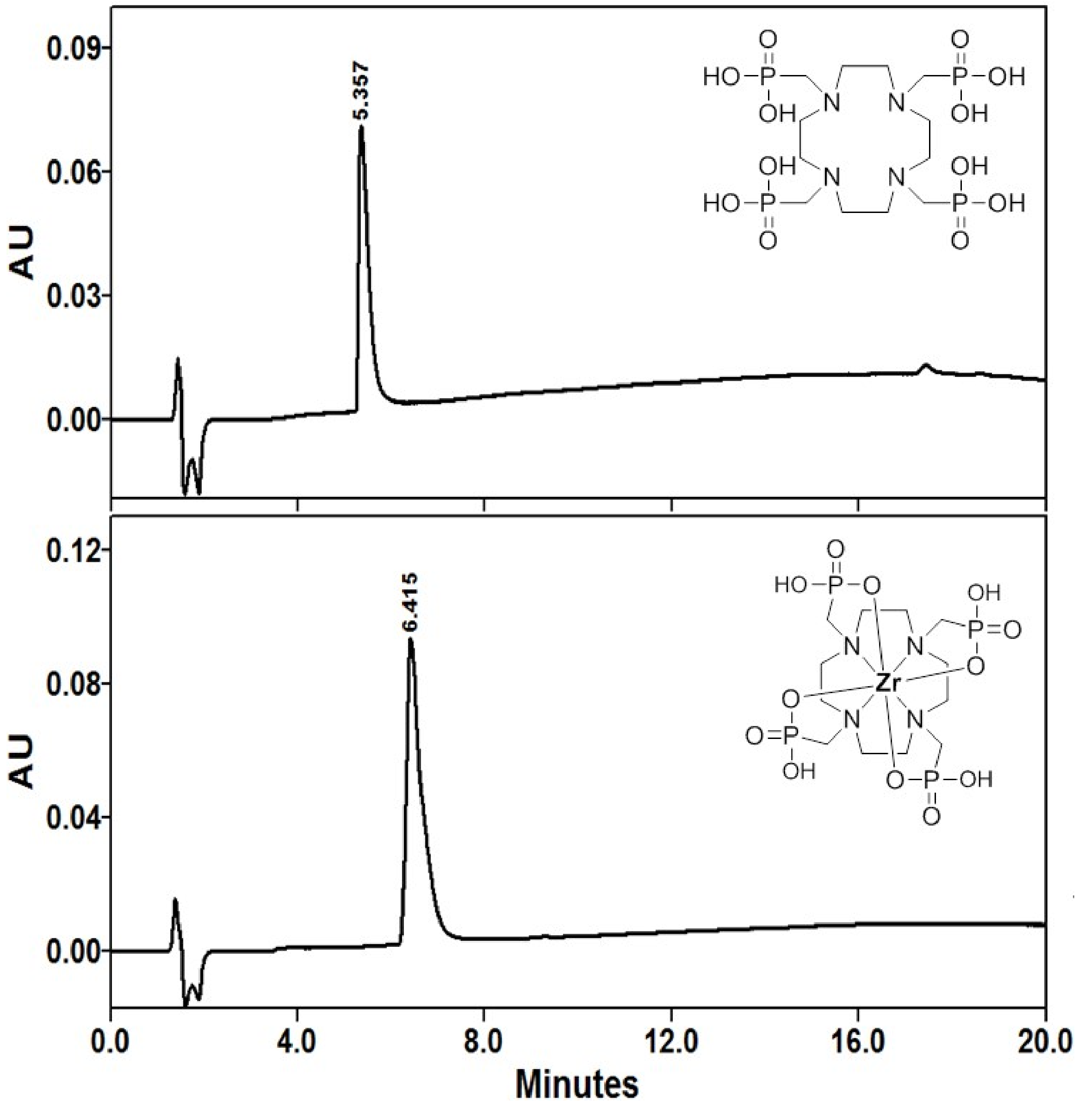

Figure S10. UV-HPLC chromatogram (201 nm) of DOTP ligand (top) and nonradioactive ^{Nat}Zr-DOTP complex (bottom)

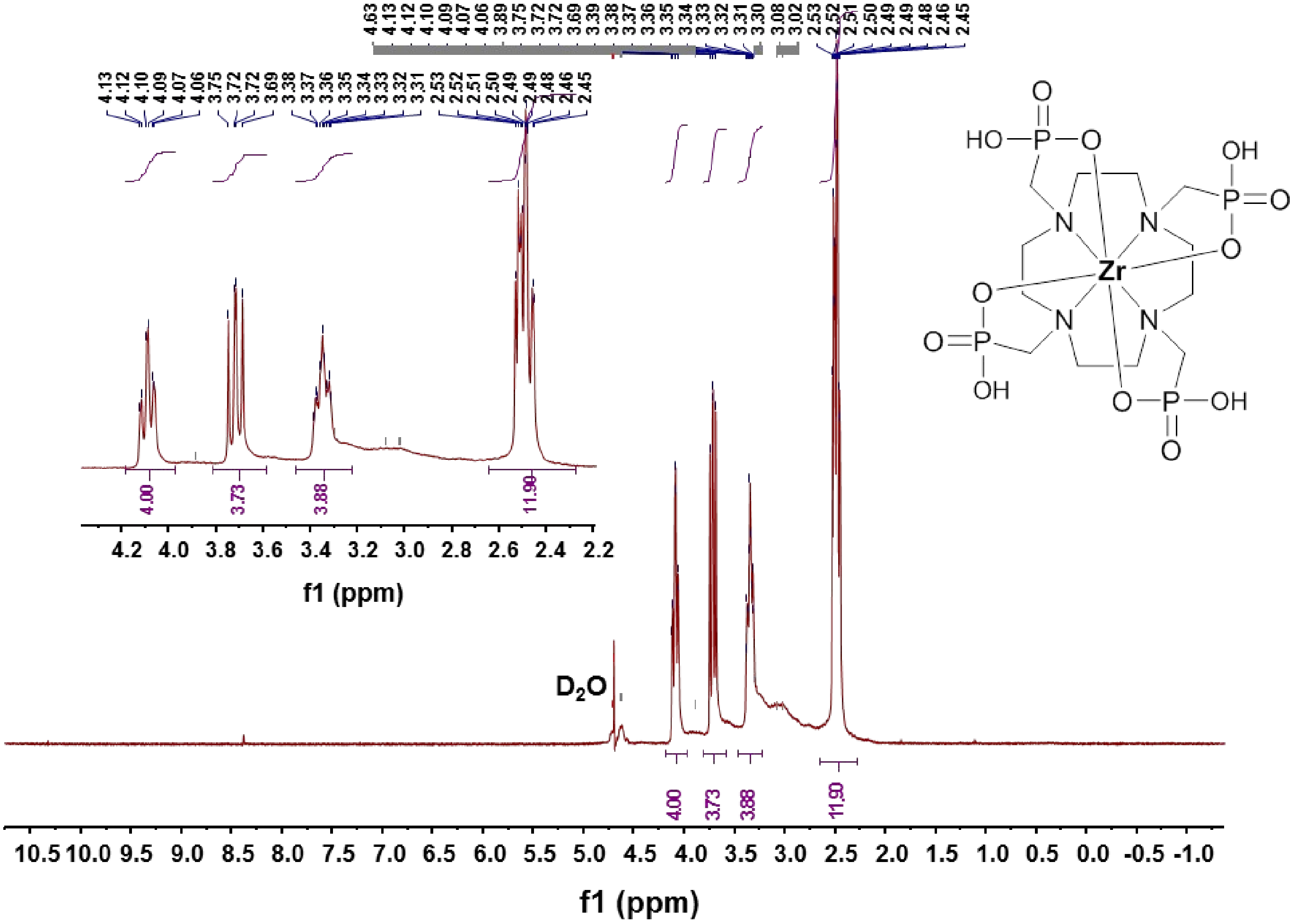

Figure S11. ^{1}H- NMR spectrum (D_2O-500 MHz) of the Zr-DOTP complex

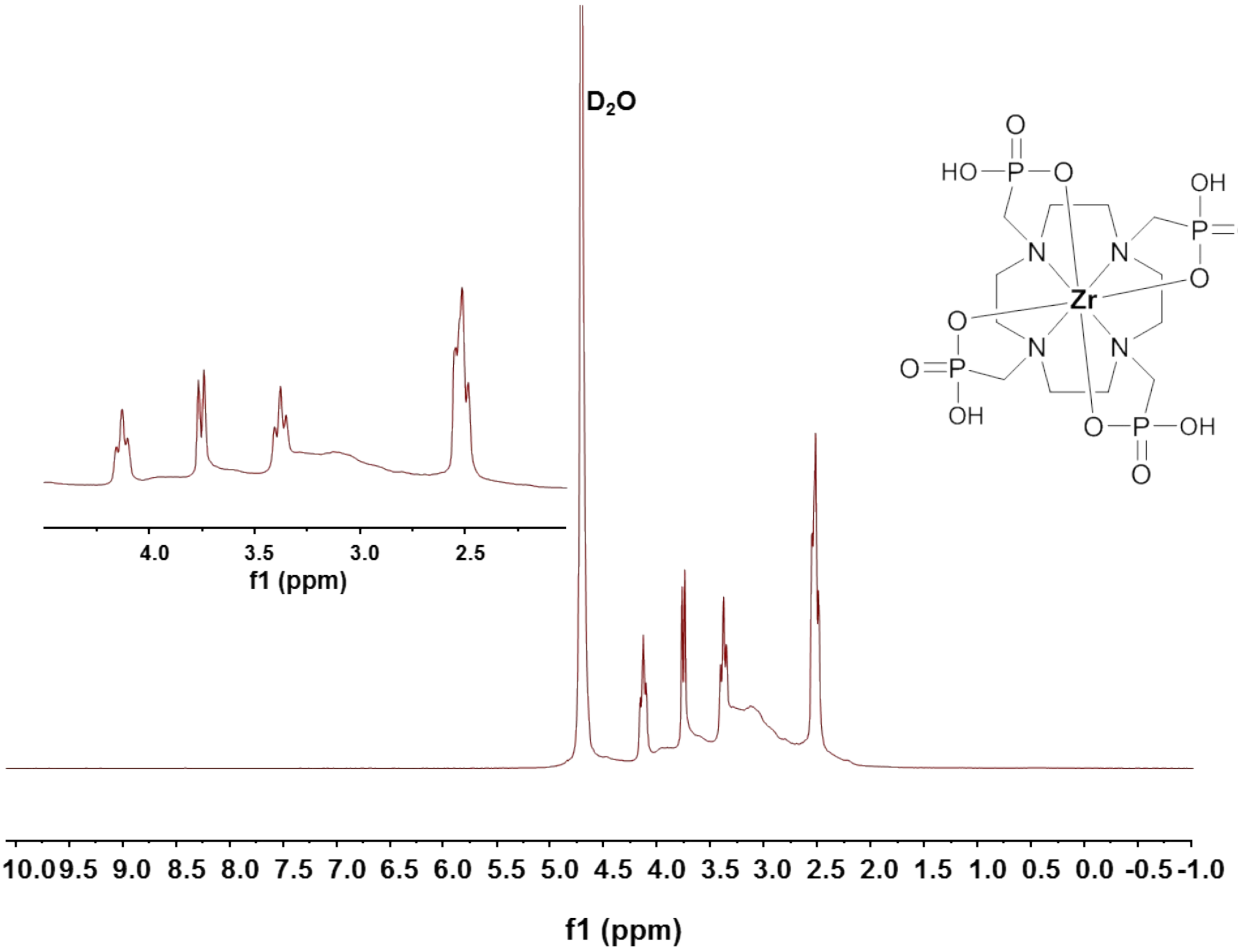

Figure S12. **^{1}H- NMR spectrum (D_2O-500 MHz) with ^{31}P decoupling of the Zr-DOTP complex**

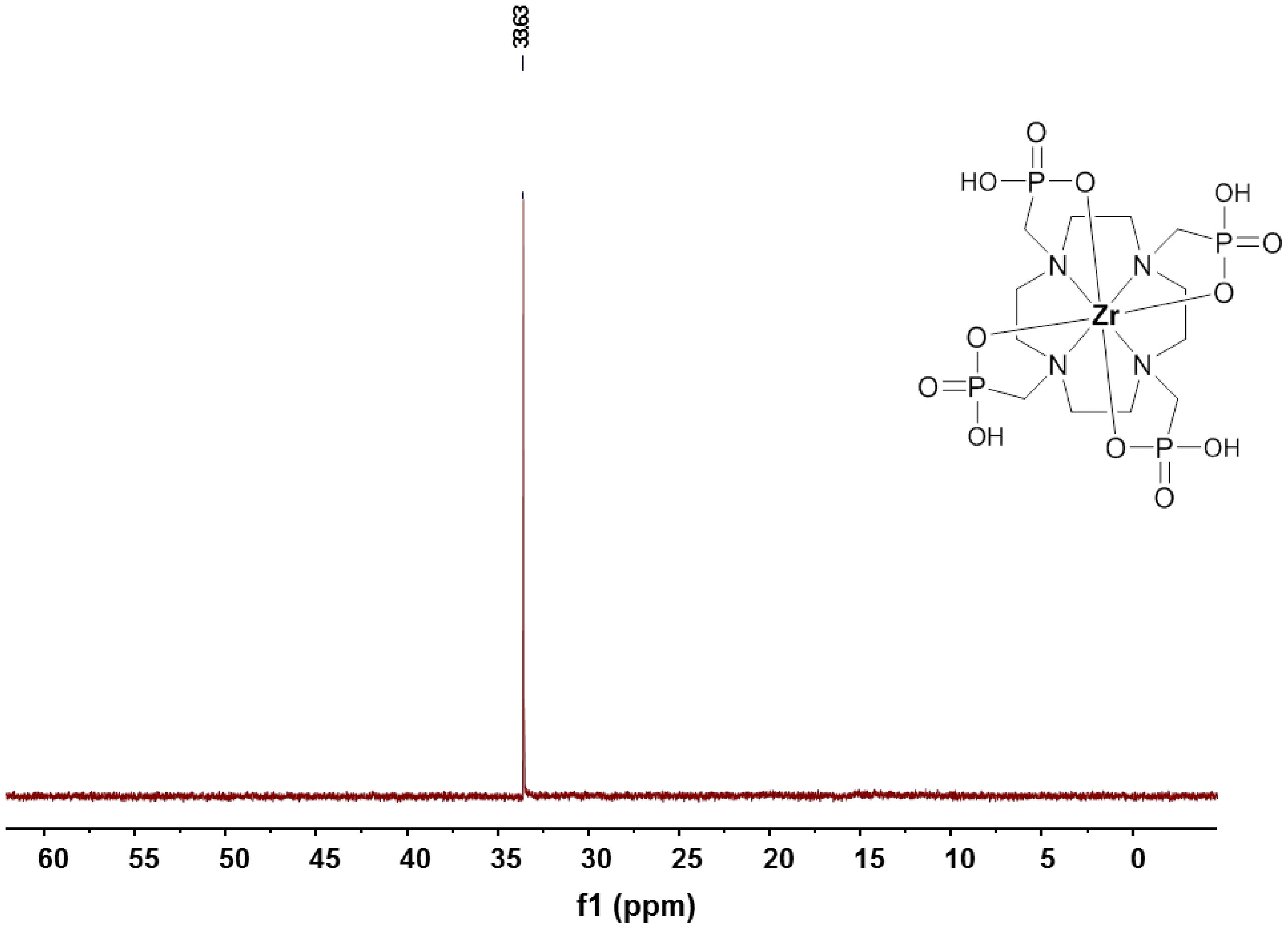

Figure S13. ^{31}P - NMR spectrum (D_2O-500 MHz) with ^{1}H decoupling of the Zr-DOTP complex

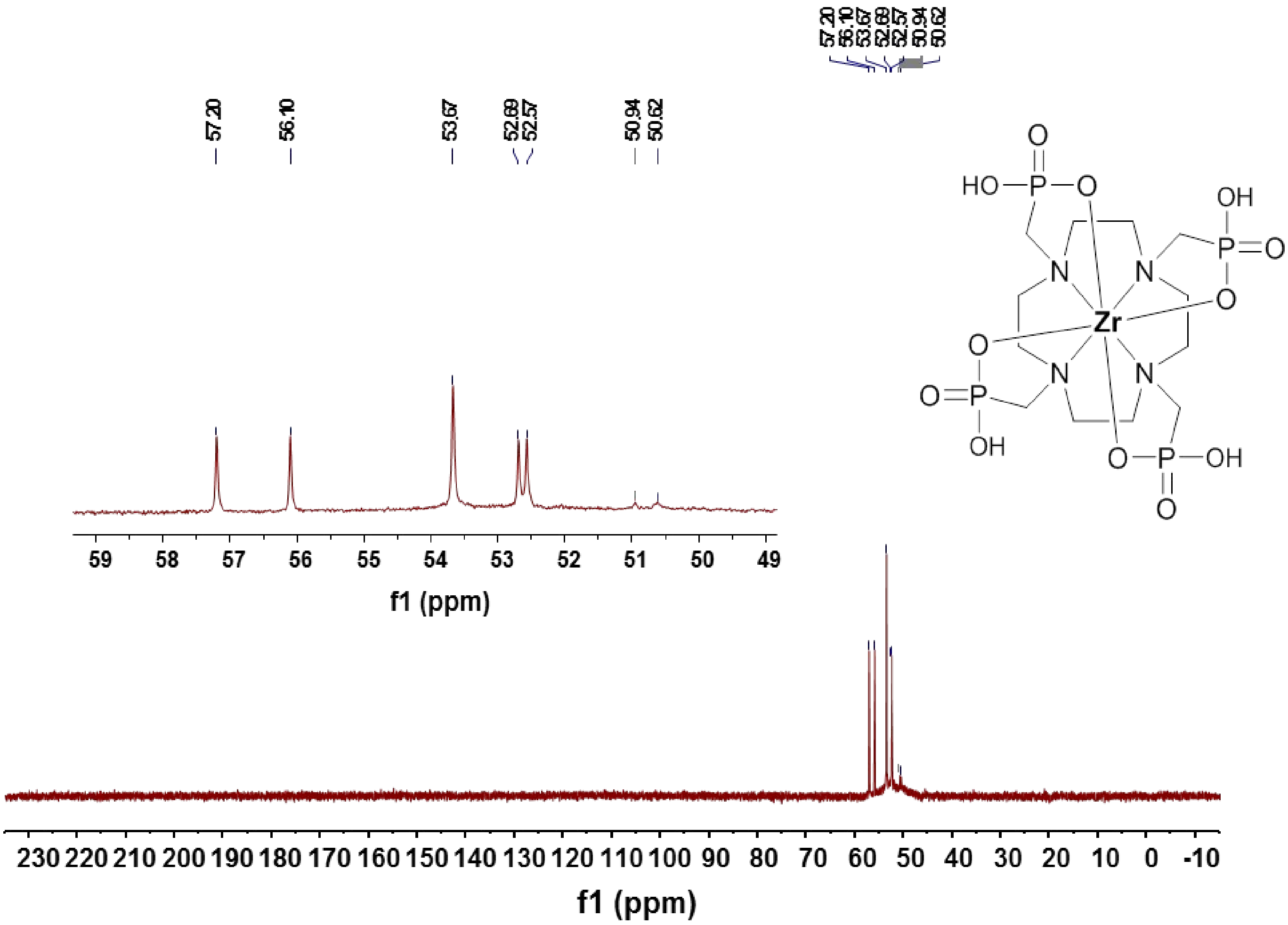

Figure S14. ^{13}**C- NMR spectrum (**D_2O**-126 MHz) of the Zr-DOTP complex**

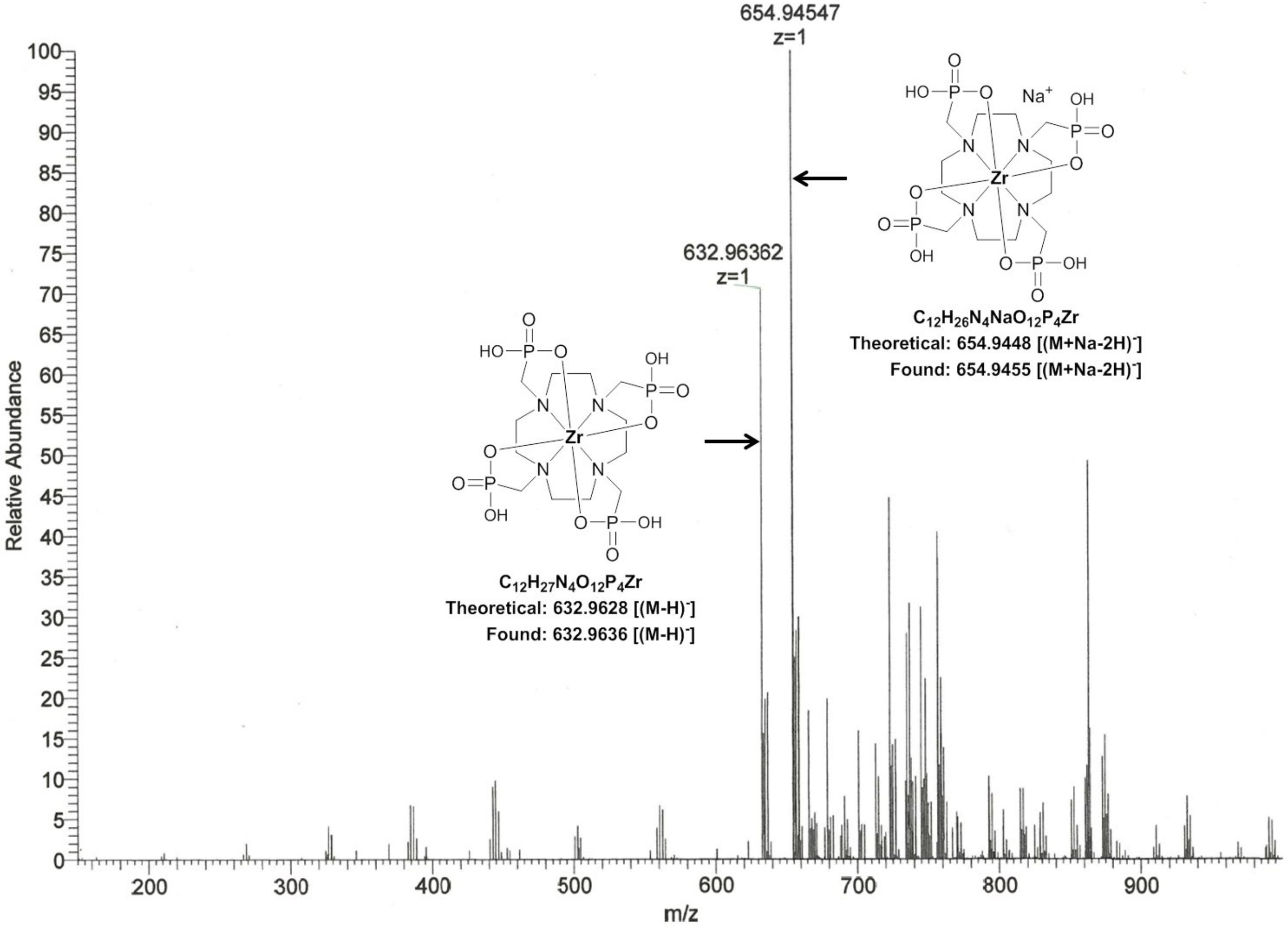

Figure S15. ESI FT-ICR MS (Negative mode) analysis of the Zr-DOTP complex

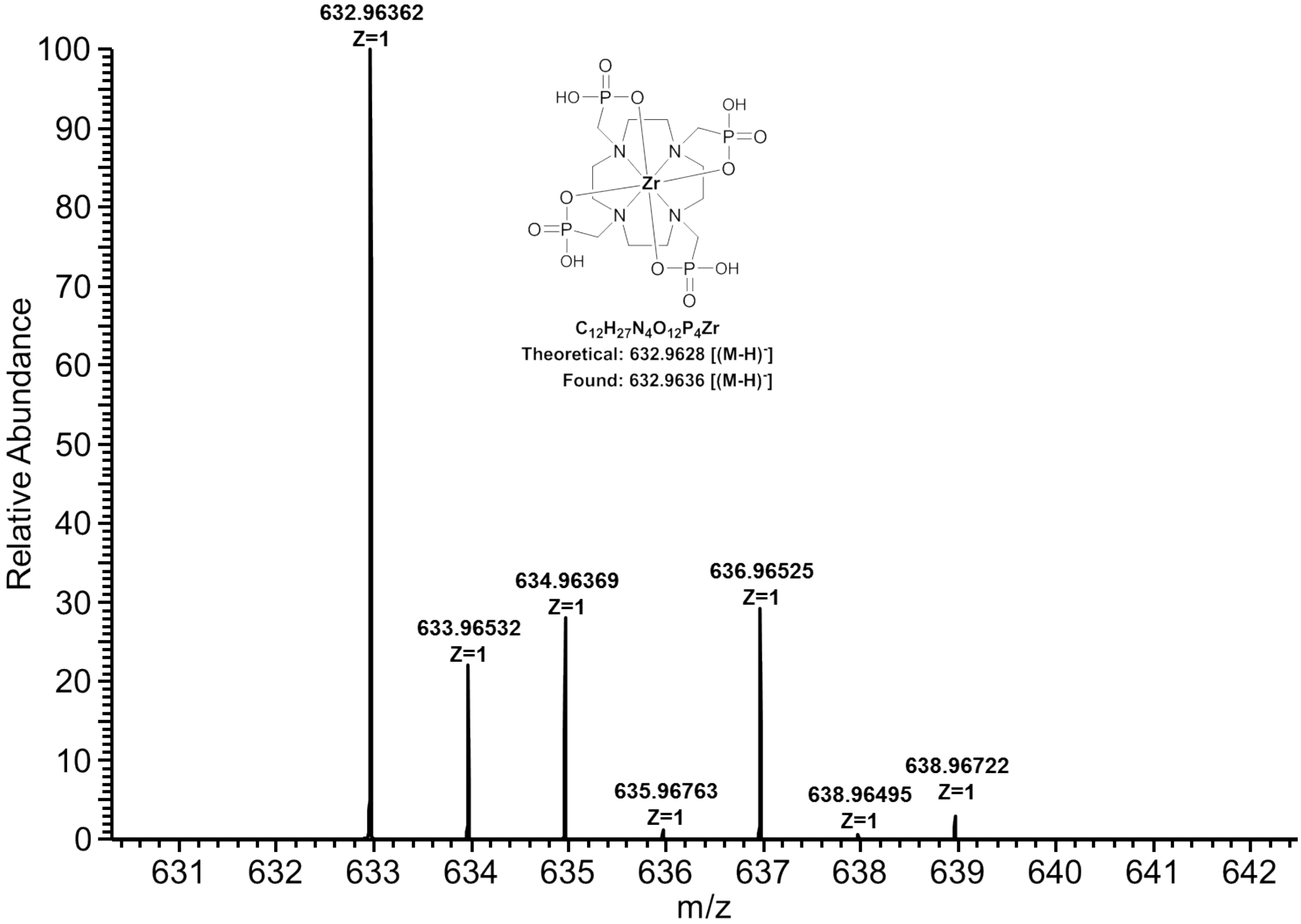

Figure S16. ESI FT-ICR MS (Negative mode) analysis of the Zr-DOTP complex

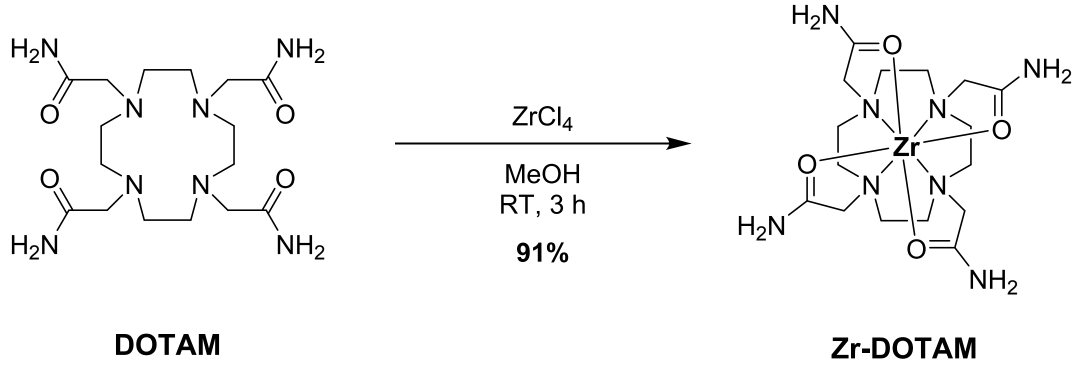

Scheme S3. Synthesis of Zirconium complex of DOTAM

Synthesis of Zr-DOTAM. $ZrCl_4$ (259 mg, 1.11 mmol) was added to a solution of DOTAM (405 mg, 1.01 mmol) in 40 mL of methanol. The resulting clear solution was stirred at room temperature for 3 h, and filtered through a celite bed. The filtrate was subjected to diethyl ether diffusion. The deposited crystals were collected and dried (452 mg, 91% yield). Formation of **Zr-DOTAM** complex was confirmed by NMR spectroscopy (Fig. S17, S18) and HRMS analysis (Fig. S19, S20). ^{1}H NMR (500 MHz, D_2O): δ 4.30 (d, J = 17.5 Hz, 4H), 4.13 (d, J = 18 Hz, 4H), 3.78 (td, J = 14.8, 3.3 Hz, 4H), 3.26 (dd, J = 14.8, 3.8 Hz, 4H), 3.08-2.98 (m, 8H); ^{13}C NMR (126 MHz, D_2O) δ 179.92, 64.52, 57.61, 56.81; HRMS (ESI FT-ICR): Calculated for $C_{16}H_{29}N_8O_4Zr$, 487.1341 [$(M-3H)^-$] Found: 487.1333 [$(M-3H)^-$].

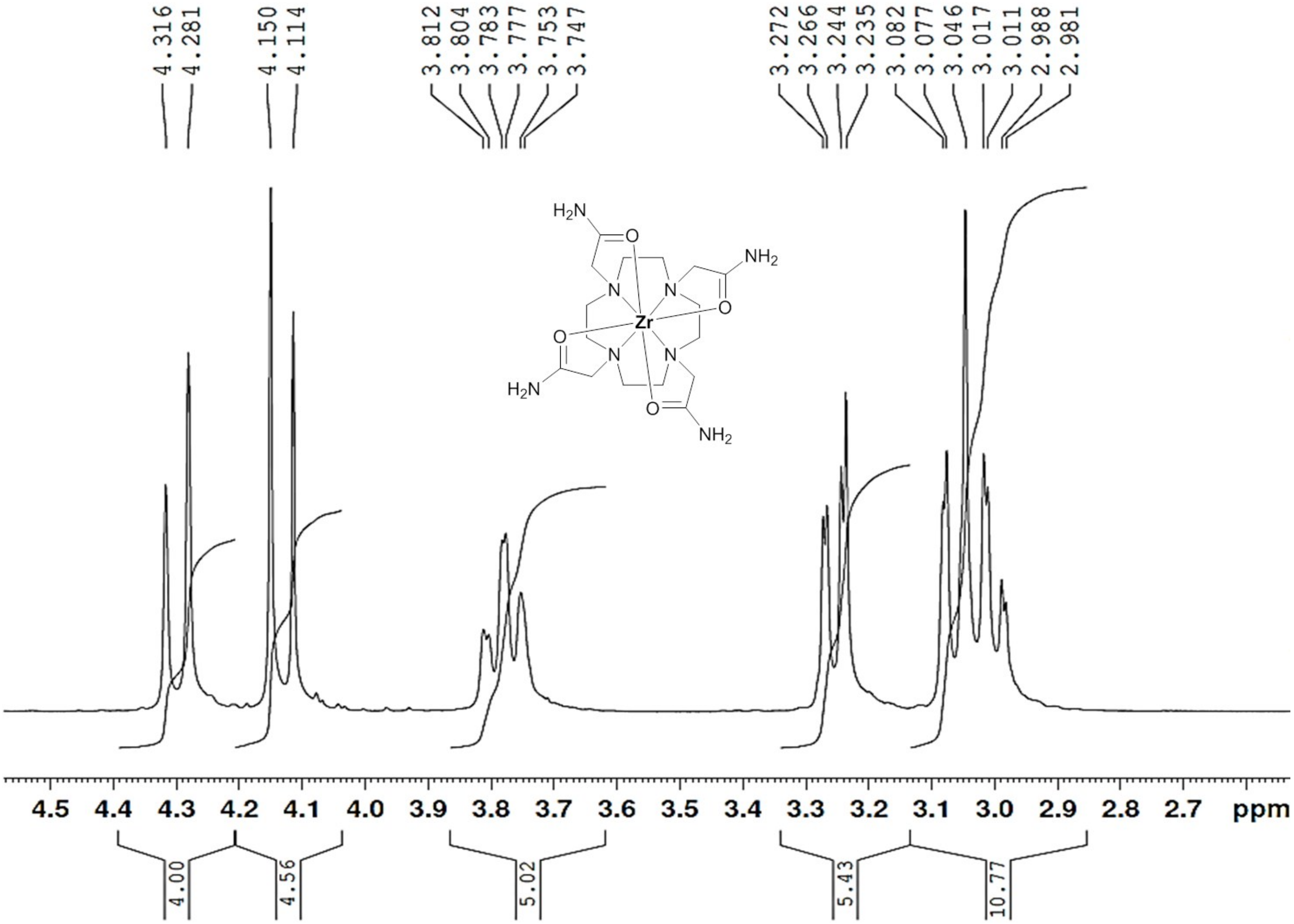

Figure S17. ^{1}H- NMR spectrum (D_2O-500 MHz) of the Zr-DOTAM complex

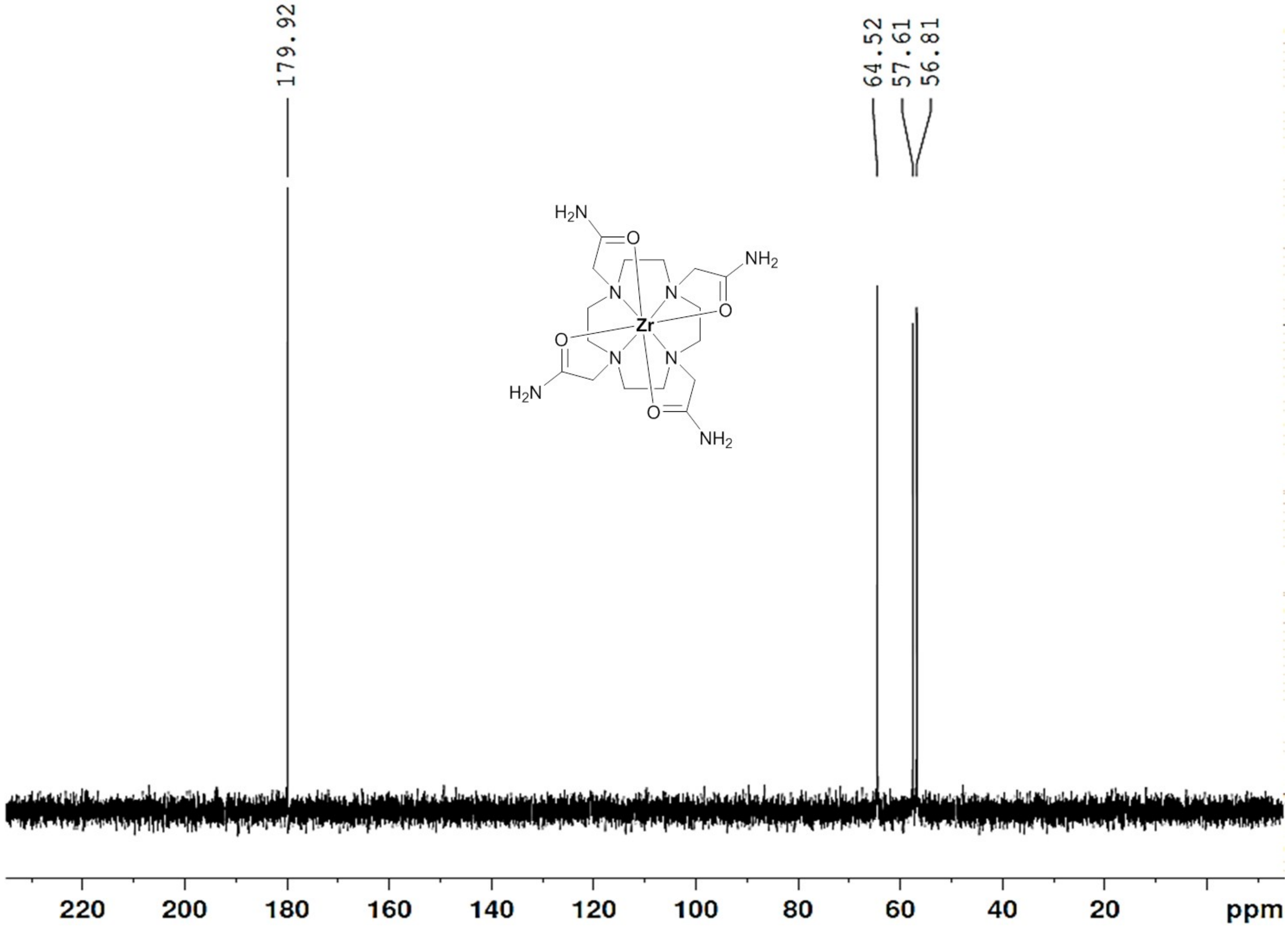

Figure S18. ^{13}C- NMR spectrum (D_2O-126 MHz) of the Zr-DOTAM complex

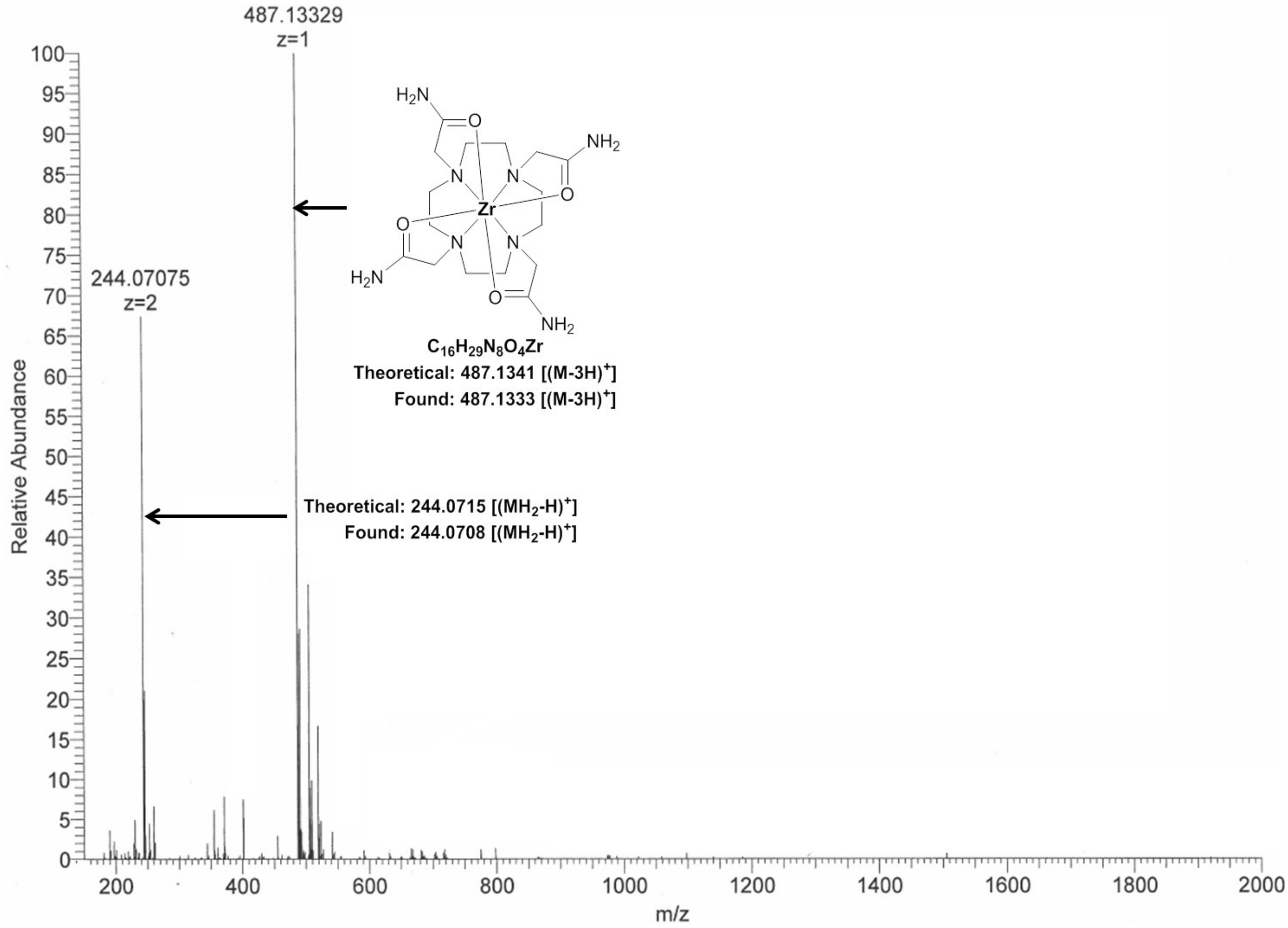

Figure S19. ESI FT-ICR MS (Positive mode) analysis of the Zr-DOTAM complex

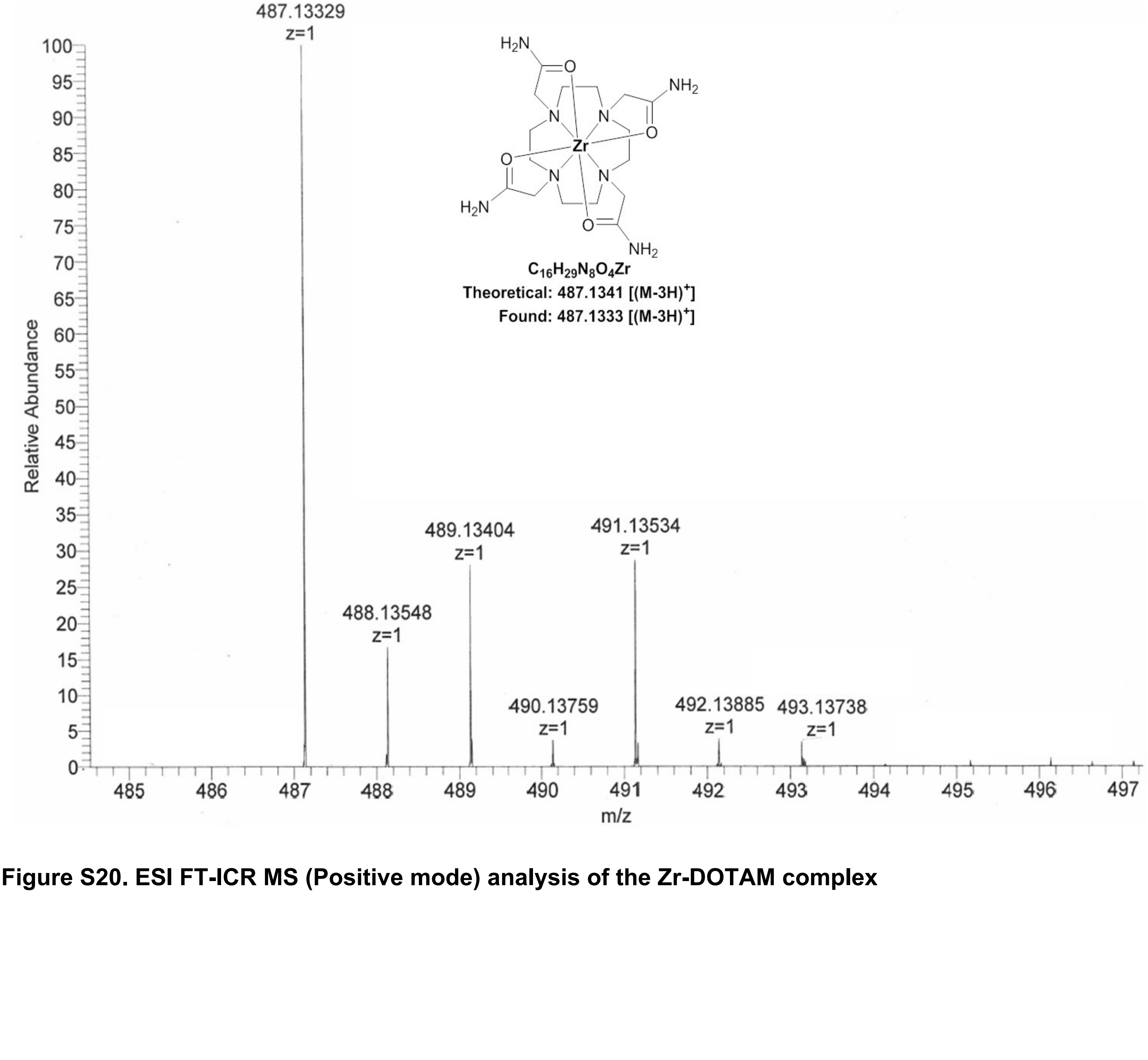

Figure S20. ESI FT-ICR MS (Positive mode) analysis of the Zr-DOTAM complex

Crystal Structure Analysis of Zr-DOTA ($ZrO_8N_4C_{16}H_{24}$ - 4.36 H_2O) (CCDC 1501174).

Experimental Details - Crystallography

Data Collection and Structure Solution. A clear colorless needle-like specimen of $C_{16}H_{32.72}N_4O_{12.36}Zr$, approximate dimensions 0.070 mm x 0.080 mm x 0.420 mm, was used for X-ray crystallographic analysis. The X-ray intensity data were measured on a Bruker APEX CCD system equipped with a graphite monochromator and a Mo Kα sealed x-ray tube (λ = 0.71073 Å). X-rays were provided by a fine-focus sealed x-ray tube operated at 50kV and 30mA.

The total exposure time was 18.57 hours. The frames were integrated with the Bruker SAINT Software[1] package using a narrow-frame algorithm. Integration of the data using a tetragonal unit cell yielded a total of 39,474 reflections to a maximum θ angle of 30.06° (0.71 Å resolution), of which 3278 were independent (average redundancy 12.042, completeness = 99.7%, R_{int} = 3.18%, R_{sig} = 1.51%) and 2866 (87.43%) were greater than 2σ(F^2). The final cell constants of a = 13.0201 (18) Å, b = 13.0201 (18) Å, c = 13.1802 (19) Å, volume = 2234.3 (7) Å^3, are based upon the refinement of the XYZ-centroids of 9915 reflections above 20 σ(I) with 6.93° < 2θ < 60.08°. Data were corrected for absorption effects using the multi-scan method (SADABS).[2] The ratio of minimum to maximum apparent transmission was 0.929. The calculated minimum and maximum transmission coefficients (based on crystal size) are 0.797 and 0.962.

The structure was solved and refined using the Bruker SHELXTL Software Package,[3] using the space group P 4 c c - C_{4v}^{5}, (No. 103) with Z = 4 for the formula unit, $C_{16}H_{32.72}N_4O_{12.36}Zr$. The contents of the asymmetric unit for the refined model, Zr0.5O4N2C8H12 - 2.18 H2O, included 1 ordered (site occupancy 0.25) and 1 disordered (site occupancies: 0.17/0.08) Zr center. For the disordered Zr site, the 2 partial-occupancy $O_8N_4C_{16}H_{24}$ ligands, "DOTA", are rotated about the c axis by ~45° with respect to each other. The final model includes 6 "partial" occupancy water molecules for which 2 oxygens were refined anisotropically and 4 oxygens were refined isotropically; only 3 water hydrogens were included in the structural model. The final structural model incorporated isotropic thermal parameters for all included hydrogen atoms. The hydrogen atoms of the DOTA ligand were included in the structural model as fixed atoms (using idealized sp^3-hybridized geometry and a C-H bond length of 0.99 Å) "riding" on their respective carbon atoms. The isotropic thermal parameters for all

included hydrogen atoms were fixed at values 1.2 times the equivalent isotropic thermal parameter of the oxygen or carbon atom to which they are covalently bonded. The final anisotropic/isotropic full-matrix least-squares refinement on F^2 with 240 variables converged at R_1 = 4.32%, for the observed data and wR_2 = 11.19% for all data. The goodness-of-fit was 1.067. The largest peak in the final difference electron density synthesis was 0.863 $e^-/Å^3$ and the largest hole was -0.816 $e^-/Å^3$ with an RMS deviation of 0.090 $e^-/Å^3$. Based on the final model, the calculated density was 1.695 g/cm^3 and F(000), 1182 e^-.

Refinement Details

Details of crystal data, data collection, and structure refinement are summarized in the tables below.

Computing details

Data collection: Bruker *SMART*; cell refinement: Bruker *APEX2* v2014.11-0; data reduction: Bruker *APEX2* v2014.11-0; program(s) used to solve structure: *SHELXL2014*; program(s) used to refine structure: *SHELXL2014*; molecular graphics: Bruker *APEX2*v2014.11-0; software used to prepare material for publication: Bruker *APEX2*v2014.11-0.[1-5]

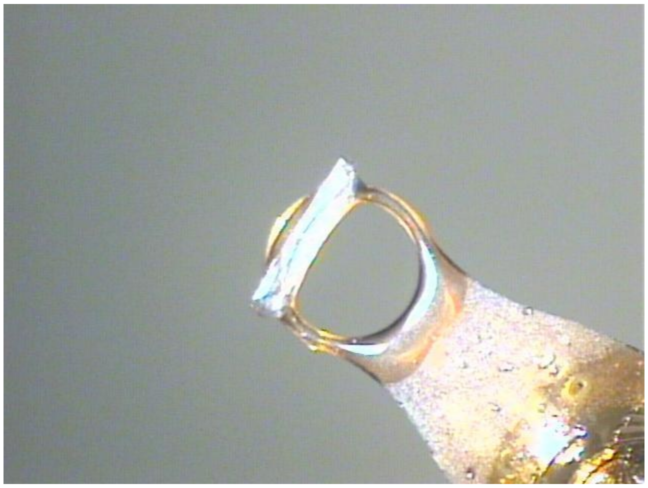

Figure S21. Picture of crystal used for data collection for a31w

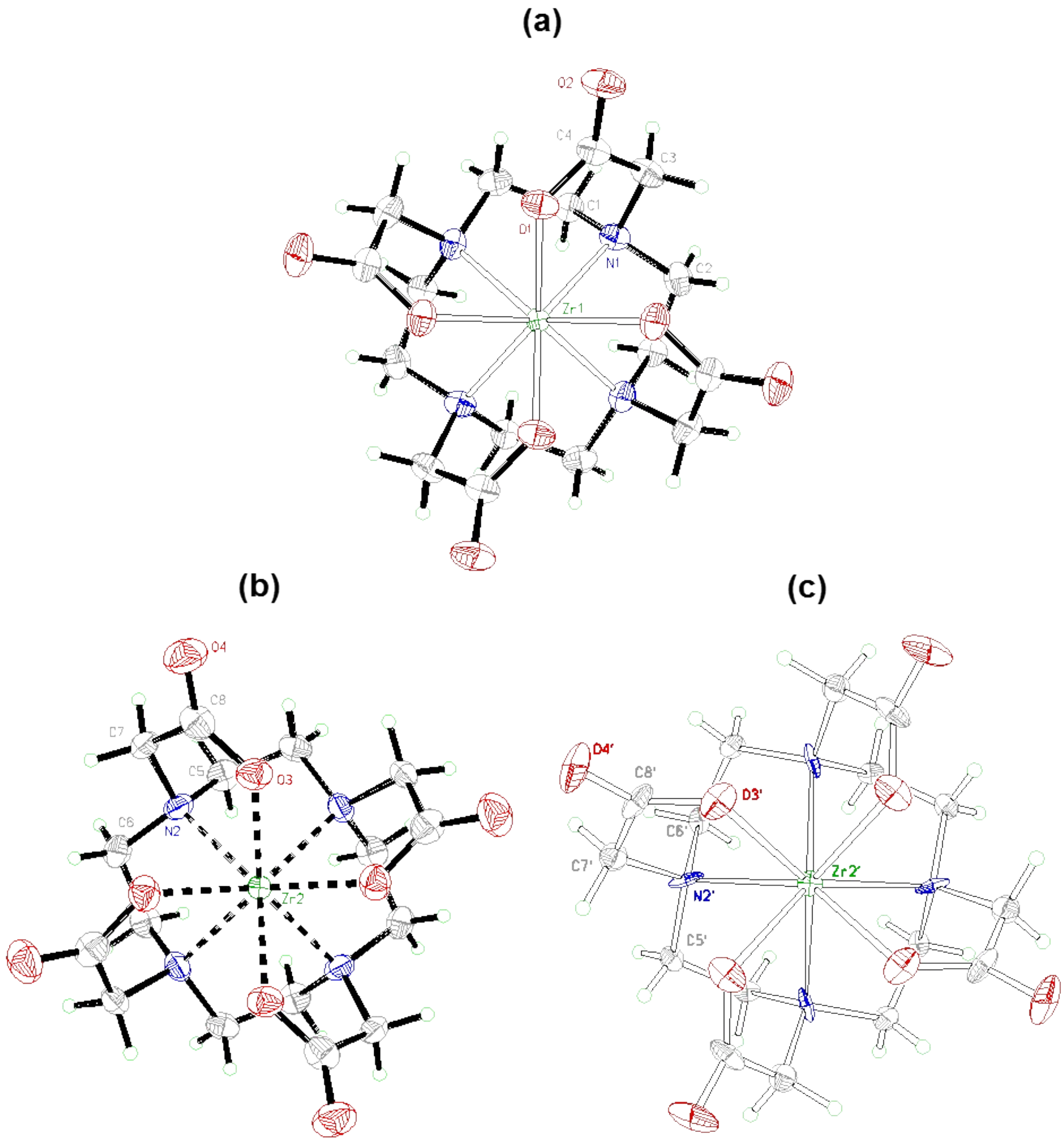

Figure S22. 50% probability plots for the 2 crystallographically-independent Zr centers in the asymmetric unit, Zr0.5O4N2C8H12 - 2.18 H2O. The ordered (Zr1) site is shown in 20a and the disordered (Zr2/Zr2') site is shown in 20b and 20c. The major (0.17 occupancy) Zr2 site is shown in 20b and the minor (0.08 occupancy) Zr2' site is shown in 20c.

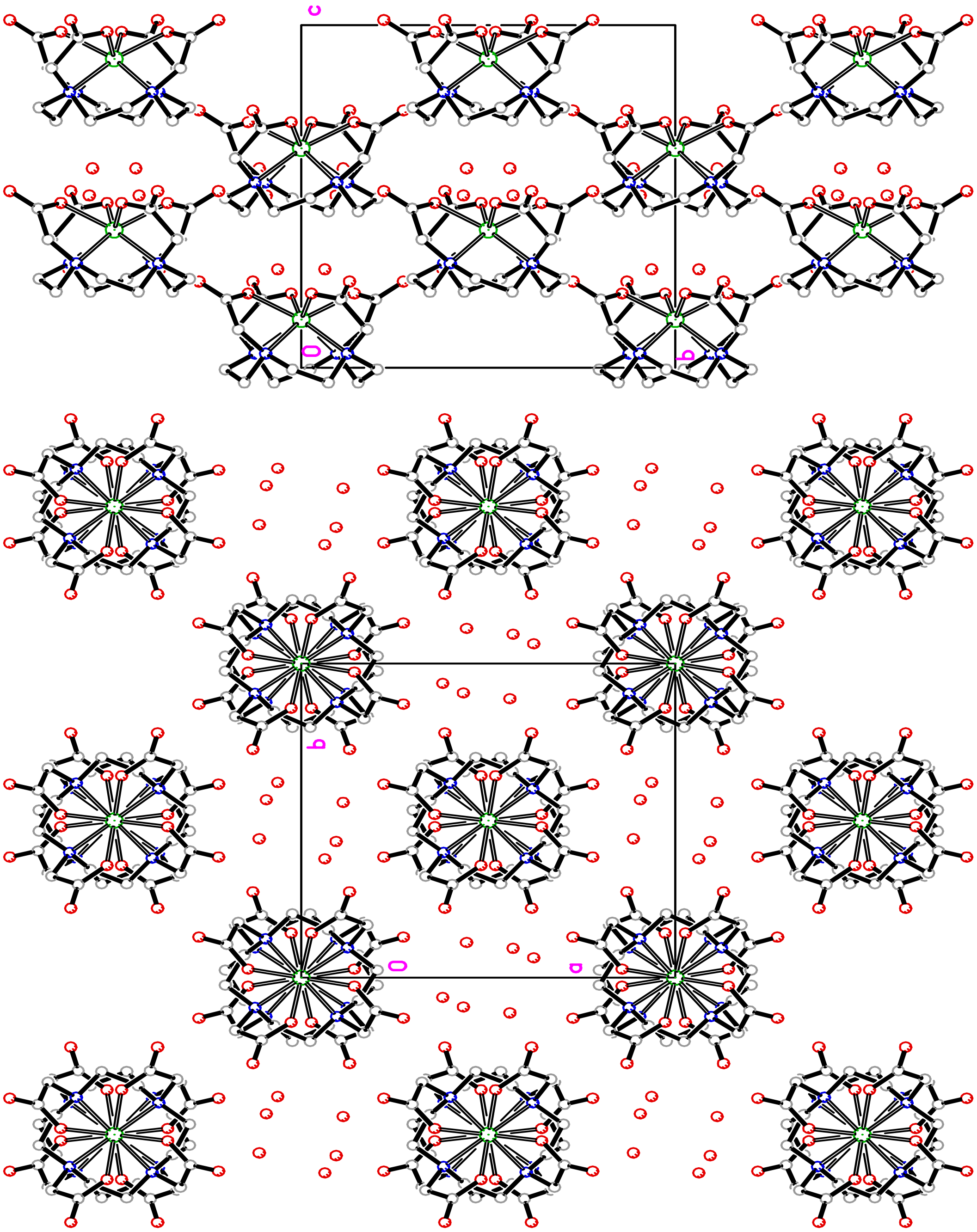

Figure S23. Projections down a and c axes of the unit cell for $ZrO_8N_4C_{16}H_{24}$ – 4.36 H_2O. Hydrogen atoms and selected disordered atoms omitted for clarity.

Table S1. Crystal structure data collection parameters

(a31w)

Crystal data

$C_{16}H_{32.72}N_4O_{12.36}Zr$	D_x = 1.695 Mg m^{-3}
M_r = 570.16	Mo *K*α radiation, λ = 0.71073 Å
Tetragonal, *P*4*cc*	Cell parameters from 9915 reflections
a = 13.0201 (18) Å	θ = 3.5–30.0°
c = 13.1802 (19) Å	μ = 0.56 mm^{-1}
V = 2234.3 (7) Å^3	*T* = 173 K
Z = 4	Needle, colorless
F(000) = 1182	0.42 × 0.08 × 0.07 mm

Data collection

Bruker APEX CCD diffractometer	3278 independent reflections
Radiation source: sealed tube	2866 reflections with *I* > 2□(*I*)
Graphite monochromator	R_{int} = 0.032
ϕ and ω scans	$θ_{max}$ = 30.1°, $θ_{min}$ = 3.5°
Absorption correction: multi-scan Data were corrected for scaling and absorption effects using the multi-scan technique (*SADABS*). The ratio of minimum to maximum apparent transmission was 0.929. The calculated minimum and maximum transmission coefficients (based on crystal size) are 0.797 and 0.962.	*h* = -18→18
T_{min} = 0.693, T_{max} = 0.746	*k* = -18→18
39474 measured reflections	*l* = -18→18

Refinement

Refinement on F^2	Secondary atom site location: difference Fourier map
Least-squares matrix: full	Hydrogen site location: mixed
$R[F^2 > 2\sigma(F^2)] = 0.043$	H atoms treated by a mixture of independent and constrained refinement
$wR(F^2) = 0.112$	$w = 1/[\sigma^2(F_o^2) + (0.0493P)^2 + 3.3847P]$ where $P = (F_o^2 + 2F_c^2)/3$
$S = 1.07$	$(\Delta/\sigma)_{max} < 0.001$
3278 reflections	$\Delta›_{max} = 0.86$ e Å^{-3}
240 parameters	$\Delta›_{min} = -0.82$ e Å^{-3}
5 restraints	Absolute structure: Flack x determined using 1231 quotients [(I+)-(I-)]/[(I+)+(I-)] (Parsons, Flack and Wagner, Acta Cryst. B69 (2013) 249-259).
Primary atom site location: structure-invariant direct methods	Absolute structure parameter: -0.032 (14)

Special details

Geometry. All esds (except the esd in the dihedral angle between two l.s. planes) are estimated using the full covariance matrix. The cell esds are taken into account individually in the estimation of esds in distances, angles, and torsion angles; correlations between esds in cell parameters are only used when they are defined by crystal symmetry. An approximate (isotropic) treatment of cell esds is used for estimating esds involving l.s. planes.
Refinement.<br>Flack x = -0.060(108) by classical fit to all intensities<br>-0.032(14) from 1231 selected quotients (Parsons' method)

Table S2. Fractional atomic coordinates and isotropic or equivalent isotropic displacement parameters (Å²)

	x	*y*	*z*	U_{iso}*/U_{eq}	Occ. (<1)
Zr1	0.0000	0.0000	0.64073 (11)	0.01366 (17)	
O1	0.0273 (3)	0.1420 (2)	0.7169 (3)	0.0289 (7)	
O2	0.1290 (3)	0.2733 (3)	0.7516 (3)	0.0374 (8)	
N1	0.1234 (3)	0.0951 (3)	0.5413 (3)	0.0263 (8)	
C1	0.0725 (3)	0.1523 (4)	0.4566 (3)	0.0256 (8)	
H1A	0.0554	0.1041	0.4010	0.031*	
H1B	0.1199	0.2050	0.4295	0.031*	
C2	0.2028 (4)	0.0237 (4)	0.4948 (4)	0.0320 (10)	
H2A	0.2378	0.0590	0.4380	0.038*	
H2B	0.2551	0.0058	0.5464	0.038*	
C3	0.1757 (4)	0.1679 (4)	0.6113 (3)	0.0294 (10)	
H3A	0.1963	0.2301	0.5731	0.035*	
H3B	0.2387	0.1354	0.6384	0.035*	
C4	0.1061 (4)	0.1992 (3)	0.6996 (3)	0.0249 (8)	
Zr2	0.5000	0.5000	0.40214 (15)	0.0196 (6)	0.68
O3	0.3572 (4)	0.4804 (4)	0.4811 (4)	0.0317 (12)	0.68
O4	0.2211 (5)	0.3841 (5)	0.5157 (5)	0.0450 (15)	0.68
N2	0.3985 (5)	0.3809 (5)	0.3051 (5)	0.0283 (13)	0.68
C5	0.3441 (6)	0.4358 (8)	0.2217 (6)	0.0323 (16)	0.68
H5A	0.3931	0.4508	0.1662	0.039*	0.68
H5B	0.2890	0.3914	0.1942	0.039*	0.68
C6	0.4667 (7)	0.2989 (6)	0.2596 (6)	0.0321 (15)	0.68
H6A	0.4822	0.2463	0.3117	0.039*	0.68
H6B	0.4303	0.2648	0.2030	0.039*	0.68
C7	0.3232 (5)	0.3326 (5)	0.3747 (7)	0.035 (2)	0.68

H7A	0.3519	0.2674	0.4008	0.042*	0.68
H7B	0.2597	0.3164	0.3366	0.042*	0.68
C8	0.2964 (6)	0.4034 (6)	0.4648 (5)	0.0323 (15)	0.68
Zr2'	0.5000	0.5000	0.3626 (3)	0.0126 (8)	0.32
O3'	0.4128 (8)	0.3849 (8)	0.4402 (7)	0.025 (2)	0.32
O4'	0.3906 (10)	0.2214 (8)	0.4844 (7)	0.034 (3)	0.32
N2'	0.5099 (9)	0.3431 (10)	0.2616 (8)	0.020 (2)	0.32
C5'	0.6152 (11)	0.3306 (10)	0.2184 (11)	0.019 (2)	0.32
H5'A	0.6622	0.3032	0.2709	0.023*	0.32
H5'B	0.6135	0.2814	0.1611	0.023*	0.32
C6'	0.4309 (9)	0.3476 (8)	0.1829 (8)	0.015 (2)	0.32
H6'A	0.4220	0.2781	0.1539	0.018*	0.32
H6'B	0.4552	0.3932	0.1278	0.018*	0.32
C7'	0.4885 (10)	0.2560 (10)	0.3326 (10)	0.023 (2)	0.32
H7'A	0.5545	0.2273	0.3567	0.027*	0.32
H7'B	0.4519	0.2011	0.2953	0.027*	0.32
C8'	0.4209 (11)	0.2895 (10)	0.4287 (9)	0.022 (2)	0.32
O5	0.1122 (9)	0.5580 (8)	0.5822 (11)	0.100 (4)	0.68
H51	0.101 (11)	0.565 (12)	0.495 (3)	0.120*	0.68
H52	0.052 (8)	0.591 (10)	0.636 (7)	0.120*	0.68
O6	0.0630 (6)	0.6214 (7)	0.7872 (7)	0.101 (3)	0.9
H61	-0.024 (3)	0.622 (9)	0.798 (10)	0.121*	0.9
O5'	0.0000	0.5000	0.640 (8)	0.076 (14)*	0.14
O6'	-0.028 (5)	0.571 (5)	0.873 (5)	0.076 (16)*	0.1
O7'	0.093 (2)	0.433 (2)	0.5030 (18)	0.080 (7)*	0.25
O7''	0.025 (3)	0.499 (4)	0.436 (2)	0.081 (11)*	0.18

Table S3. Atomic displacement parameters (Å^2)

	U^{11}	U^{22}	U^{33}	U^{12}	U^{13}	U^{23}
Zr1	0.0163 (2)	0.0163 (2)	0.0083 (3)	0.000	0.000	0.000
O1	0.0373 (18)	0.0255 (16)	0.0238 (15)	-0.0056 (13)	-0.0025 (13)	-0.0013 (12)
O2	0.050 (2)	0.0299 (17)	0.0324 (17)	-0.0112 (15)	0.0021 (16)	-0.0107 (14)
N1	0.0313 (19)	0.0250 (17)	0.0228 (17)	-0.0036 (15)	-0.0013 (14)	0.0012 (14)
C1	0.030 (2)	0.031 (2)	0.0159 (15)	0.0002 (17)	0.0041 (15)	0.0076 (15)
C2	0.027 (2)	0.042 (3)	0.027 (2)	0.000 (2)	0.0033 (17)	-0.0005 (19)
C3	0.032 (2)	0.031 (2)	0.0243 (18)	-0.010 (2)	0.0034 (15)	-0.0027 (15)
C4	0.032 (2)	0.0249 (19)	0.0175 (16)	-0.0050 (17)	-0.0005 (15)	-0.0003 (14)
Zr2	0.0176 (5)	0.0176 (5)	0.0235 (16)	0.000	0.000	0.000
O3	0.027 (2)	0.037 (3)	0.031 (3)	-0.002 (2)	-0.002 (2)	-0.007 (2)
O4	0.039 (3)	0.050 (3)	0.046 (3)	-0.009 (3)	0.015 (2)	-0.003 (3)
N2	0.024 (3)	0.027 (3)	0.033 (3)	-0.006 (2)	0.007 (2)	-0.007 (2)
C5	0.036 (4)	0.037 (4)	0.023 (3)	-0.004 (4)	-0.008 (3)	-0.007 (4)
C6	0.040 (4)	0.025 (3)	0.032 (4)	-0.004 (3)	-0.002 (3)	-0.008 (3)
C7	0.026 (3)	0.023 (3)	0.055 (7)	-0.006 (2)	0.009 (3)	-0.006 (3)
C8	0.034 (4)	0.037 (4)	0.026 (3)	0.005 (3)	0.004 (3)	0.000 (3)
Zr2'	0.0170 (12)	0.0170 (12)	0.0038 (10)	0.000	0.000	0.000
O3'	0.035 (5)	0.030 (5)	0.010 (4)	-0.009 (4)	0.006 (4)	0.000 (4)
O4'	0.063 (7)	0.021 (4)	0.020 (4)	-0.011 (5)	0.019 (5)	0.006 (4)
N2'	0.009 (5)	0.034 (7)	0.016 (5)	-0.012 (4)	0.003 (4)	0.002 (4)
C5'	0.019 (6)	0.017 (5)	0.023 (6)	0.004 (5)	0.008 (6)	-0.007 (5)
C6'	0.022 (5)	0.013 (5)	0.009 (4)	0.001 (4)	0.000 (4)	-0.005 (4)
C7'	0.022 (6)	0.022 (6)	0.024 (5)	-0.001 (5)	-0.003 (4)	0.002 (5)
C8'	0.032 (7)	0.022 (6)	0.013 (4)	-0.015 (5)	-0.005 (5)	0.006 (4)
O5	0.103 (8)	0.073 (6)	0.124 (8)	0.037 (6)	-0.022 (7)	-0.035 (6)
O6	0.091 (6)	0.122 (7)	0.091 (5)	0.041 (5)	-0.027 (4)	0.000 (5)

Table S4. Geometric parameters (Å, º) for (a31w)

Zr1—O1	2.133 (3)	C5—C6^{v}	1.486 (12)
Zr1—O1^{i}	2.133 (3)	C5—H5A	0.9900
Zr1—O1^{ii}	2.133 (3)	C5—H5B	0.9900
Zr1—O1^{iii}	2.133 (3)	C6—C5^{vi}	1.486 (12)
Zr1—N1^{ii}	2.415 (4)	C6—H6A	0.9900
Zr1—N1^{i}	2.415 (4)	C6—H6B	0.9900
Zr1—N1	2.415 (4)	C7—C8	1.543 (11)
Zr1—N1^{iii}	2.415 (4)	C7—H7A	0.9900
O1—C4	1.287 (5)	C7—H7B	0.9900
O2—C4	1.221 (5)	Zr2'—O3'^{iv}	2.140 (10)
N1—C3	1.488 (6)	Zr2'—O3'^{vi}	2.140 (10)
N1—C1	1.496 (5)	Zr2'—O3'	2.140 (10)
N1—C2	1.519 (6)	Zr2'—O3'^{v}	2.140 (10)
C1—C2^{iii}	1.503 (7)	Zr2'—N2'^{v}	2.443 (13)
C1—H1A	0.9900	Zr2'—N2'	2.443 (13)
C1—H1B	0.9900	Zr2'—N2'^{vi}	2.443 (13)
C2—C1^{i}	1.503 (7)	Zr2'—N2'^{iv}	2.443 (13)
C2—H2A	0.9900	O3'—C8'	1.255 (17)
C2—H2B	0.9900	O4'—C8'	1.217 (14)
C3—C4	1.531 (6)	N2'—C6'	1.461 (15)
C3—H3A	0.9900	N2'—C5'	1.493 (17)
C3—H3B	0.9900	N2'—C7'	1.496 (17)
Zr2—O3^{iv}	2.146 (5)	C5'—C6'^{vi}	1.469 (17)
Zr2—O3^{v}	2.146 (5)	C5'—H5'A	0.9900
Zr2—O3^{vi}	2.146 (5)	C5'—H5'B	0.9900

Zr2—O3	2.146 (5)	C6'—C5'^{v}	1.469 (17)
Zr2—N2	2.406 (6)	C6'—H6'A	0.9900
Zr2—N2^{iv}	2.406 (6)	C6'—H6'B	0.9900
Zr2—N2^{v}	2.406 (6)	C7'—C8'	1.603 (19)
Zr2—N2^{vi}	2.406 (6)	C7'—H7'A	0.9900
O3—C8	1.295 (9)	C7'—H7'B	0.9900
O4—C8	1.215 (9)	O5—H51	1.16 (3)
N2—C7	1.482 (9)	O5—H52	1.14 (3)
N2—C5	1.490 (10)	O6—H61	1.14 (3)
N2—C6	1.512 (9)	O7''—O7''^{vii}	0.65 (7)
O1—Zr1—O1^{i}	77.22 (8)	C7—N2—C5	110.2 (6)
O1—Zr1—O1^{ii}	123.88 (18)	C7—N2—C6	109.6 (6)
O1^{i}—Zr1—O1^{ii}	77.22 (8)	C5—N2—C6	109.0 (6)
O1—Zr1—O1^{iii}	77.22 (8)	C7—N2—Zr2	107.9 (5)
O1^{i}—Zr1—O1^{iii}	123.88 (18)	C5—N2—Zr2	110.1 (5)
O1^{ii}—Zr1—O1^{iii}	77.22 (8)	C6—N2—Zr2	110.0 (4)
O1—Zr1—N1^{ii}	144.16 (13)	C6^{v}—C5—N2	110.5 (6)
O1^{i}—Zr1—N1^{ii}	138.26 (13)	C6^{v}—C5—H5A	109.6
O1^{ii}—Zr1—N1^{ii}	72.53 (13)	N2—C5—H5A	109.6
O1^{iii}—Zr1—N1^{ii}	76.37 (13)	C6^{v}—C5—H5B	109.6
O1—Zr1—N1^{i}	138.26 (13)	N2—C5—H5B	109.6
O1^{i}—Zr1—N1^{i}	72.53 (13)	H5A—C5—H5B	108.1
O1^{ii}—Zr1—N1^{i}	76.37 (13)	C5^{vi}—C6—N2	110.8 (6)
O1^{iii}—Zr1—N1^{i}	144.16 (13)	C5^{vi}—C6—H6A	109.5
N1^{ii}—Zr1—N1^{i}	72.88 (9)	N2—C6—H6A	109.5
O1—Zr1—N1	72.53 (13)	C5^{vi}—C6—H6B	109.5
O1^{i}—Zr1—N1	76.37 (13)	N2—C6—H6B	109.5

O1^{ii}—Zr1—N1	144.16 (13)	H6A—C6—H6B	108.1
O1^{iii}—Zr1—N1	138.26 (13)	N2—C7—C8	111.9 (6)
N1^{ii}—Zr1—N1	114.28 (18)	N2—C7—H7A	109.2
N1^{i}—Zr1—N1	72.88 (9)	C8—C7—H7A	109.2
O1—Zr1—N1^{iii}	76.37 (13)	N2—C7—H7B	109.2
O1^{i}—Zr1—N1^{iii}	144.16 (13)	C8—C7—H7B	109.2
O1^{ii}—Zr1—N1^{iii}	138.26 (13)	H7A—C7—H7B	107.9
O1^{iii}—Zr1—N1^{iii}	72.53 (13)	O4—C8—O3	124.2 (7)
N1^{ii}—Zr1—N1^{iii}	72.88 (9)	O4—C8—C7	118.9 (7)
N1^{i}—Zr1—N1^{iii}	114.28 (18)	O3—C8—C7	116.9 (6)
N1—Zr1—N1^{iii}	72.88 (9)	O3'^{iv}—Zr2'—O3'^{vi}	76.8 (2)
C4—O1—Zr1	123.4 (3)	O3'^{iv}—Zr2'—O3'	123.0 (5)
C3—N1—C1	110.4 (4)	O3'^{vi}—Zr2'—O3'	76.8 (2)
C3—N1—C2	109.2 (4)	O3'^{iv}—Zr2'—O3'^{v}	76.8 (2)
C1—N1—C2	107.7 (3)	O3'^{vi}—Zr2'—O3'^{v}	123.0 (5)
C3—N1—Zr1	107.2 (3)	O3'—Zr2'—O3'^{v}	76.8 (2)
C1—N1—Zr1	111.4 (3)	O3'^{iv}—Zr2'—N2'^{v}	137.8 (4)
C2—N1—Zr1	111.0 (3)	O3'^{vi}—Zr2'—N2'^{v}	144.9 (4)
N1—C1—C2^{iii}	109.7 (3)	O3'—Zr2'—N2'^{v}	77.3 (4)
N1—C1—H1A	109.7	O3'^{v}—Zr2'—N2'^{v}	72.7 (4)
C2^{iii}—C1—H1A	109.7	O3'^{iv}—Zr2'—N2'	144.9 (4)
N1—C1—H1B	109.7	O3'^{vi}—Zr2'—N2'	77.3 (4)
C2^{iii}—C1—H1B	109.7	O3'—Zr2'—N2'	72.7 (4)
H1A—C1—H1B	108.2	O3'^{v}—Zr2'—N2'	137.8 (4)
C1^{i}—C2—N1	110.4 (4)	N2'^{v}—Zr2'—N2'	72.7 (3)
C1^{i}—C2—H2A	109.6	O3'^{iv}—Zr2'—N2'^{vi}	77.3 (4)
N1—C2—H2A	109.6	O3'^{vi}—Zr2'—N2'^{vi}	72.7 (4)
C1^{i}—C2—H2B	109.6	O3'—Zr2'—N2'^{vi}	137.8 (4)

N1—C2—H2B	109.6	O3'^{v}—Zr2'—N2'^{vi}	144.9 (4)
H2A—C2—H2B	108.1	N2'^{v}—Zr2'—N2'^{vi}	113.9 (5)
N1—C3—C4	111.7 (4)	N2'—Zr2'—N2'^{vi}	72.7 (3)
N1—C3—H3A	109.3	O3'^{iv}—Zr2'—N2'^{iv}	72.7 (4)
C4—C3—H3A	109.3	O3'^{vi}—Zr2'—N2'^{iv}	137.8 (4)
N1—C3—H3B	109.3	O3'—Zr2'—N2'^{iv}	144.9 (4)
C4—C3—H3B	109.3	O3'^{v}—Zr2'—N2'^{iv}	77.3 (4)
H3A—C3—H3B	107.9	N2'^{v}—Zr2'—N2'^{iv}	72.7 (3)
O2—C4—O1	123.6 (4)	N2'—Zr2'—N2'^{iv}	113.9 (5)
O2—C4—C3	119.5 (4)	N2'^{vi}—Zr2'—N2'^{iv}	72.7 (3)
O1—C4—C3	116.9 (4)	C8'—O3'—Zr2'	126.2 (9)
O3^{iv}—Zr2—O3^{v}	76.39 (13)	C6'—N2'—C5'	112.4 (10)
O3^{iv}—Zr2—O3^{vi}	76.39 (13)	C6'—N2'—C7'	110.1 (10)
O3^{v}—Zr2—O3^{vi}	122.0 (3)	C5'—N2'—C7'	109.1 (11)
O3^{iv}—Zr2—O3	122.0 (3)	C6'—N2'—Zr2'	108.4 (8)
O3^{v}—Zr2—O3	76.39 (13)	C5'—N2'—Zr2'	110.3 (8)
O3^{vi}—Zr2—O3	76.39 (13)	C7'—N2'—Zr2'	106.4 (8)
O3^{iv}—Zr2—N2	144.1 (2)	C6'^{vi}—C5'—N2'	109.1 (11)
O3^{v}—Zr2—N2	138.7 (2)	C6'^{vi}—C5'—H5'A	109.9
O3^{vi}—Zr2—N2	76.4 (2)	N2'—C5'—H5'A	109.9
O3—Zr2—N2	72.9 (2)	C6'^{vi}—C5'—H5'B	109.9
O3^{iv}—Zr2—N2^{iv}	72.9 (2)	N2'—C5'—H5'B	109.9
O3^{v}—Zr2—N2^{iv}	76.4 (2)	H5'A—C5'—H5'B	108.3
O3^{vi}—Zr2—N2^{iv}	138.7 (2)	N2'—C6'—C5'^{v}	114.4 (10)
O3—Zr2—N2^{iv}	144.1 (2)	N2'—C6'—H6'A	108.7
N2—Zr2—N2^{iv}	115.8 (3)	C5'^{v}—C6'—H6'A	108.7
O3^{iv}—Zr2—N2^{v}	138.7 (2)	N2'—C6'—H6'B	108.7
O3^{v}—Zr2—N2^{v}	72.9 (2)	C5'^{v}—C6'—H6'B	108.7

O3^{vi}—Zr2—N2^{v}	144.1 (2)	H6'A—C6'—H6'B	107.6
O3—Zr2—N2^{v}	76.4 (2)	N2'—C7'—C8'	113.0 (10)
N2—Zr2—N2^{v}	73.58 (15)	N2'—C7'—H7'A	109.0
N2^{iv}—Zr2—N2^{v}	73.58 (15)	C8'—C7'—H7'A	109.0
O3^{iv}—Zr2—N2^{vi}	76.4 (2)	N2'—C7'—H7'B	109.0
O3^{v}—Zr2—N2^{vi}	144.1 (2)	C8'—C7'—H7'B	109.0
O3^{vi}—Zr2—N2^{vi}	72.9 (2)	H7'A—C7'—H7'B	107.8
O3—Zr2—N2^{vi}	138.7 (2)	O4'—C8'—O3'	128.4 (13)
N2—Zr2—N2^{vi}	73.58 (15)	O4'—C8'—C7'	117.1 (12)
N2^{iv}—Zr2—N2^{vi}	73.58 (15)	O3'—C8'—C7'	114.2 (10)
N2^{v}—Zr2—N2^{vi}	115.8 (3)	H51—O5—H52	120 (4)
C8—O3—Zr2	122.7 (5)		
C3—N1—C1—C2^{iii}	-75.4 (5)	C6—N2—C7—C8	147.1 (6)
C2—N1—C1—C2^{iii}	165.5 (4)	Zr2—N2—C7—C8	27.3 (7)
Zr1—N1—C1—C2^{iii}	43.5 (4)	Zr2—O3—C8—O4	171.5 (6)
C3—N1—C2—C1^{i}	158.8 (4)	Zr2—O3—C8—C7	-8.3 (9)
C1—N1—C2—C1^{i}	-81.3 (5)	N2—C7—C8—O4	164.7 (7)
Zr1—N1—C2—C1^{i}	40.9 (4)	N2—C7—C8—O3	-15.5 (10)
C1—N1—C3—C4	92.9 (4)	C6'—N2'—C5'—C6'^{vi}	79.8 (14)
C2—N1—C3—C4	-148.8 (4)	C7'—N2'—C5'—C6'^{vi}	-157.8 (10)
Zr1—N1—C3—C4	-28.6 (5)	Zr2'—N2'—C5'—C6'^{vi}	-41.3 (11)
Zr1—O1—C4—O2	-170.2 (4)	C5'—N2'—C6'—C5'^{v}	-165.7 (12)
Zr1—O1—C4—C3	7.2 (5)	C7'—N2'—C6'—C5'^{v}	72.5 (14)
N1—C3—C4—O2	-165.2 (4)	Zr2'—N2'—C6'—C5'^{v}	-43.5 (12)
N1—C3—C4—O1	17.3 (6)	C6'—N2'—C7'—C8'	-91.9 (12)
C7—N2—C5—C6^{v}	74.9 (8)	C5'—N2'—C7'—C8'	144.3 (10)
C6—N2—C5—C6^{v}	-164.8 (7)	Zr2'—N2'—C7'—C8'	25.4 (11)

Zr2—N2—C5—C6^{v}	-44.1 (7)	Zr2'—O3'—C8'—O4'	162.6 (12)
C7—N2—C6—C5^{vi}	-159.0 (6)	Zr2'—O3'—C8'—C7'	-11.7 (16)
C5—N2—C6—C5^{vi}	80.3 (9)	N2'—C7'—C8'—O4'	172.2 (12)
Zr2—N2—C6—C5^{vi}	-40.5 (7)	N2'—C7'—C8'—O3'	-12.8 (16)
C5—N2—C7—C8	-93.0 (8)		

Symmetry codes: (i) *y*, -*x*, *z*; (ii) -*x*, -*y*, *z*; (iii) -*y*, *x*, *z*; (iv) -*x*+1, -*y*+1, *z*; (v) *y*, -*x*+1, *z*; (vi) -*y*+1, *x*, *z*; (vii) -*x*, -*y*+1, *z*.

Table S5. Hydrogen-bond geometry (Å, º) for (a31w)

D—H···*A*	*D*—H	H···*A*	*D*···*A*	*D*—H···*A*
O5—H52···O6	1.14 (3)	2.04 (10)	2.897 (15)	129 (9)
O6—H61···O2^{vii}	1.14 (3)	2.03 (10)	2.890 (8)	129 (9)

Symmetry code: (vii) -*x*, -*y*+1, *z*.

Least-squares planes (x,y,z in crystal coordinates) and deviations from them

(* indicates atom used to define plane)

0.0000 (0.0000) x + 0.0000 (0.0000) y + 13.1802 (0.0019) z = 9.4485 (0.0037)

* 0.0000 (0.0000) O1

* 0.0000 (0.0000) O1_$1

* 0.0000 (0.0000) O1_$2

* 0.0000 (0.0000) O1_$3

-1.0036 (0.0033) Zr1

Rms deviation of fitted atoms = 0.0000

0.0000 (0.0000) x + 0.0000 (0.0000) y + 13.1802 (0.0019) z = 7.1345 (0.0040)

Angle to previous plane (with approximate esd) = 0.000 (0.008)

* 0.0000 (0.0000) N1

* 0.0000 (0.0000) N1_$1

* 0.0000 (0.0000) N1_$2

* 0.0000 (0.0000) N1_$3

1.3104 (0.0038) Zr1

Rms deviation of fitted atoms = 0.0000

0.0000 (0.0000) x + 0.0000 (0.0000) y + 13.1802 (0.0019) z = 6.3413 (0.0054)

Angle to previous plane (with approximate esd) = 0.000 (0.008)

* 0.0000 (0.0000) O3_a

* 0.0000 (0.0000) O3_$4a

* 0.0000 (0.0000) O3_$5a

* 0.0000 (0.0000) O3_$7a

-1.0410 (0.0056) Zr2_a

Rms deviation of fitted atoms = 0.0000

0.0000 (0.0000) x + 0.0000 (0.0000) y + 13.1802 (0.0019) z = 4.0211 (0.0067)

Angle to previous plane (with approximate esd) = 0.000 (0.008)

* 0.0000 (0.0000) N2_a

* 0.0000 (0.0000) N2_$4a

* 0.0000 (0.0000) N2_$5a

* 0.0000 (0.0000) N2_$7a

1.2792 (0.0068) Zr2_a

Rms deviation of fitted atoms = 0.0000

0.0000 (0.0000) x + 0.0000 (0.0000) y + 13.1802 (0.0019) z = 5.8016 (0.0089)

Angle to previous plane (with approximate esd) = 0.000 (0.008)

* 0.0000 (0.0000) O3'_b

*   0.0000 (0.0000) O3'_$4b

*   0.0000 (0.0000) O3'_$5b

*   0.0000 (0.0000) O3'_$7b

-1.0218 (0.0100) Zr2'_b

Rms deviation of fitted atoms =   0.0000

0.0000 (0.0000) x + 0.0000 (0.0000) y + 13.1802 (0.0019) z = 3.4473 (0.0109)

Angle to previous plane (with approximate esd) = 0.000 (0.008)

*   0.0000 (0.0000) N2'_b

*   0.0000 (0.0000) N2'_$4b

*   0.0000 (0.0000) N2'_$5b

*   0.0000 (0.0000) N2'_$7b

1.3325 (0.0115) Zr2'_b

Rms deviation of fitted atoms =   0.0000

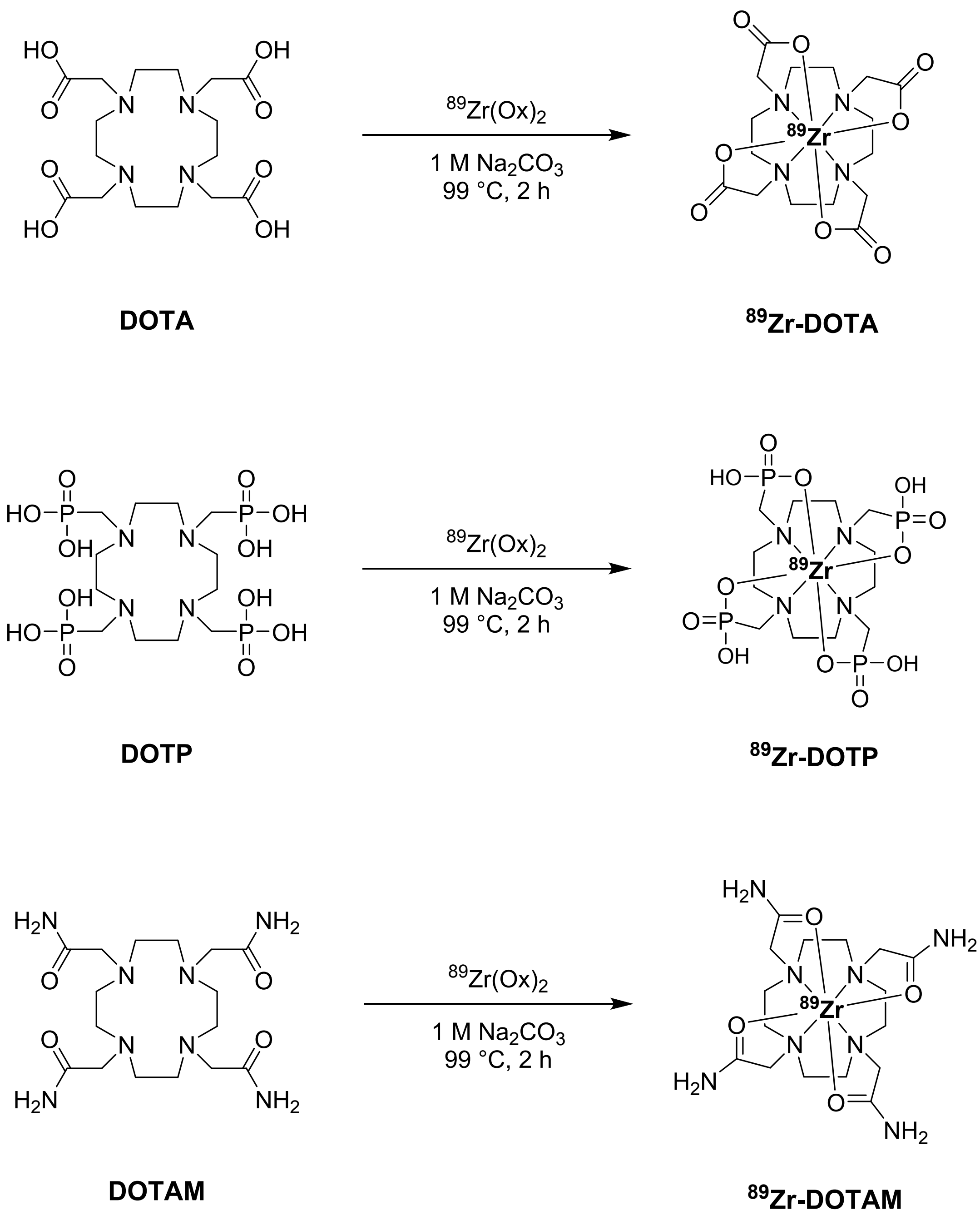

Scheme S4. Radiochemical synthesis of ^{89}Zr-DOTA, ^{89}Zr-DOTP, and ^{89}Zr-DOTAM using $^{89}Zr(ox)_2$

Radiolabeling of Tetraazamacrocyclic ligands (DOTA, DOTP, and DOTAM) with $^{89}Zr(ox)_2$. The complexation of ^{89}Zr with tetraazamacrocyclic ligands (DOTA, DOTP, and DOTAM) was achieved by reacting 10-50 µg (1-5 µL, 10 mg/mL in water) of each ligand with an aliquot of $^{89}Zr(ox)_2$ (0.6 mCi, 22.2 MBq) diluted in 100 µL of water and pH adjusted to 7-7.5 using 1 M Na_2CO_3. Reactions were incubated at 99ºC for 2 h in a thermomixer (550 rpm). Formation of ^{89}Zr-**DOTA**, ^{89}Zr-**DOTP**, and ^{89}Zr-**DOTAM** complexes was monitored by radio-TLC using Varian ITLC-SA strips and 0.1 M EDTA (pH 5) as the mobile phase.

Table S6. Summary of optimized radiochemistry conditions to prepare ^{89}Zr-complexes with $^{89}Zr(ox)_2$

**Radiochemistry conditions**	**Ligand (n = 20)**			
	**DOTA**	**DOTP**	**DOTAM**	**DFO**
Quantity (µg)	10-50	10-50	10-50	10
Temperature (°C)	99	99	99	24
Reaction time (min)	120	120	120	15
Reaction pH	7.0-7.5	7.0-7.5	7.0-7.5	7.0-7.5
Radiochemical yield (%)	65 ± 9.6	70 ± 10.6	9 ± 1.3	100

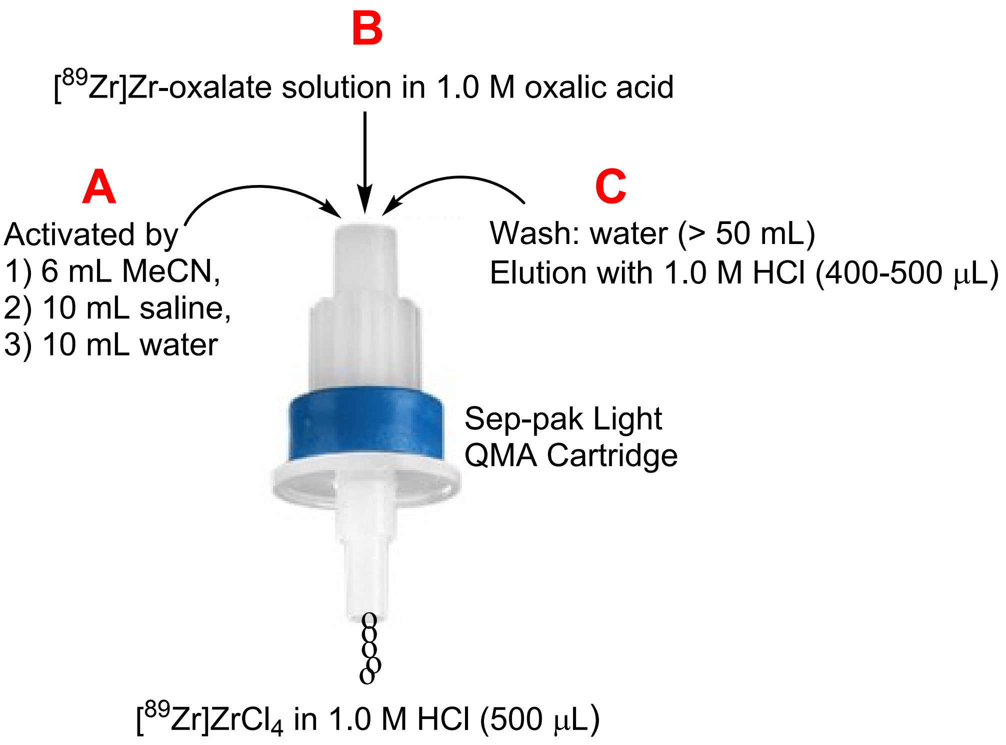

Figure S24. Schematic diagram for the production of $^{89}ZrCl_4$ from $^{89}Zr(ox)_2$

Preparation of [^{89}Zr]Zr-chloride. $^{89}ZrCl_4$ was produced using a procedure modified from the literature. Briefly, a [^{89}Zr]Zr-oxalate solution in 1.0 M oxalic acid was loaded onto an activated Waters Sep-pak Light accell plus QMA strong anion exchange cartridge (300 Å pore size, 37–55 µm particle size, 230 µeq/gram ion exchange capacity), pre-washed with 6 ml MeCN, 10 ml 0.9% saline and 10 ml water. The cartridge was then washed with water (>50 ml) to remove oxalic acid and the activity eluted with 100% recovery of ^{89}Zr by chloride ion exchange with 400–500 µl of 1.0 M HCl(aq.).

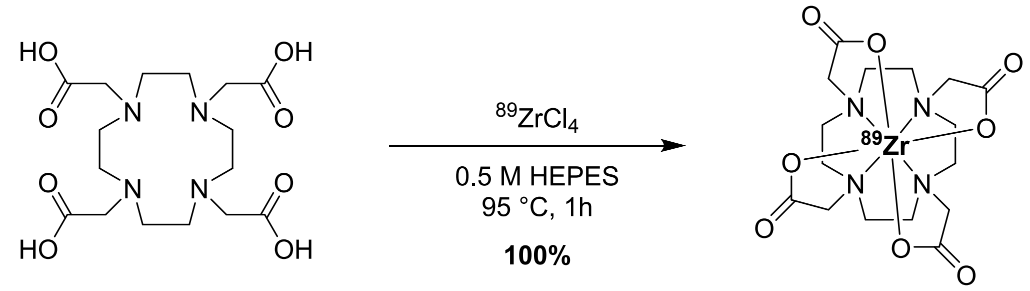

DOTA **^{89}Zr-DOTA**

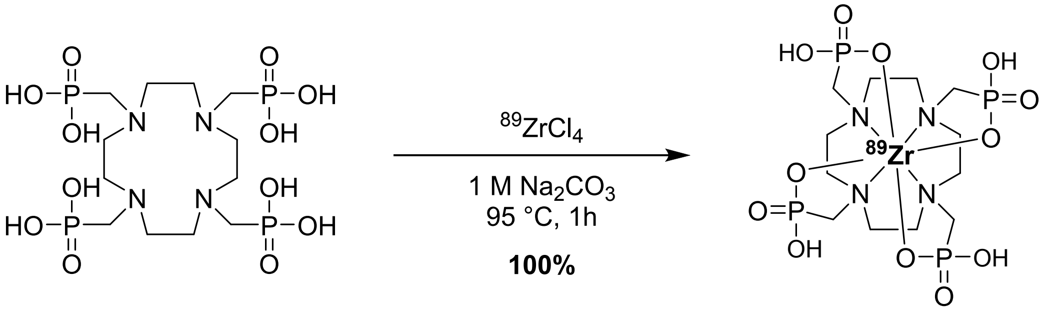

DOTP **^{89}Zr-DOTP**

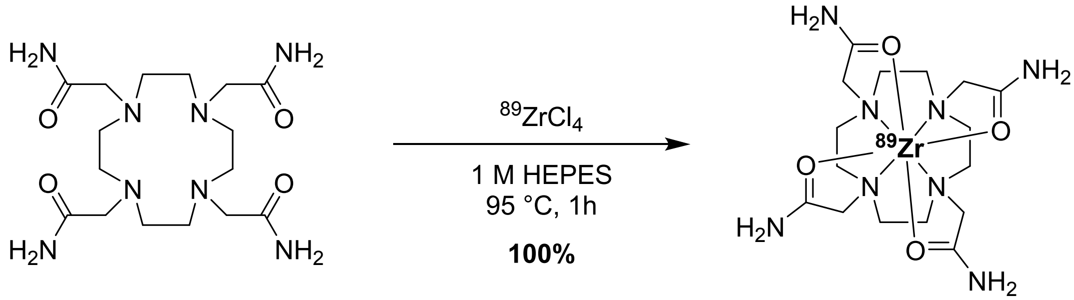

DOTAM **^{89}Zr-DOTAM**

Scheme S5. Radiochemical synthesis of ^{89}Zr-DOTA, ^{89}Zr-DOTP, and ^{89}Zr-DOTAM using $^{89}ZrCl_4$

Radiolabeling of Tetraazamacrocyclic ligand (DOTA, DOTP, and DOTAM) with $^{89}ZrCl_4$.

^{89}Zr-DOTA: Complexing ^{89}Zr with the tetraazamacrocyclic ligand DOTA was achieved by reacting 10 μg (10 μL, 1.0 mg/mL in water) of ligand DOTA with an aliquot of $^{89}ZrCl_4$ (1.1 mCi, 40.7 MBq) diluted in 100 μL of 0.5 M HEPES (pH 7.2) followed by 45 min incubation at 90ºC in a thermomixer (550 rpm). Formation of ^{89}Zr-DOTA complex was monitored by radio-TLC using a mobile phase consisting of 1:1 MeOH:10% NH_4Cl on C-18 plates and 0.1 M EDTA (pH 5) on Varian ITLC-SA strips. In the C-18 system, un-chelated ^{89}Zr remained at the origin (R_f = 0), while ^{89}Zr-DOTA complex moved near the solvent front (R_f = 0.85-0.90). In the ITLC-SA system, free ^{89}Zr formed a complex with EDTA and eluted with the solvent front (R_f = 1), while ^{89}Zr-DOTA complex moves from origin (R_f = 0.35-0.40) (Fig. S25). The identity of the radioactive complex ^{89}Zr-DOTA was further confirmed by comparing its radio-HPLC elution profile to the UV-HPLC spectrum of nonradioactive ^{Nat}Zr-DOTA (Fig. S26).

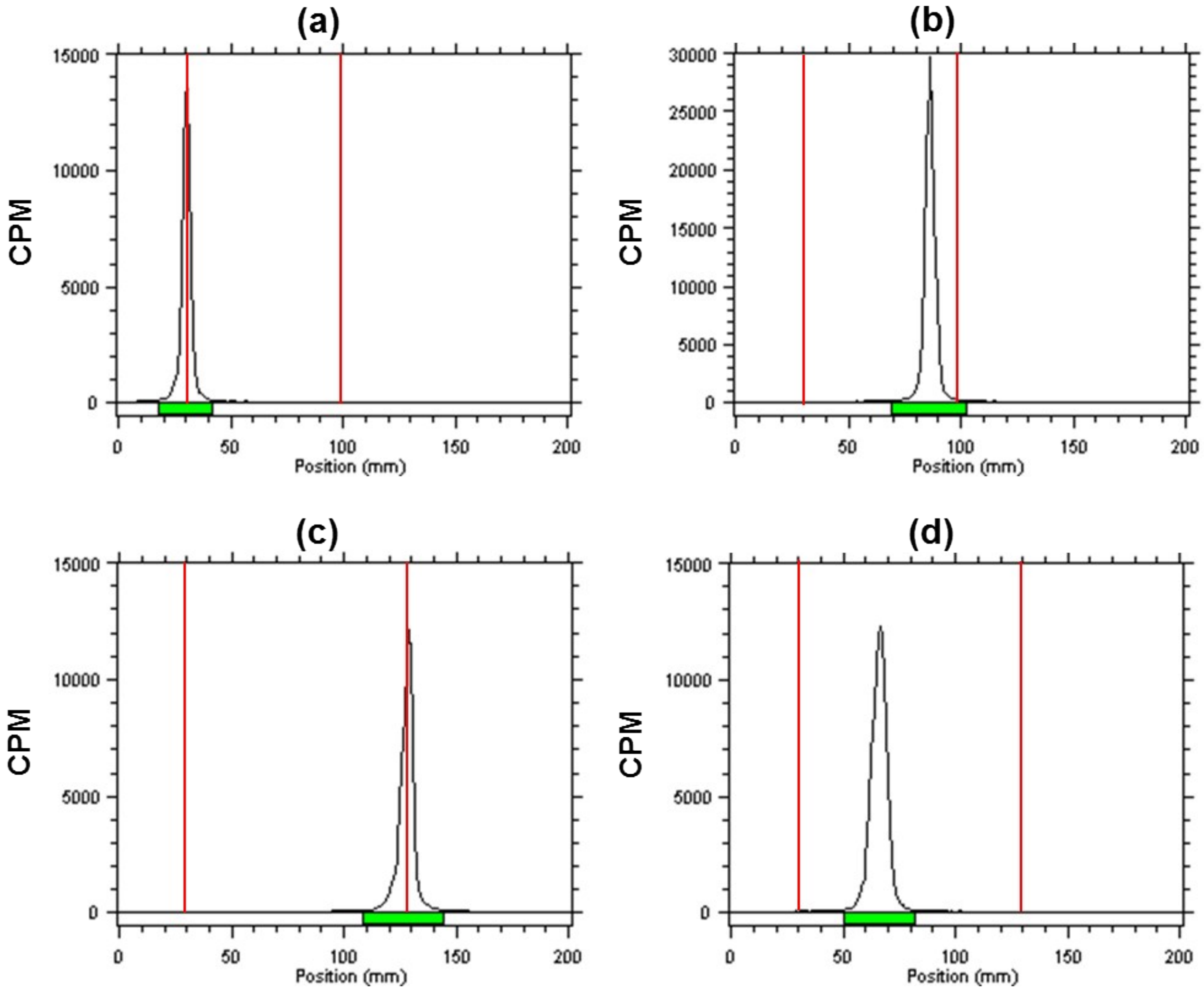

Figure S25. Quality control of ^{89}Zr-DOTA by radio-TLC. C-18-TLC of $^{89}ZrCl_4$ (a), ^{89}Zr-DOTA (b), and ITLC-SA of $^{89}ZrCl_4$ (c), ^{89}Zr-DOTA (d)

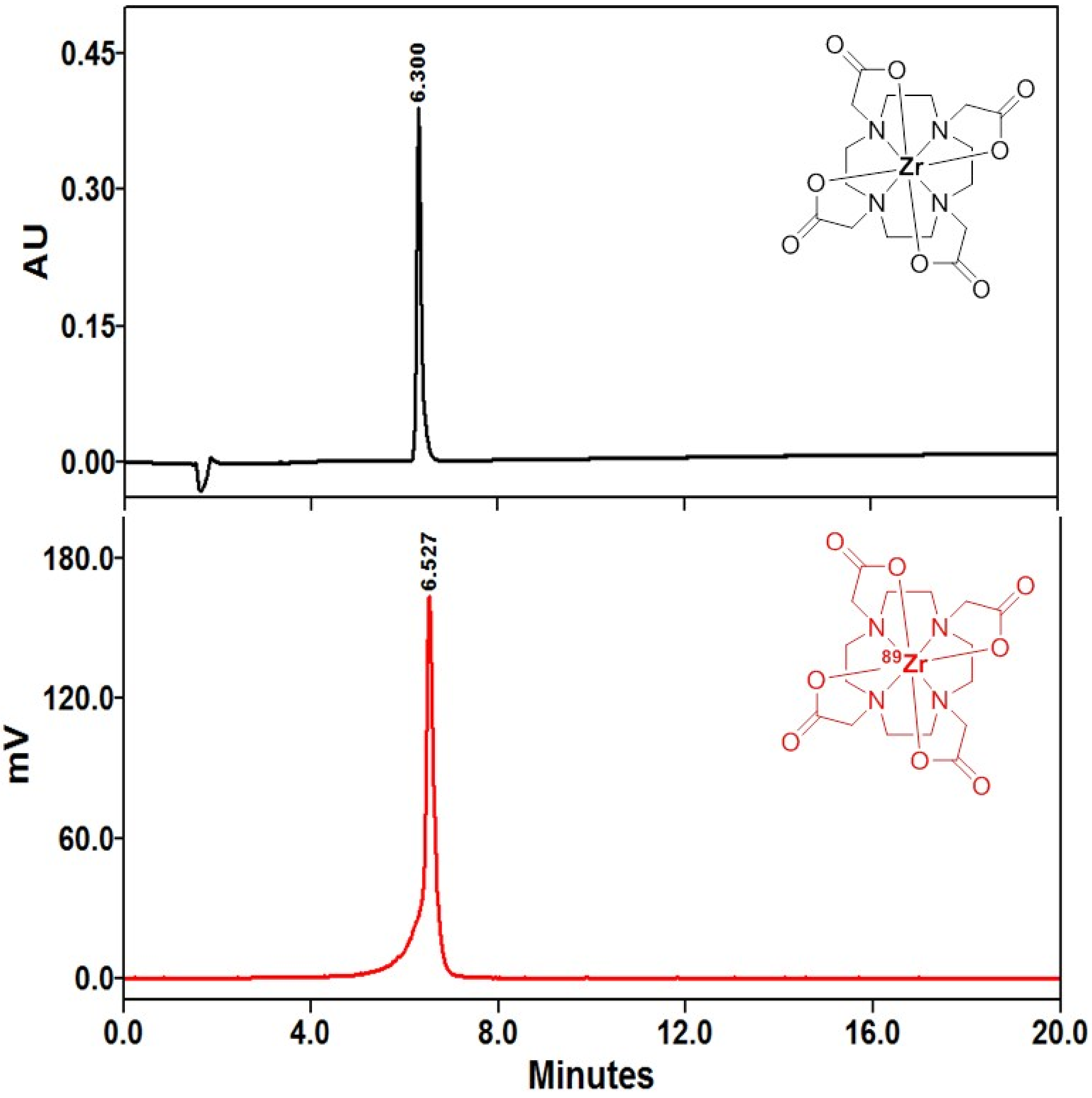

Figure S26. Quality control of ^{89}Zr-DOTA by radio-HPLC. UV-HPLC chromatogram (201 nm) of nonradioactive ^{Nat}Zr-DOTA complex (top) compared with radio-HPLC chromatogram of ^{89}Zr-DOTA (bottom)

^{89}Zr-DOTP: Complexation of ^{89}Zr by the tetraazamacrocyclic ligand DOTP was achieved by reacting 10 μg (10 μL, 1.0 mg/mL in water) of the ligand DOTP with an aliquot of $^{89}ZrCl_4$ (1.1 mCi, 40.7 MBq) diluted in 100 μL of water and pH adjusted to 7-7.5 using 1 M Na_2CO_3. The reactions were incubated at 90ºC for 45 min in a thermomixer (550 rpm). Formation of ^{89}Zr-DOTP complex was monitored by radio-TLC using a mobile phase consisting of 6:4 MeOH:5% NH_4OH on C-18 plates and 0.1 M EDTA (pH 5) on Varian ITLC-SA strips. In the C-18 system, un-chelated ^{89}Zr remained at the origin (R_f = 0), while the ^{89}Zr-DOTP complex moved near the solvent front (R_f = 0.75-0.85). In the ITLC-SA system, free ^{89}Zr formed a complex with EDTA and eluted with the solvent front (R_f = 1), while the ^{89}Zr-DOTP complex remained near the origin (R_f = 0.15-0.20) (Fig. S27). The identity of the radioactive complex ^{89}Zr-DOTP was further confirmed by comparing its radio-HPLC elution profile to the UV-HPLC spectrum of nonradioactive ^{Nat}Zr-DOTP (Fig. S28).

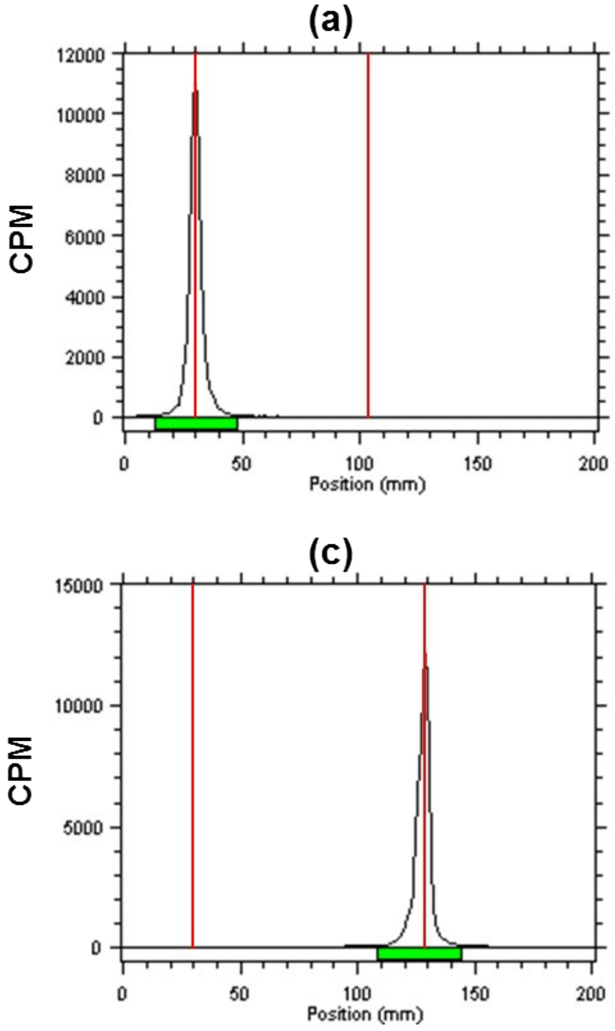

Figure S27. Quality control of ^{89}Zr-DOTP by radio-TLC. C-18-TLC of $^{89}ZrCl_4$ (a), ^{89}Zr-DOTP (b), and ITLC-SA of $^{89}ZrCl_4$ (c), ^{89}Zr-DOTP (d)

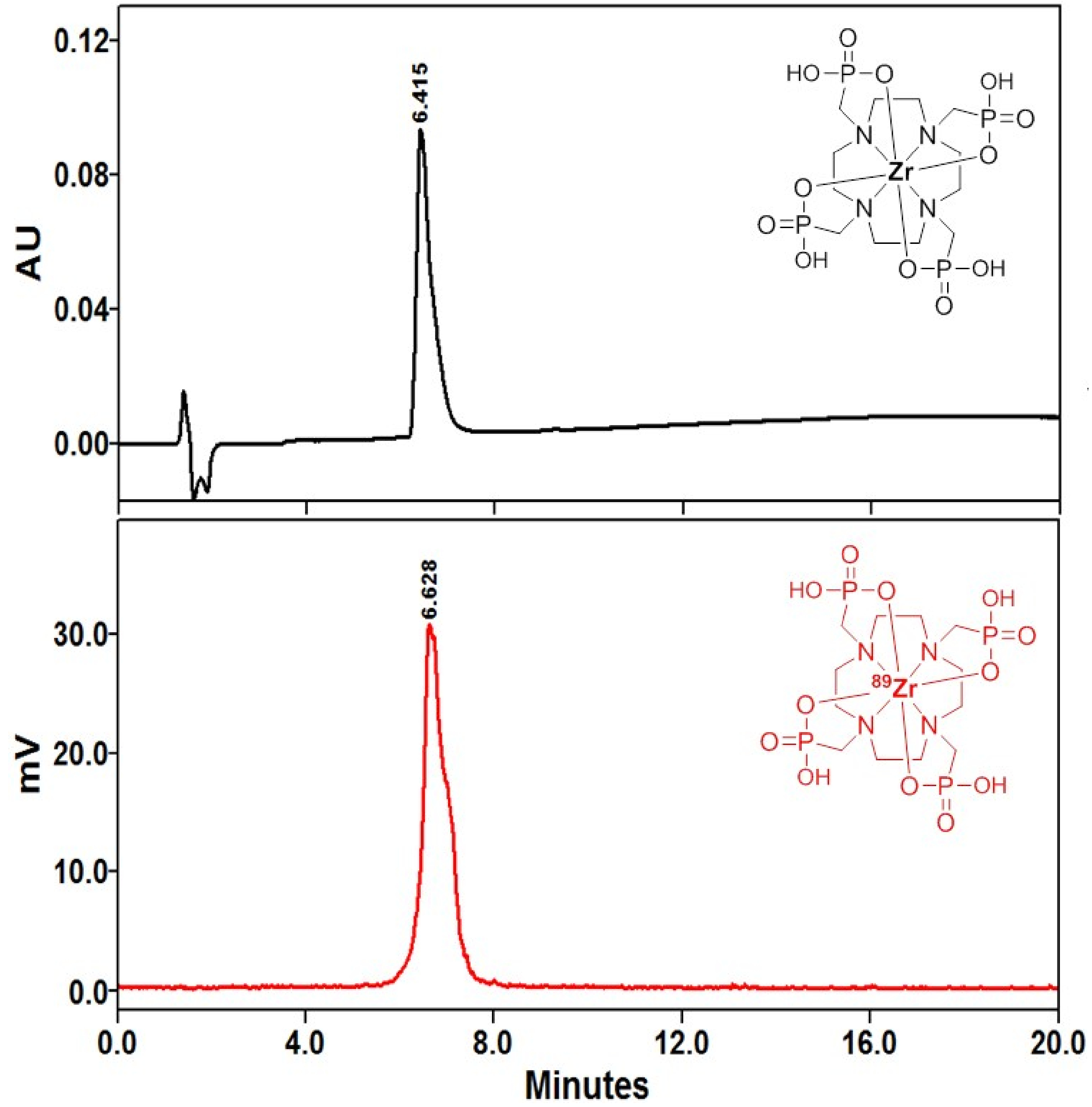

Figure S28. Quality control of ^{89}Zr-DOTP by radio-HPLC. UV-HPLC chromatogram (201 nm) of nonradioactive ^{Nat}Zr-DOTP complex (top) compared with radio-HPLC chromatogram of ^{89}Zr-DOTP (bottom).

^{89}Zr-DOTAM: Complexing ^{89}Zr with the tetraazamacrocyclic ligand DOTAM was achieved by reacting 10 µg (10 µL, 1.0 mg/mL in water) of the ligand DOTAM with an aliquot of $^{89}ZrCl_4$ (1.1 mCi, 40.7 MBq) diluted in 100 µL of 1 M HEPES (pH 7.2) and 10 µL of 1 % sodium dodecyl sulfate followed by 45 min incubation at 90ºC in a thermomixer (550 rpm). Formation of the ^{89}Zr-DOTAM complex was monitored by radio-TLC using Varian ITLC-SA strips and 0.1 M oxalic acid (pH 5) as the mobile phase. In this system, free ^{89}Zr formed a complex with oxalic acid and eluted with the solvent front (R_f = 1), while the ^{89}Zr-DOTAM complex remained at the origin (R_f = 0) (Fig. S29).

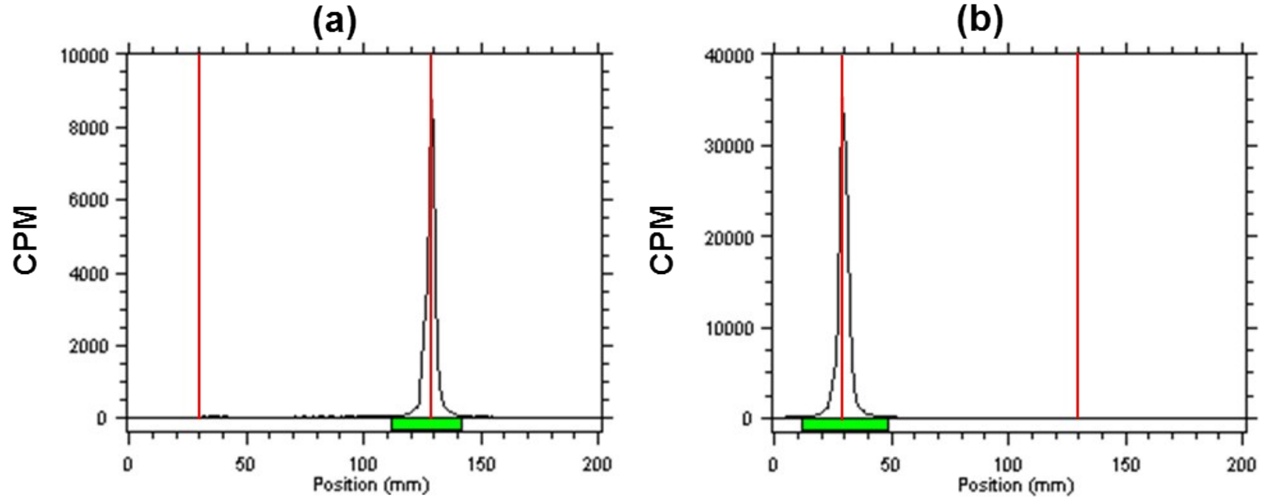

Figure S29. Quality control of ^{89}Zr-DOTAM by radio-TLC. ITLC of $^{89}ZrCl_4$ (a), and ^{89}Zr-DOTAM (b)

Table S7. Summary of optimized radiochemistry conditions to prepare ^{89}Zr-complexes with $^{89}ZrCl_4$

Radiochemistry conditions	Ligand (n = 50)			
	**DOTA**	**DOTP**	**DOTAM**	**DFO**
Quantity (μg)	10	10	10	10
Temperature (°C)	90	90	90	24
Reaction time (min)	45	45	45	15
Reaction buffer	0.5 M HEPES	-	1 M HEPES	-
Reaction pH	6.9-7.2	7.0-7.5	6.9-7.2	7.0-7.5
Radiochemical yield (%)	100	100	100	100
Specific activity (A_s; MBq μmol^{-1})	1010 ± 8	1008 ± 10	989 ± 10	1005 ± 10

***In vitro* EDTA challenge study**. *In vitro* EDTA challenge study was carried out by adding 10 µL of each ^{89}Zr-labeled complex (70 µCi, 2.59 MBq) to 500 µL of EDTA (10 mM, 50 mM and 100 mM: pH 5 and pH 7) with a 1:100, 1:500, and 1:1000 ratio of ligand/EDTA. The solutions (n=4) were incubated at 37 °C for 7 days in a thermomixer. Samples were analyzed at 30 min, 1 h, 3 h, 6 h, 1 d, 3 d, 5 d and 7 d post administration to EDTA by radio-TLC using Varian ITLC-SA strips and 0.1 M EDTA (pH 5) as the mobile phase and gamma counting using an energy window of 500-1500 keV and standard protocols .

Table S8. Stability of ^{89}Zr-complexes in EDTA (pH 5 and pH 7) in challenge study at 37 °C for 7 days

Complex	EDTA	pH	% Intact of ^{89}Zr-complexes (n = 4)							
			30 min	1 h	3 h	6 h	1 d	3 d	5 d	7 d
**^{89}Zr-DOTA**	100-fold	7.0		**100**			**100**	**100**	**100**	**100**
		5.0		**100**			**100**	**100**	**100**	**100**
	500-fold	7.0		**100**			**100**	**100**	**100**	**100**
		5.0		**100**			**100**	**100**	**100**	**100**
	1000-fold	7.0		**100**			**100**	**100**	**100**	**100**
		5.0		**100**			**100**	**100**	**100**	**100**
**^{89}Zr-DOTP**	100-fold	7.0		99.8 ± 0.1			98.6 ± 0.1	96.8 ± 0.3	94.5 ± 0.5	92.5 ± 0.4
		5.0		98.6 ± 0.3			91.8 ± 0.9	84.3 ± 0.7	78.6 ± 1.0	56.5 ± 1.4
	500-fold	7.0		99.5 ± 0.3			98.2 ± 0.1	94.9 ± 1.1	91.4 ± 0.3	89.3 ± 0.6
		5.0		97.4 ± 0.4			89.4 ± 0.7	72.4 ± 1.4	66.7 ± 0.7	53.8 ± 0.8
	1000-fold	7.0		99.2 ± 0.1			97.9 ± 0.1	91.5 ± 0.6	84.9 ± 0.6	79.4 ± 1.2
		5.0		96.3 ± 0.5			87.3 ± 1.2	67.5 ± 1.1	58.9 ± 1.4	43.7 ± 1.1

**^{89}Zr-DOTAM**	100-fold	7.0		99.0 ± 0.2			90.9 ± 0.8	86.1 ± 1.1	75.7 ± 0.8	72.1 ± 2.0
		5.0		97.7 ± 0.2			84.4 ± 0.9	67.6 ± 0.9	49.6 ± 0.6	42.3 ± 1.2
	500-fold	7.0		98.5 ± 0.2			90.8 ± 0.9	77.5 ± 1.4	66.6 ± 0.5	58.1 ± 1.4
		5.0		97.5 ± 0.6			80.0 ± 1.2	47.0 ± 0.4	22.8 ± 1.8	12.2 ± 1.5
	1000-fold	7.0		98.3 ± 0.1			88.6 ± 0.4	69.9 ± 1.8	58.2 ± 1.3	47.4 ± 0.9
		5.0		96.5 ± 0.2			77.0 ± 1.3	38.3 ± 1.5	13.4 ± 1.6	5.7 ± 1.0
**^{89}Zr-DFO**	100-fold	7.0	99.0 ± 0.4	96.6 ± 1.1	90.3 ± 0.8	89.4 ± 0.2	88.6 ± 0.2	87.2 ± 0.6	86.6 ± 0.3	83.9 ± 0.5
		5.0	93.0 ± 0.7	85.5 ± 0.9	59.6 ± 0.8	29.4 ± 0.7	6.6 ± 0.3	5.4 ± 0.3	4.4 ± 0.2	3.6 ± 0.1
	500-fold	7.0	90.3 ± 0.7	81.1 ± 1.1	58.6 ± 0.3	42.7 ± 1.2	36.2 ± 0.7	31.8 ± 1.1	30.4 ± 0.9	28.5 ± 0.4
		5.0	60.9 ± 1.0	33.9 ± 1.2	3.9 ± 0.4	0				
	1000-fold	7.0	85.6 ± 1.1	68.3 ± 1.2	45.4 ± 0.7	32.9 ± 0.5	28.1 ± 0.7	24.5 ± 0.8	21.4 ± 1.2	20.3 ± 0.5
		5.0	37.7 ± 1.1	14.6 ± 0.7	0						

***In vitro* Metal Competition study.** To a solution of metal cations [iron (III) chloride, cobalt (II) chloride, zinc (II) chloride, copper (II) chloride, magnesium (II) chloride, gallium (III) nitrate, gadolinium (III) chloride] (1 mM, 200 μL), was added ^{89}Zr-labeled complex (0.1 mM, 10 μL, 80 μCi, 2.96 MBq) in PBS, pH 7.4. The resulting solutions (n = 3) were incubated at 37ºC for 7 days in a thermomixer. Dissociation of ^{89}Zr from ^{89}Zr-complexes was monitored by radio-TLC at 2 h, 1 d, 4 d and 7 d time point. All studies were performed in triplicate.

Table S9. Stability of ^{89}Zr-complexes with various metals (PBS, pH 7.4) in competition study at 37 °C for 7 days

Time Point	Complex	% Intact of ^{89}Zr-complexes (n = 4)						
		Fe^{3+}	Zn^{2+}	Co^{2+}	Cu^{2+}	Mg^{2+}	Gd^{3+}	Ga^{3+}
2 h	**^{89}Zr-DOTA**	**100**	**100**	**100**	**100**	**100**	**100**	**100**
	**^{89}Zr-DOTP**	99.3 ± 0.1	99.0 ± 0.1	99.1 ± 0.2	98.4 ± 0.1	99.2 ± 0.2	99.5 ± 0.4	99.2 ± 0.1
	**^{89}Zr-DOTAM**	92.4 ± 0.5	94.1 ± 0.5	92.6 ± 0.5	91.9 ± 0.1	93.6 ± 1.0	93.9 ± 0.3	92.9 ± 0.7
	**^{89}Zr-DFO**	96.5 ± 0.2	99.5 ± 0.1	99.2 ± 0.2	98.9 ± 0.5	99.3 ± 0.1	99.4 ± 0.3	99.3 ± 0.2
1 d	**^{89}Zr-DOTA**	**100**	**100**	**100**	**100**	**100**	**100**	**100**
	**^{89}Zr-DOTP**	96.8 ± 1.2	95.4 ± 0.3	97.2 ± 0.1	95.4 ± 0.2	99.4 ± 0.1	97.9 ± 0.4	98.2 ± 0.2
	**^{89}Zr-DOTAM**	89.8 ± 0.2	89.4 ± 1.9	88.6 ± 0.3	86.7 ± 0.7	90.5 ± 0.5	90.6 ± 0.3	87.9 ± 0.7
	**^{89}Zr-DFO**	76.1 ± 1.3	98.8 ± 0.3	98.3 ± 0.4	98.6 ± 0.4	98.6 ± 0.5	98.8 ± 0.1	93.4 ± 2.6
4 d	**^{89}Zr-DOTA**	**100**	**100**	**100**	**100**	**100**	**100**	**100**
	**^{89}Zr-DOTP**	89.5 ± 0.7	89.4 ± 1.5	95.3 ± 0.5	97.5 ± 0.7	98.1 ± 0.1	93.2 ± 1.0	93.6 ± 0.6
	**^{89}Zr-DOTAM**	87.2 ± 0.8	71.9 ± 1.7	85.8 ± 0.4	80.5 ± 0.8	87.4 ± 0.9	88.8 ± 0.6	86.6 ± 2.3
	**^{89}Zr-DFO**	53.2 ± 3.8	97.7 ± 0.6	98.1 ± 0.3	97.3 ± 0.7	97.6 ± 0.8	98.4 ± 0.3	81.1 ± 0.5
7 d	**^{89}Zr-DOTA**	**100**	**100**	**100**	**100**	**100**	**100**	**100**
	**^{89}Zr-DOTP**	84.0 ± 0.4	84.7 ± 2.7	93.5 ± 1.3	94.4 ± 1.3	96.3 ± 0.4	91.7 ± 2.0	91.1 ± 1.4
	**^{89}Zr-DOTAM**	85.8 ± 0.8	57.0 ± 1.8	80.9 ± 1.0	74.1 ± 1.2	93.1 ± 0.8	86.3 ± 0.1	80.3 ± 0.4
	**^{89}Zr-DFO**	33.9 ± 1.5	95.9 ± 0.7	95.4 ± 0.9	96.0 ± 1.0	96.8 ± 1.2	96.8 ± 0.2	72.6 ± 1.3

***In vitro* serum stability**. *In vitro* serum stability was carried out by adding 10 µL of each ^{89}Zr-labeled complex (70 µCi, 2.59 MBq) to 500 µL of human serum. The solutions (n=4) were incubated at 37 °C for 7 days and were analyzed daily for 1 week by radio -TLC using Varian ITLC-SA strips and 0.1 M EDTA (pH 5) as the mobile phase and gamma counting using an energy window of 500-1500 keV and standard protocols . Serum samples were also analyzed after 7 days by size exclusion chromatography (SEC) using a Superdex 200 10/300 GL™ column (GE Healthcare Life Sciences, Piscataway, NJ) and phosphate buffered saline (PBS) as eluent with a flow rate of 0.5 mL/min.

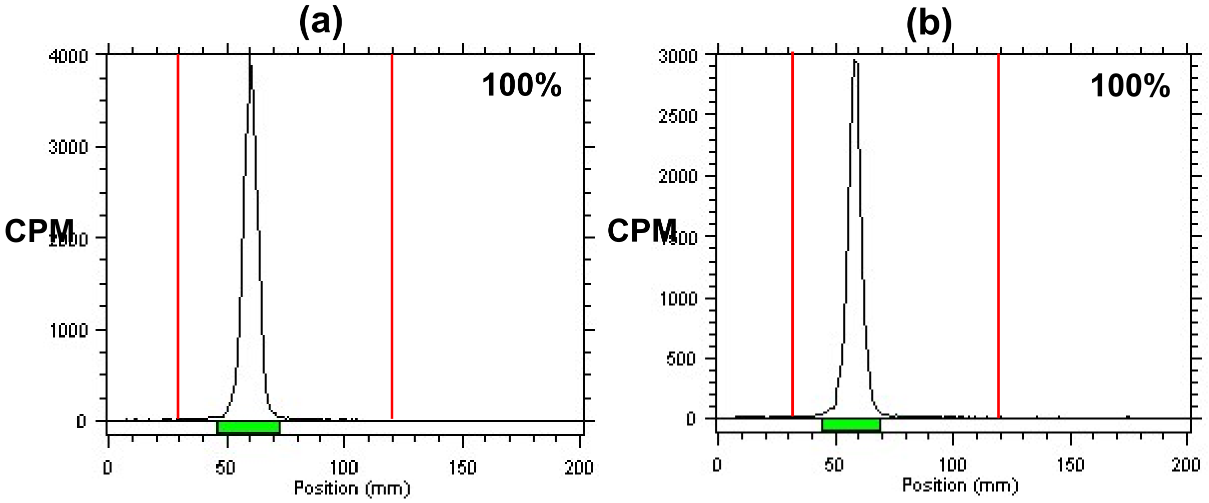

Figure S30. Radio-ITLC of ^{89}Zr-DOTA solution in human serum at 37 °C after 0 h (a) and 7 days (b).

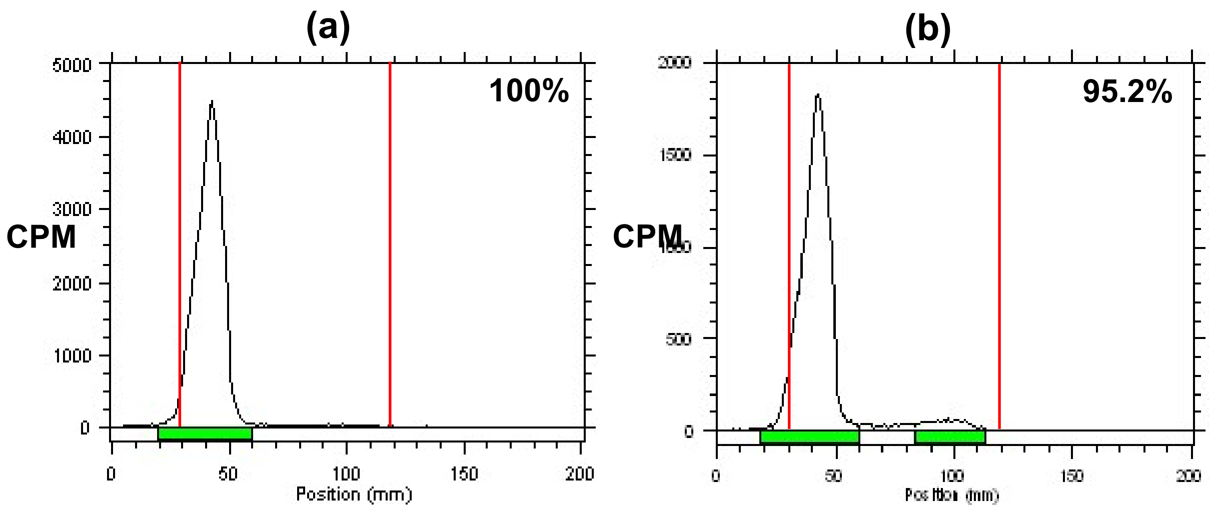

Figure S31. Radio-ITLC of ^{89}Zr-DOTP solution in human serum at 37 °C after 0 h (a) and 7 days (b).

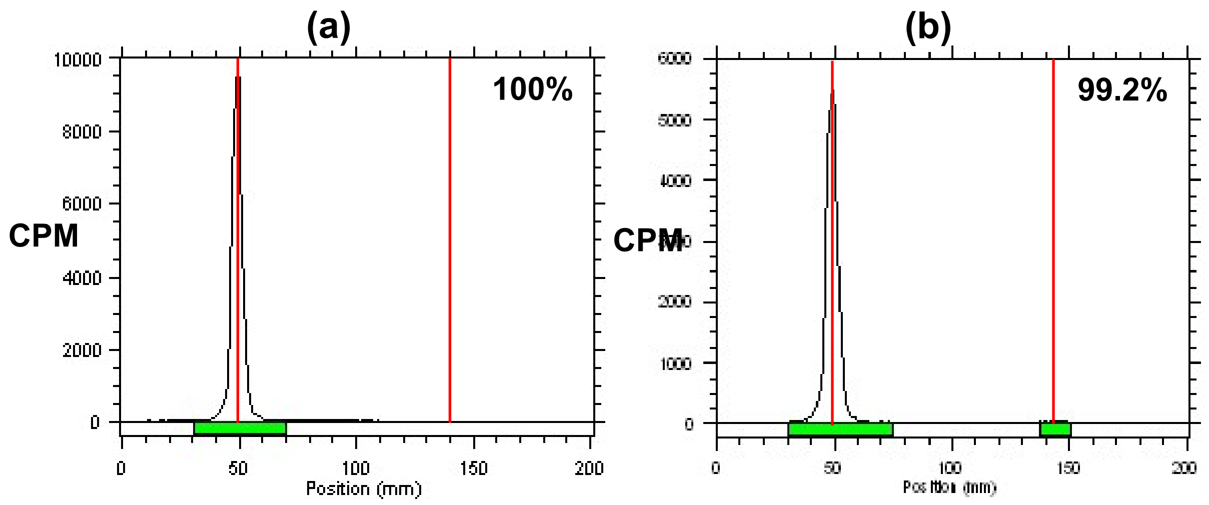

Figure S32. Radio-ITLC of ^{89}Zr-DOTAM solution in human serum at 37 °C after 0 h (a) and 7 days (b).

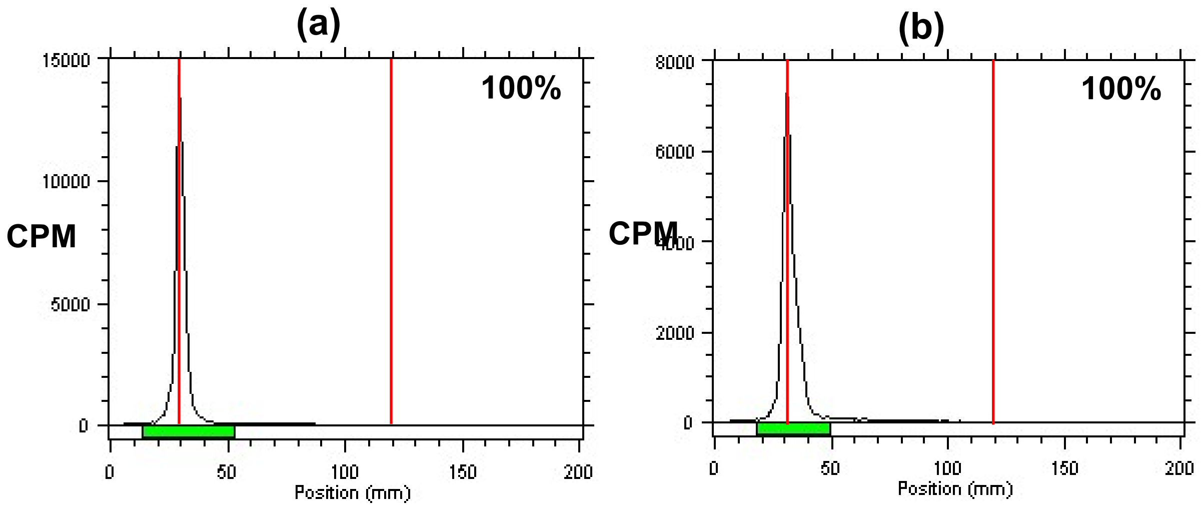

Figure S33. Radio-ITLC of ^{89}Zr-DFO solution in human serum at 37 °C after 0 h (a) and 7 days (b).

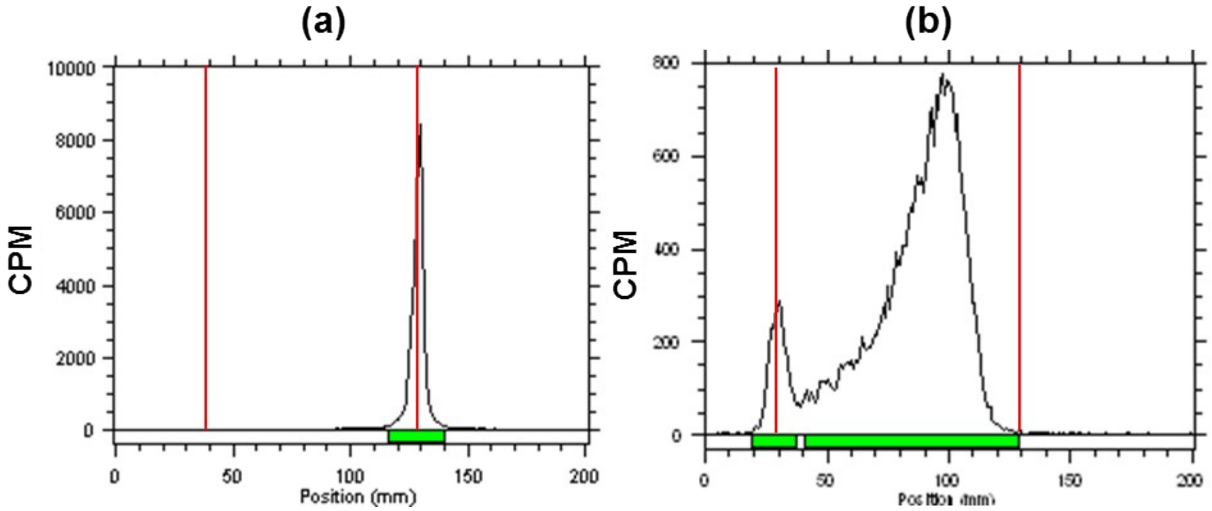

Figure S34. Radio-ITLC of $^{89}ZrCl_4$ solution in human serum at 37 °C after 0 h (a) and 7 days (b).

When samples of serum which contained unchelated ^{89}Zr were analyzed using radio-ITLC, we observed broad peaks ranging form the origin to the solvent front because unchelated ^{89}Zr could be bound to variety of serum protein components and migrate with different retention factors in our ITLC system. Therefore we elected to perform size exclusion chromatography to further characterize the stability of ^{89}Zr-complexes.

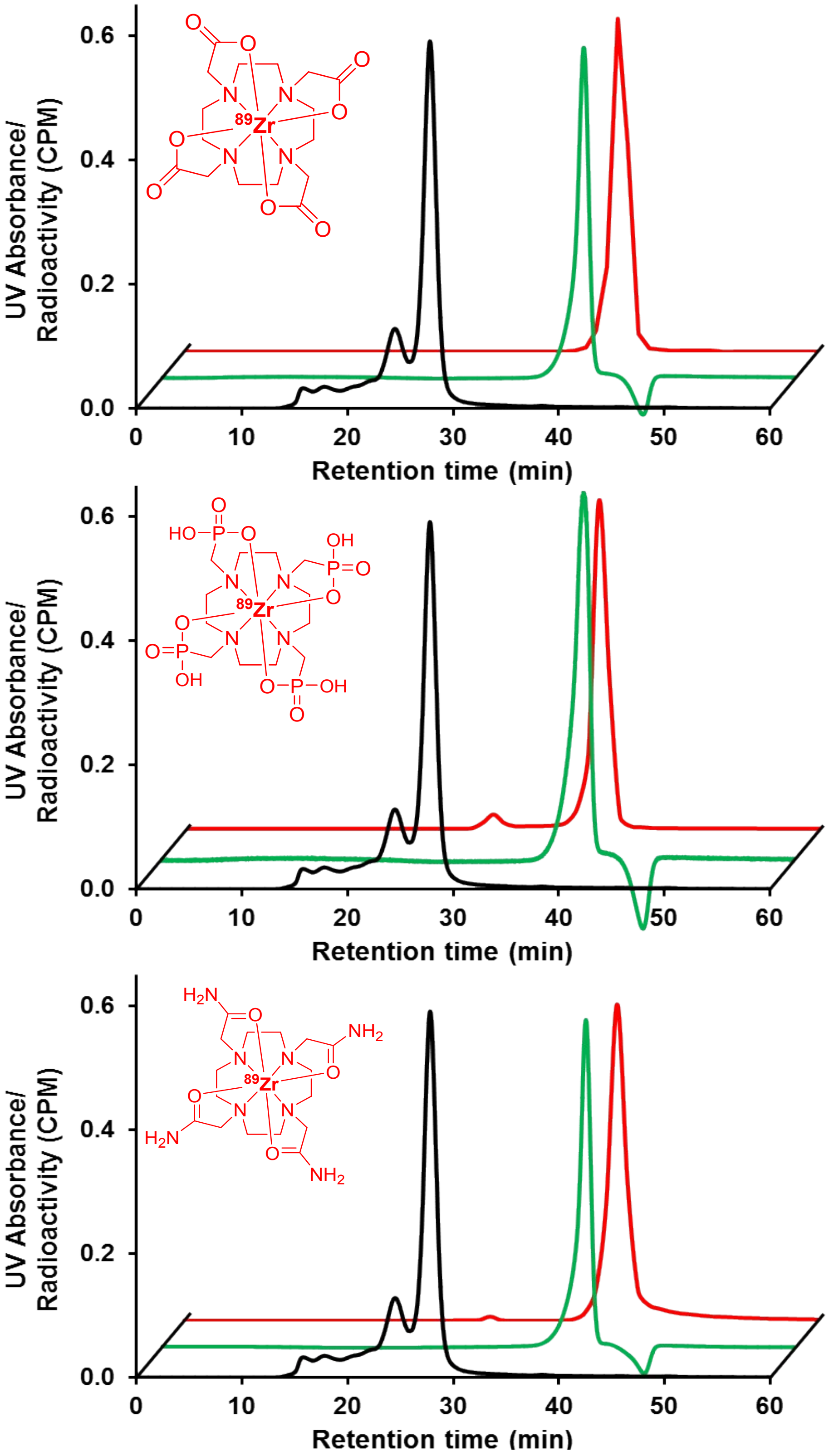

Figure S35. *In vitro* serum stability by HPLC. UV-SE-HPLC (220 nm, black, and green) and radio-SE-HPLC chromatogram (red) of ^{89}Zr-DOTA (top), ^{89}Zr-DOTP (middle) and ^{89}Zr-DOTAM (bottom) in serum after 7 days. Black lines are the UV absorbance due to the human serum components, green lines are the UV absorbance associated with ^{Nat}Zr-complexes and red lines are the radiotracer associated with ^{89}Zr-complexes.

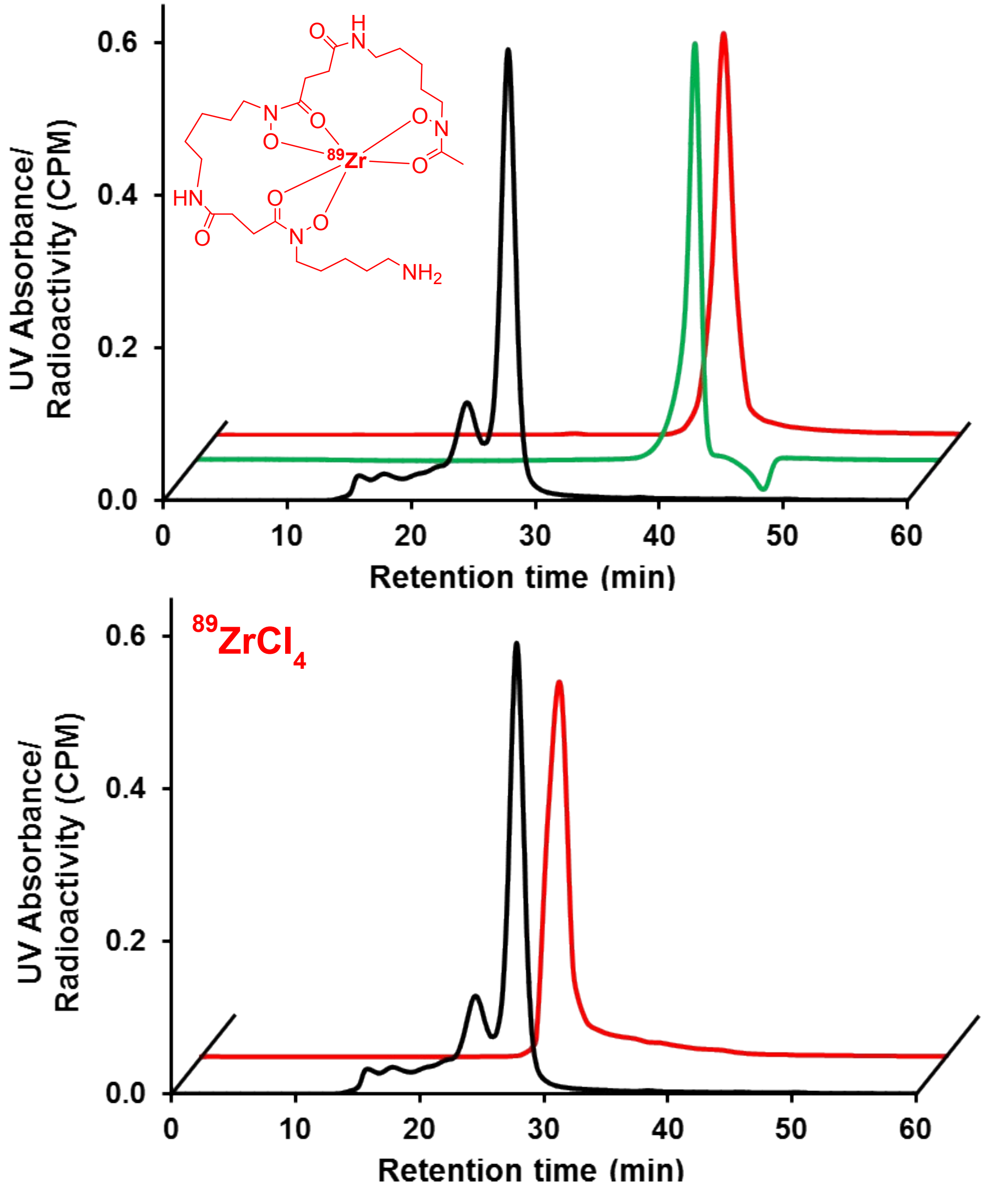

Figure S36. ***In vitro* serum stability by HPLC.** UV-SE-HPLC (220 nm, black, and green) and radio-SE-HPLC chromatogram (red) of ^{89}Zr-DFO (top), and $^{89}ZrCl_4$ (bottom) in serum after 7 days. Black lines are the UV absorbance due to the human serum components, green lines are the UV absorbance associated with ^{Nat}Zr-complexes and red lines are the radiotracer associated with ^{89}Zr-complexes.

Table S10. Summary of *in vitro* serum stability of ^{89}Zr-complexes in human serum at 37 °C for 7 days

Day	^{89}Zr-DOTA (%)		^{89}Zr-DOTP (%)		^{89}Zr-DOTAM (%)		^{89}Zr-DFO (%)	
	Radio-ITLC	Radio-HPLC	Radio-ITLC	Radio-HPLC	Radio-ITLC	Radio-HPLC	Radio-ITLC	Radio-HPLC
1	100	-	100	-	100	-	100	-
3	100	-	97.6 ± 0.2	-	100	-	100	-
5	100	-	95.9 ± 0.4	-	99.8 ± 0.1	-	100	-
7	100	100	95.1 ± 0.5	95.6 ± 0.2	99.1 ± 0.3	99.3 ± 0.4	100	99.8 ± 0.1

Determination of partition coefficients (logP).[6] The partition coefficient (logP) for each complex was determined by adding 5 μL of each ^{89}Zr-labeled complex (approx. 5 μCi; 0.19 MBq) to a mixture of 500 μL of octanol and 500 μL of water. The resulting solutions (n = 5) were vigorously vortexed for 5 min at room temperature, then centrifuged for 5 min to ensure complete separation of layers. From each of the five sets, 50 μL aliquot was removed from each phase into screw tubes and counted separately in a gamma counter. Each organic phase was washed with water to remove any radioactivity remaining in the organic phase before gamma counting. The partition coefficient was calculated as a ratio of counts in the octanol fraction to counts in the water fraction. The logP values were reported in an average of five measurements.

Table S11. Log P values for all ^{89}Zr-complexes

Complex	log P (n = 5)
^{89}Zr-DOTA	-3.80 ± 0.04
^{89}Zr-DOTP	-3.89 ± 0.02
^{89}Zr-DOTAM	-1.40 ± 0.03
^{89}Zr-DFO	-2.83 ± 0.04

Biodistribution Studies. Biodistribution studies were conducted using a modified literature procedure.[7] Briefly, female NIH Swiss mice (6-8 wk old, n=6) were injected with each ^{89}Zr-labeled complex (0.55 MBq (15 μCi)/mouse) via the tail vein, and sacrificed at 2, 4, 24, 48, 72 h post-injection. Organs and tissues of interest were excised, weighted, and counted on a Perkin Elmer 2480 Wizard® gamma counter (Waltham, MA). The percent injected dose per gram (%ID/g) and percent injected dose per organ (%ID/organ) were calculated by comparison to a weighed, counted standard for each group (Tables S9 – S12).

Statistical Methods. All of the data are presented as mean±SD or mean (95% Confidence Interval). For statistical classification a student's t test (two-tailed, unpaired) was performed using GraphPad Prism (San Diego, CA). Any $p<0.05$ was considered significant.

Table S12. Biodistribution (%ID/g) of ^{89}Zr-DOTA in selected organs at 2, 4, 24, 48, and 72 h p.i.

Tissue/Organ	2 h	4 h	24 h	48 h	72 h
Blood	0.010 ± 0.003	0.003 ± 0.002	0.001 ± 0.000	0.001 ± 0.000	0.000 ± 0.001
Heart	0.024 ± 0.008	0.020 ± 0.006	0.016 ± 0.004	0.015 ± 0.005	0.009 ± 0.004
Lung	0.069 ± 0.007	0.053 ± 0.008	0.025 ± 0.007	0.023 ± 0.004	0.014 ± 0.001
Liver	0.134 ± 0.017	0.123 ± 0.013	0.068 ± 0.004	0.035 ± 0.003	0.021 ± 0.002
Small intestine	0.311 ± 0.274	0.047 ± 0.008	0.033 ± 0.003	0.031 ± 0.006	0.022 ± 0.006
Large intestine	0.494 ± 0.185	0.281 ± 0.080	0.199 ± 0.055	0.189 ± 0.034	0.152 ± 0.051
Kidney	1.137 ± 0.163	0.757 ± 0.031	0.317 ± 0.054	0.131 ± 0.015	0.078 ± 0.009
Spleen	0.048 ± 0.004	0.042 ± 0.011	0.041 ± 0.004	0.029 ± 0.009	0.018 ± 0.008
Pancreas	0.015 ± 0.003	0.022 ± 0.007	0.009 ± 0.002	0.005 ± 0.002	0.003 ± 0.002
Stomach	0.073 ± 0.049	0.021 ± 0.006	0.027 ± 0.006	0.027 ± 0.009	0.024 ± 0.011
Muscle	0.007 ± 0.001	0.006 ± 0.002	0.004 ± 0.001	0.003 ± 0.002	0.002 ± 0.001
Fat	0.008 ± 0.003	0.008 ± 0.003	0.006 ± 0.004	0.005 ± 0.001	0.002 ± 0.001
Bone	0.027 ± 0.008	0.023 ± 0.005	0.036 ± 0.008	0.024 ± 0.005	0.025 ± 0.009

Table S13. Biodistribution (%ID/g) of ^{89}Zr-DOTP in selected organs at 2, 4, 24, 48, and 72 h p.i.

Tissue/Organ	2 h	4 h	24 h	48 h	72 h
Blood	0.108 ± 0.022	0.039 ± 0.009	0.006 ± 0.002	0.003 ± 0.001	0.001 ± 0.001
Heart	0.048 ± 0.010	0.029 ± 0.003	0.016 ± 0.004	0.013 ± 0.003	0.010 ± 0.002
Lung	0.095 ± 0.010	0.071 ± 0.009	0.036 ± 0.005	0.023 ± 0.006	0.022 ± 0.002
Liver	0.064 ± 0.008	0.059 ± 0.009	0.052 ± 0.009	0.040 ± 0.003	0.036 ± 0.002
Small intestine	0.179 ± 0.050	0.054 ± 0.011	0.033 ± 0.007	0.027 ± 0.007	0.023 ± 0.007
Large intestine	0.235 ± 0.064	0.420 ± 0.048	0.136 ± 0.041	0.121 ± 0.028	0.100 ± 0.014
Kidney	1.037 ± 0.114	0.816 ± 0.115	0.609 ± 0.036	0.395 ± 0.011	0.316 ± 0.045
Spleen	0.059 ± 0.004	0.038 ± 0.006	0.033 ± 0.007	0.028 ± 0.003	0.025 ± 0.003
Pancreas	0.031 ± 0.006	0.023 ± 0.005	0.014 ± 0.005	0.013 ± 0.004	0.011 ± 0.002
Stomach	0.077 ± 0.027	0.027 ± 0.008	0.025 ± 0.009	0.020 ± 0.009	0.017 ± 0.006
Muscle	0.020 ± 0.007	0.016 ± 0.005	0.006 ± 0.002	0.008 ± 0.004	0.004 ± 0.001
Fat	0.021 ± 0.007	0.012 ± 0.003	0.006 ± 0.004	0.007 ± 0.003	0.005 ± 0.003
Bone	4.814 ± 0.745	3.364 ± 0.301	2.622 ± 0.433	2.373 ± 0.732	2.631 ± 0.124

Table S14. Biodistribution (%ID/g) of ^{89}Zr-DOTAM in selected organs at 2, 4, 24, 48, and 72 h p.i.

Tissue/Organ	2 h	4 h	24 h	48 h	72 h
Blood	0.533 ± 0.134	0.373 ± 0.097	0.041 ± 0.013	0.023 ± 0.010	0.015 ± 0.009
Heart	0.907 ± 0.252	1.464 ± 0.420	1.255 ± 0.236	1.128 ± 0.288	1.148 ± 0.342
Lung	2.865 ± 0.573	4.397 ± 1.093	2.640 ± 0.632	2.127 ± 0.489	2.158 ± 0.364
Liver	43.788 ± 7.980	52.116 ± 4.435	53.649 ± 5.774	54.116 ± 3.848	61.826 ± 2.268
Small intestine	0.139 ± 0.018	0.144 ± 0.031	0.150 ± 0.033	0.225 ± 0.043	0.183 ± 0.036
Large intestine	0.049 ± 0.010	0.064 ± 0.010	0.090 ± 0.035	0.100 ± 0.014	0.119 ± 0.034
Kidney	1.276 ± 0.312	1.632 ± 0.155	1.326 ± 0.237	1.926 ± 0.100	1.697 ± 0.166
Spleen	58.831 ± 5.687	58.365 ± 9.312	82.889 ± 10.725	56.220 ± 15.581	60.382 ± 8.866
Pancreas	0.146 ± 0.068	0.115 ± 0.022	0.102 ± 0.013	0.139 ± 0.032	0.153 ± 0.028
Stomach	0.063 ± 0.029	0.171 ± 0.113	0.054 ± 0.022	0.061 ± 0.016	0.083 ± 0.012
Muscle	0.083 ± 0.037	0.047 ± 0.008	0.051 ± 0.012	0.105 ± 0.021	0.090 ± 0.030
Fat	0.086 ± 0.068	0.046 ± 0.009	0.041 ± 0.008	0.050 ± 0.026	0.099 ± 0.030
Bone	3.157 ± 0.788	4.145 ± 0.860	3.700 ± 0.644	6.831 ± 0.536	6.055 ± 0.802

Table S15. Biodistribution (%ID/g) of ^{89}Zr-DFO in selected organs at 2, 4, 24, 48, and 72 h p.i.[8]

Tissue/Organ	2 h	4 h	24 h	48 h	72 h
Blood	0.009 ± 0.003	0.005 ± 0.001	0.001 ± 0.001	0.001 ± 0.001	0.000 ± 0.001
Heart	0.020 ± 0.003	0.019 ± 0.003	0.014 ± 0.002	0.010 ± 0.002	0.009 ± 0.004
Lung	0.060 ± 0.009	0.038 ± 0.006	0.024 ± 0.006	0.019 ± 0.005	0.017 ± 0.004
Liver	0.234 ± 0.023	0.163 ± 0.051	0.081 ± 0.012	0.070 ± 0.007	0.066 ± 0.009
Small intestine	0.357 ± 0.175	0.130 ± 0.080	0.013 ± 0.002	0.008 ± 0.001	0.006 ± 0.001
Large intestine	0.877 ± 0.435	1.020 ± 0.207	0.024 ± 0.004	0.009 ± 0.002	0.008 ± 0.001
Kidney	2.051 ± 0.238	1.848 ± 0.382	1.340 ± 0.137	0.957 ± 0.216	0.689 ± 0.098
Spleen	0.037 ± 0.005	0.036 ± 0.004	0.036 ± 0.007	0.030 ± 0.008	0.027 ± 0.007
Pancreas	0.015 ± 0.005	0.013 ± 0.002	0.012 ± 0.002	0.009 ± 0.003	0.007 ± 0.002
Stomach	0.140 ± 0.124	0.055 ± 0.038	0.014 ± 0.005	0.005 ± 0.003	0.005 ± 0.002
Muscle	0.011 ± 0.001	0.008 ± 0.003	0.006 ± 0.002	0.004 ± 0.001	0.004 ± 0.002
Fat	0.013 ± 0.003	0.009 ± 0.002	0.007 ± 0.002	0.005 ± 0.008	0.008 ± 0.004
Bone	0.051 ± 0.017	0.058 ± 0.008	0.082 ± 0.016	0.092 ± 0.011	0.078 ± 0.014

[*]These data were published previously.[8]

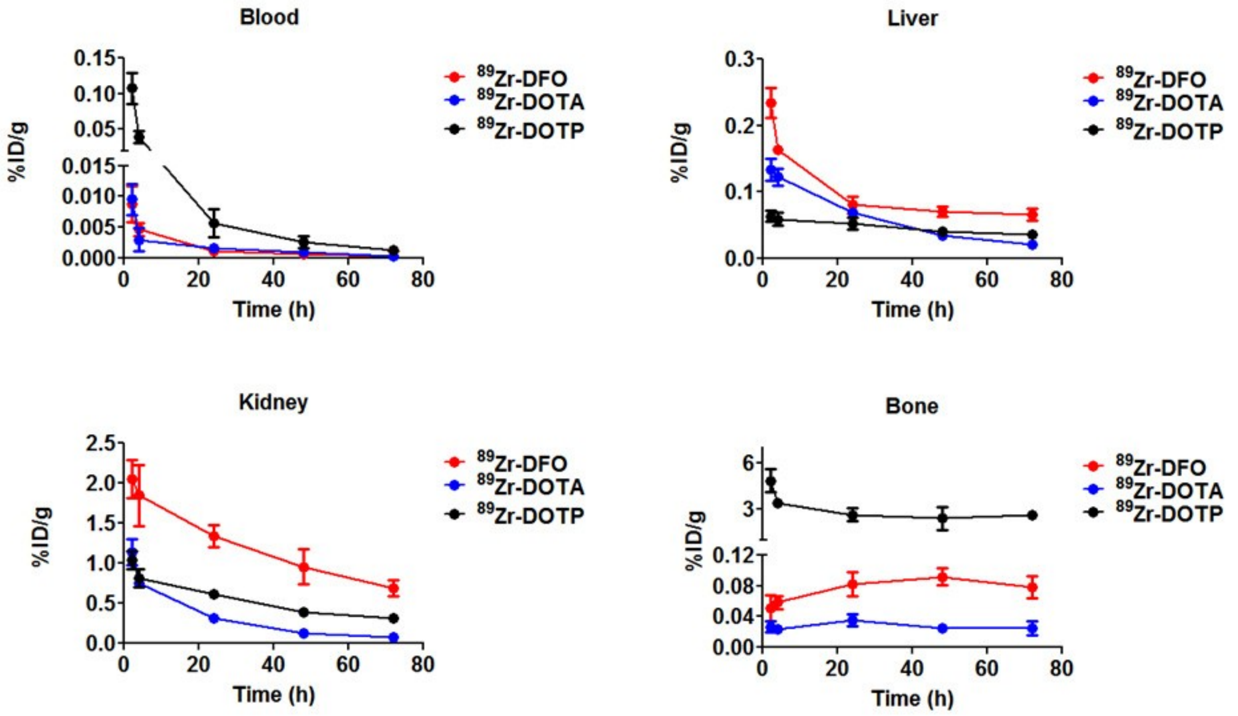

Figure S37. Biodistribution data summary of ^{89}Zr-DFO, ^{89}Zr-DOTA and ^{89}Zr-DOTP in selected tissues. Despite the elevated bone uptake associated with ^{89}Zr-DOTP, it demonstrates better clearance from the liver and kidney than ^{89}Zr-DFO. ^{89}Zr-DOTA demonstrated a superior clearance and excretion pattern when compared to all complexes studied and suggests this complex exhibits extraordinary stability *in vivo*.

PET/CT Imaging. PET imaging with ^{89}Zr-DFO or ^{89}Zr-DOTA was performed on healthy female NIH Swiss mice (6-8 wk old, n=6) using a small animal PET/CT scanner (eXplore VISTA model, GE Healthcare, Waukesha, WI). Before imaging, each mouse was anesthetized by inhalation of isoflurane mixed with oxygen gas (3% isoflurane for induction and 1-2% for maintenance) and a tail vein catheter was placed on the tail. The mouse was then positioned in the imaging cradle and a CT scan was performed for the subsequent attenuation correction and anatomical colocalization. After the CT scan, ^{89}Zr-DFO or ^{89}Zr-DOTA (11.1-12.5 MBq (300-338 μCi) in 100 μL saline/ mouse) was administered through the tail vein catheter, and a dynamic PET scan was simultaneously initiated and acquired for 1 hour to record the initial distribution and pharmacokinetics. Mice were re-anesthetized at 2, 4 and 24 hours, and a static PET scan was acquired for 20 min as late-phase scans. PET images were reconstructed using 2D ordered subset expectation maximization (2D-OSEM) algorithms with random, scatter, and attenuation corrections and then coregistered with the CT image. The PET system was calibrated using a Zr-89 phantom with known activity. Standard uptake value (SUV = [(nCi/mL) x (animal wt. (g))/ injected dose (nCi)]) and the measure of %ID/g was then calculated voxel-wise with the calibration factor and normalization to the injected dose and animal body weight. For dynamic scans, data were binned into 19 time frames with the following schemes: 6x10s, 2x30s, 3x60s, 5x5min, 3x10min. The tissue uptake curves were generated from the dynamic scans with regions of interest (ROIs) manually placed in heart, liver, kidney, bone, and muscle. Biodistribution data at 2, 4 and 24 hours were obtained from the PET images, with regions of interest placed in corresponding organs or tissues.

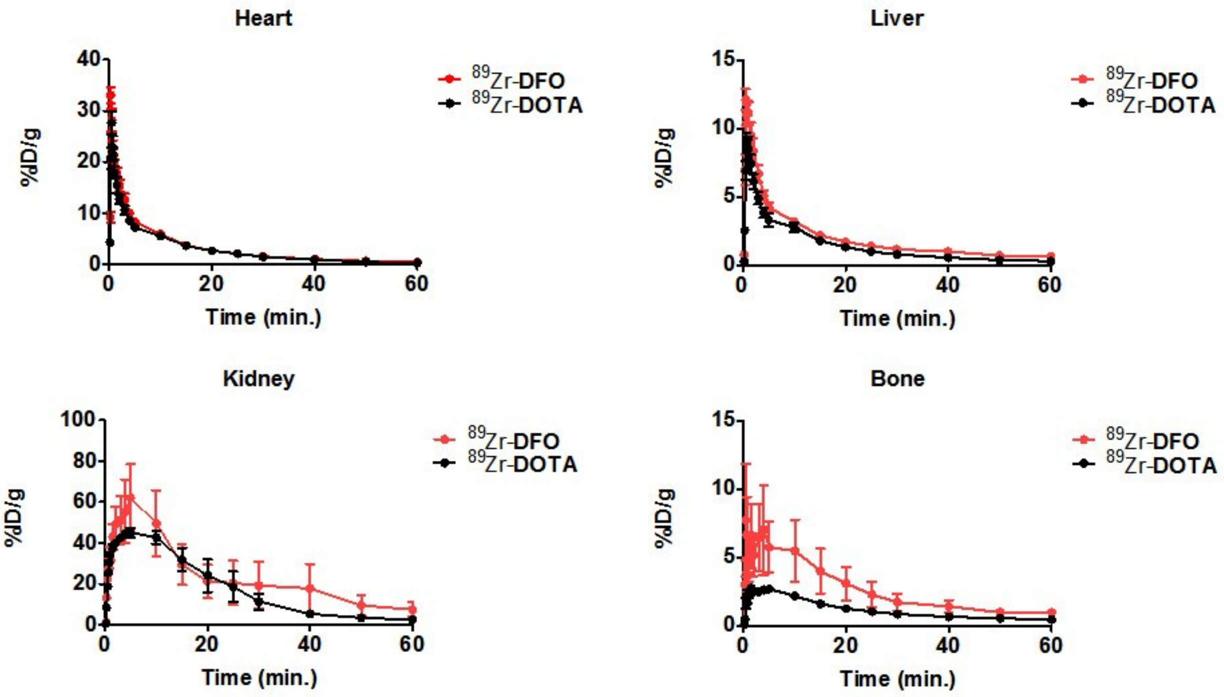

Figure S38. Dynamic PET data comparing ^{89}Zr-DFO and ^{89}Zr-DOTA in specific tissues. Both radiotracers behaved similarly during the first 60 minutes after injection into normal mice. Higher levels of radioactivity were observed in the kidney and bone tissue of mice receiving ^{89}Zr-DFO.

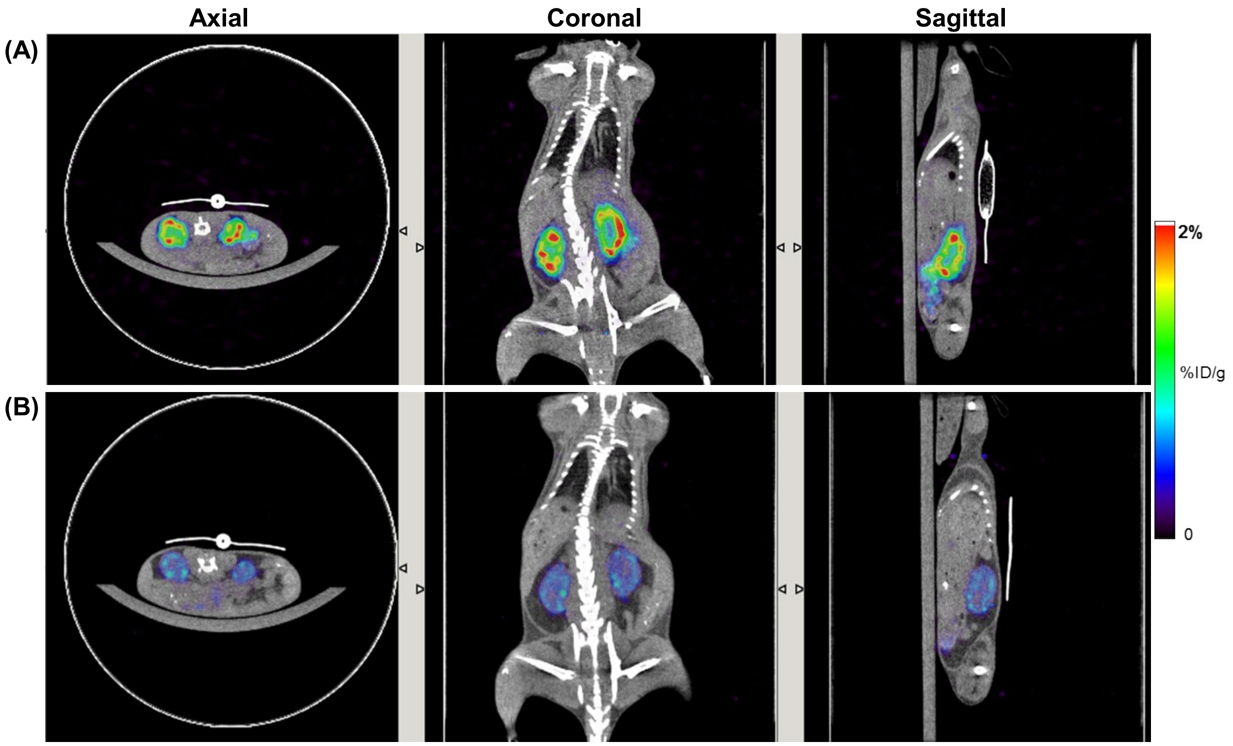

Figure S39. Co-registered PET/CT image results of ^{89}Zr-complexes at 4 h post-injection (p.i.) in normal mice. (**A**) Representative images of a mouse receiving ^{89}Zr-DFO. (**B**) Representative images of a mouse receiving ^{89}Zr-DOTA. PET images at 4 hours post injection reveal much lower kidney retention of ^{89}Zr-DOTA compared to that of ^{89}Zr-DFO. These data corroborate the biodistribution studies and the time activity data collected from 0-60 minutes.

Table S16. Image-based biodistribution in selected tissues (mean %ID/g ± SD) of normal mice (n =6/cohort) receiving ^{89}Zr-DFO or ^{89}Zr-DOTA

Tissue/Organ	2 h		4 h		24 h	
	^{89}Zr-DFO	^{89}Zr-DOTA	^{89}Zr-DFO	^{89}Zr-DOTA	^{89}Zr-DFO	^{89}Zr-DOTA
Heart	0.416 ± 0.141	0.272 ± 0.124	0.031 ± 0.017	0.030 ± 0.014	0.022 ± 0.012	0.019 ± 0.013
Liver	0.650 ± 0.100	0.321 ± 0.104	0.220 ± 0.078	0.125 ± 0.047	0.135 ± 0.047	0.073 ± 0.022
Kidney	3.717 ± 1.597	2.582 ± 1.034	1.894 ± 0.353	0.736 ± 0.305	1.414 ± 0.173	0.331 ± 0.138
Muscle	0.320 ± 0.287	0.964 ± 0.565	0.023 ± 0.012	0.013 ± 0.008	0.017 ± 0.007	0.011 ± 0.005
Bone	0.998 ± 0.525	0.469 ± 0.278	0.058 ± 0.022	0.023 ± 0.008	0.070 ± 0.034	0.008 ± 0.005

Table S17. Comparative stability of ^{89}Zr-Complexes in exogenous ligand challenge study

Complex	Challenging Ligand	pH	Time Point	% Intact of ^{89}Zr-complexes
^{89}Zr-L4[9]	EDTA (1000-fold)	7.0	6 d	87 ± 1
^{89}Zr-TAM-1[8]	DTPA (1000-fold)	7.0	7 d	100
^{89}Zr-TAM-2[8]	DTPA (1000-fold)	7.0	7 d	100
^{89}Zr-2,3-HOPO[10]	DTPA (1000-fold)	7.0	7 d	78
^{89}Zr-C7[11]	EDTA (1750-fold)	7.0	7 d	87 ± 3
^{89}Zr-CP256[12]	Data Not Reported			
^{89}Zr-TAFC[13]	EDTA (1000-fold)	7.0	7 d	97.2 ± 0.2
		6.0	7 d	94.4 ± 0.5
^{89}Zr-DFO*[14]	Data Not Reported			
^{89}Zr-DFOSq-Taur[15]	EDTA (500-fold)	7.0	1 d	88 ± 3.2
^{89}Zr-(oxinate)$_4$[16]	Data Not Reported			
^{89}Zr-(Me-AHA)$_4$[17]	Data Not Reported			
^{89}Zr-HOPO[18]	EDTA (100-fold)	7.0	7 d	100
		5.0	7 d	99.2 ± 1.5
^{89}Zr-DOTA (Current Study)	EDTA (1000-fold)	7.0	7 d	100
		5.0	7 d	100

Table S18. Comparative stability of ^{89}Zr-Complexes with various metals in competition study

Complex	Time Point	% Intact of ^{89}Zr-complexes						
		Fe^{3+}	Zn^{2+}	Co^{2+}	Cu^{2+}	Mg^{2+}	Gd^{3+}	Ga^{3+}
^{89}Zr-L4[9]		Data Not Reported						
^{89}Zr-TAM-1[8]		Data Not Reported						
^{89}Zr-TAM-2[8]		Data Not Reported						
^{89}Zr-2,3-HOPO[10]		Data Not Reported						
^{89}Zr-C7[11]		Data Not Reported						
^{89}Zr-CP256[12]	20 min	14	Data Not Reported					
^{89}Zr-TAFC[13]		Data Not Reported						
^{89}Zr-DFO*[14]		Data Not Reported						
^{89}Zr-DFOSq-Taur[15]		Data Not Reported						
^{89}Zr-(oxinate)$_4$[16]		Data Not Reported						
^{89}Zr-(Me-AHA)$_4$[17]		Data Not Reported						
^{89}Zr-HOPO[18]	7 d	83.0±4.2	98.3±2.4	98.9±1.6	98.4±2.3	98.7±1.8	94.5±4.1	96.4±0.6
^{89}Zr-DOTA (Current Study)	7 d	100	100	100	100	100	100	100

Table S19. Comparative biodistribution results of ^{89}Zr-Complexes

Complex	Time Point	% ID/g					
		Blood	Heart	Liver	Kidney	Muscle	Bone
^{89}Zr-L4[9]	24 h	0.09±0.01	0.03±0.02	0.40±0.14	2.76±0.40	0.00±0.00	0.60±0.19
^{89}Zr-TAM-1[8]	24 h	0.003±0.002	0.046±0.007	0.449±0.037	8.214±1.018	0.016±0.007	0.100±0.030
^{89}Zr-TAM-2[8]	24 h	0.010±0.003	0.147±0.040	1.244±0.180	46.095±7.788	0.039±0.014	0.274±0.100
^{89}Zr-2,3-HOPO[10]	24 h	0.004±0.001	0.027± .006	0.650±0.080	29.191±6.989	0.007±0.016	0.272±0.066
^{89}Zr-C7[11]		Data Not Reported					
^{89}Zr-CP256[12]		Data Not Reported					
^{89}Zr-TAFC[13]	6 h	0.05±0.01			0.86±0.48		0.04±0.02
^{89}Zr-DFO*[14]		Data Not Reported					
^{89}Zr-DFOSq-Taur[15]		Data Not Reported					
^{89}Zr-(oxinate)$_4$[16]		Data Not Reported					
^{89}Zr-(Me-AHA)$_4$[17]		Data Not Reported					
^{89}Zr-HOPO[18]	24 h	0.02±0.00	0.07±0.01	0.06±0.03	0.51±0.29	0.06±0.01	0.17±0.03
^{89}Zr-DOTA (Current Study)	24 h	0.001±0.000	0.016±0.004	0.068±0.004	0.317±0.054	0.004±0.001	0.036±0.008

REFERENCES

1. I. Bruker AXS, 2014.

2. G. Sheldrick, 2014/5.

3. I. Bruker AXS, 2014.

4. I. Bruker AXS, 2002.

5. I. Bruker AXS, 2014.

6. R. N. Waterhouse, *Mol Imaging Biol*, 2003, **5**, 376-389.

7. T. J. Wadas, C. D. Sherman, J. H. Miner, J. R. Duncan and C. J. Anderson, *Magn Reson Med*, 2010, **64**, 1274-1280.

8. D. N. Pandya, S. Pailloux, D. Tatum, D. Magda and T. J. Wadas, *Chem Commun*, 2015, **51**, 2301-2303.

9. E. Boros, J. P. Holland, N. Kenton, N. Rotile and P. Caravan, *Chempluschem*, 2016, **81**, 274-281.

10. J. N. Tinianow, D. N. Pandya, S. L. Pailloux, A. Ogasawara, A. N. Vanderbilt, H. S. Gill, S.-P. Williams, T. J. Wadas, D. Magda and J. Marik, *Theranostics*, 2016, **6**, 511-521.

11. F. Guerard, Y.-S. Lee and M. W. Brechbiel, *Chem. - Eur. J.*, 2014, **20**, 5584-5591.

12. M. T. Ma, L. K. Meszaros, B. M. Paterson, D. J. Berry, M. S. Cooper, Y. Ma, R. C. Hider and P. J. Blower, *Dalton Trans.*, 2015, **44**, 4884-4900.

13. C. Zhai, D. Summer, C. Rangger, G. M. Franssen, P. Laverman, H. Haas, M. Petrik, R. Haubner and C. Decristoforo, *Mol. Pharmaceutics*, 2015, **12**, 2142-2150.

14. M. Patra, A. Bauman, C. Mari, C. A. Fischer, O. Blacque, D. Haussinger, G. Gasser and T. L. Mindt, *Chem. Commun.*, 2014, **50**, 11523-11525.

15. S. E. Rudd, P. Roselt, C. Cullinane, R. J. Hicks and P. S. Donnelly, *Chem. Commun.*, 2016, **52**, 11889-11892.

16. T. J. Ferris, P. Charoenphun, L. K. Meszaros, G. E. D. Mullen, P. J. Blower and M. J. Went, *Dalton Trans.*, 2014, **43**, 14851-14857.

17. F. Guerard, Y. S. Lee, R. Tripier, L. P. Szajek, J. R. Deschamps and M. W. Brechbiel, *Chem Commun.*, 2013, **49**, 1002-1004.

18. M. A. Deri, S. Ponnala, B. M. Zeglis, G. Pohl, J. J. Dannenberg, J. S. Lewis and L. C. Francesconi, *J Med Chem*, 2014, **57**, 4849-4860.
